# The effects of biofeedback training on athletes’ mental health and performance: a systematic review and Bayesian meta-analysis

**DOI:** 10.3389/fpsyg.2025.1662868

**Published:** 2025-10-21

**Authors:** Xuda Zhang, Zhizhao Chang, Shiao Zhao, Ziheng Ning

**Affiliations:** ^1^Faculty of Health Sciences and Sports, Macao Polytechnic University, Macau, Macao SAR, China; ^2^Sports Teaching and Research Department, Lanzhou University, Lanzhou, China

**Keywords:** biofeedback, neurofeedback, mental health, athletic performance, Bayesian meta-analysis

## Abstract

**Background:**

Biofeedback and neurofeedback are increasingly used in sports psychology, yet their overall effectiveness for athletes’ mental health, athletic performance, and cognitive performance remains unclear.

**Methods:**

We conducted a systematic review of randomized controlled trials across eight databases and performed Bayesian random-effects meta-analyses. Study selection used ASReview with the SAFE rule; full-text screening was done in Covidence; risk of bias followed Cochrane guidance; certainty of evidence was appraised with GRADE.

**Results:**

Forty-one studies met inclusion. Pooled effects were statistically significant across domains: mental health µ(SMD)=0.76 (95% CrI 0.44–1.09), athletic performance µ(SMD)=0.88 (0.69–1.05), and cognitive performance µ(SMD)=0.81 (0.48–1.14).

**Conclusion:**

Biofeedback and neurofeedback benefit athletes across mental, athletic, and cognitive outcomes. Given heterogeneity and sample sizes, further rigorous trials are warranted to refine the estimates.

**Systematic review registration:**

PROSPERO registration CRD420251015094.

## Introduction

As an interdisciplinary technique that integrates physiology, psychology, and neuroscience, biofeedback training is increasingly recognized as an effective intervention for enhancing athletes’ mental health and performance (Kloudova, 2021). Research has demonstrated a significant positive correlation between optimal mental states and athletic performance, wherein effective regulation of emotional reactivity and anxiety enhances decision making and attentional control-key determinants of athletic success (Raglin, 2001; Rice et al., 2016). Therefore, an in-depth investigation into the application value of biofeedback in enhancing athletes’ mental health and performance represents a meaningful and timely direction in contemporary sports science research (Blumenstein et al., 2014).

Biofeedback can be defined as a technique that uses instruments to monitor an individual’s physiological activities in real time and provides feedback through visual or auditory means (Egner and Gruzelier, 2004; Giggins et al., 2013; Schwartz, 2010). Herbert Benson’s “relaxation response” theory suggests that by regulating the autonomic nervous system, biofeedback can effectively reduce the levels of stress hormones such as cortisol in the body, thereby alleviating the stress response (Lehrer et al., 2020). Furthermore, the core theoretical mechanism underlying biofeedback interventions lies in enhancing individuals’ ability to regulate autonomic nervous system activity particularly heart rate variability (HRV) through training. This process relies on the plasticity of vagal tone, whereby repeated practice improves the ability to identify and control the resonance frequency per week between heart rate and resonant frequency, thereby increasing parasympathetic activation (Lehrer et al., 2003; Pagani et al., 2009). Additionally, from the perspective of operant conditioning, biofeedback is a learning process where individuals achieve intentional control over their body states through awareness and regulation of physiological signals (Basmajian, 1983). This process reflects the fundamental view of the mind body interaction theory that psychological processes can have regulatory effects on physiological functions through the central nervous system (Blanchard and Young, 1979). Although these theories provide important theoretical support for the application of biofeedback training, current empirical research on it is still insufficient, and its true effectiveness remains controversial in multiple fields (Rydzik et al., 2023).

Recent research suggests that biofeedback training has a positive effect on improving athletes’ mental health (Saha et al., 2013), with statistically significant benefits observed in shooting athletes (Donghai et al., 2024a), and football players (Rusciano et al., 2017). Biofeedback training can effectively attenuate the stress response by modulating autonomic nervous system activity, thereby enhancing emotional regulation and cognitive function (Dehghani et al., 2023). Notably, the enhancement of physiological self-regulation through biofeedback not only contributes to improved self-efficacy but may also indirectly reduce anxiety (Goessl et al., 2017; Teufel et al., 2013). Taken together, these findings highlight that biofeedback training not only alleviates stress and anxiety through enhanced emotional and physiological self-regulation, but also builds a solid psychological foundation that may benefit athletic performance.

In addition to its positive impact on mental health, research also shows that biofeedback training has a direct promoting effect on athletic performance itself. It is also applicable to swimming (Bar-Eli, 2004), golf (Cheng et al., 2015), judo (Pronczuk et al., 2023), winter sports athletes (Toolis et al., 2024), and basketball players (Paul and Garg, 2012). Furthermore, improving the regulatory ability of the autonomic nervous system through biofeedback also helps enhance an individual’s cognitive performance (Pronczuk et al., 2023), such as attention control, working memory, and decision making ability. These cognitive factors play significant roles in complex and high speed competitive environments (Rusciano et al., 2017; Paul and Garg, 2012; Dana et al., 2019; Mikicin et al., 2015). Therefore, biofeedback training can not only enhance the self-regulation ability of physiology and emotion, but also support the improvement of cognitive efficiency, thereby comprehensively promoting the improvement of athletic performance (Brito et al., 2022; Tosti et al., 2024).

Although existing studies have to some extent verified the positive effects of biofeedback training on improving athletes’ mental health and performance, research in this field is still relatively scarce. Existing studies mostly focus on a specific sport or small sample experiments, lacking extensive coverage and in depth exploration across different sports (Paul and Garg, 2012; Bar-Eli et al., 2002; Wilson and Bird, 1981; Yılmaz et al., 2025). Most existing meta-analyses have primarily examined the effects of heart rate variability biofeedback on depression and general performance, often without specifically focusing on athlete populations or encompassing the full range of biofeedback modalities (Lehrer et al., 2020; Pizzoli et al., 2021). Consequently, there is a pressing need for more diverse and representative large-scale studies-particularly systematic empirical investigations across various sports-to comprehensively assess the applicability, developmental potential, and actual efficacy of biofeedback interventions in athletic settings (Lehrer et al., 2020).

Furthermore, although the majority of studies support the effectiveness of biofeedback training in improving mental health and enhancing both motor and cognitive performance, notable exceptions have also been reported. For instance, no significant differences in attentional performance were found between the experimental and control groups following neurofeedback training (Mirifar et al., 2019). Similarly, physical flexibility significantly improved across all three experimental groups after biofeedback training; however, the magnitude of improvement did not differ significantly between groups, suggesting that the specific type of training administered had no distinct effect on flexibility outcomes (Wilson and Bird, 1981). Moreover, the interaction between group type and training outcome was not statistically significant, indicating that group assignment did not moderate the training’s impact on flexibility gains (Wilson and Bird, 1981). Therefore, these differences suggest that the effects of biofeedback or neurofeedback training may not be universally applicable, and its benefits may depend on a variety of factors, including the specific sport participated in, the training program adopted, as well as the individual’s sports background and psychological characteristics, etc. (Tosti et al., 2024).

Building upon findings from prior studies, the present research incorporates a dose–response and moderation analysis to explore how variations in intervention dosage-defined by intervention time (weeks), frequency per week, and frequency per week-impact outcomes related to mental health, athletic performance, and cognitive performance. Using a Bayesian meta-analytic framework, this study systematically evaluates the overall effectiveness of biofeedback training among athletes, with the hypothesis-grounded in prior empirical evidence and dose-effect patterns-that such training yields significant positive effects across all three domains.

## Methods

This study was registered on the PROSPERO platform (registration number: CRD420251015094) and conducted in accordance with the PRISMA guidelines for systematic reviews and meta-analyses (Haddaway et al., 2022). During literature screening and data analysis, we used R (version 4.5.1), the Python-based ASReview tool, the Covidence platform, and GRADEprofiler to support screening and evaluation procedures.

### Inclusion criteria for screening

Literature screening followed the PICOS framework. Participants in the included studies were athletes of any age and health status. Only randomized controlled trials (RCTs) that used biofeedback or neurofeedback training as the intervention were eligible. The control group could include participants who received no psychological or physiological training, or those who underwent alternative skill training that did not involve biofeedback or neurofeedback.

Eligible studies were required to report outcome measures related to mental health, athletic performance, or cognitive performance. Publications in both English and Chinese were considered. Studies were excluded if they met any of the following criteria: (1) master’s or doctoral theses and conference abstracts, which were excluded to ensure methodological consistency and reliable data extraction; (2) non-original articles such as letters, editorials, or commentaries; (3) studies lacking extractable data; or (4) studies in which the experimental and control groups received different types of biofeedback or neurofeedback interventions.

### Information retrieval

A comprehensive search strategy was developed on February 17, 2025, using both Medical Subject Headings (MeSH) and free-text terms. Systematic searches were performed across eight databases: Ovid, Web of Science, Scopus, PubMed, Embase, PsycINFO, SPORTDiscus, and CNKI (China National Knowledge Infrastructure). The keywords and MeSH terms were discussed and finalized by four authors (XZ, ZC, SZ, and ZN). Detailed search strings for each database are provided in Supplementary File S1. Screening was conducted using the Covidence online platform and ASReview, a Python-based machine learning tool for literature prioritization (van de Schoot et al., 2021). A total of 5,527 studies were identified for further evaluation.

### Screening process

All titles and abstracts were first evaluated with ASReview, a machine learning based screening tool. ASReview predicts study relevance by training a classification model on labeled abstracts and continuously reprioritizes the remaining records according to their likelihood of inclusion (Holzinger, 2016; Van de Schoot et al., 2021; Wang et al., 2020). This approach markedly reduces manual workload by presenting the most likely relevant records first.

During this phase we applied the conservative SAFE rule, which stops screening only after 200 consecutive records have been judged irrelevant (Boetje and Van De Schoot, 2024). Full-text screening was then conducted independently by two reviewers (XZ and ZC) on the Covidence platform, as recommended in PRISMA guidelines. Eligible articles were recorded with the Extraction 1.0 form, and any disagreements were resolved by a third and fourth reviewer (SZ and NZ) (Boetje and Van De Schoot, 2024).

### Extract data information

For each study, the extracted features include the author, publication year, country, intervention, study design, biofeedback training or neurofeedback training, practice period, sample size, gender, athlete type, age, training years and outcome. The outcomes of mental health include anxiety, stress, anger, fatigue and depression. The results of athletic performance include golf performance, speed, swimming performance, balance, shooting performance, endurance, coordination, basketball performance, bowling performance, football performance, strength, running performance, rowing performance and flexibility. Cognitive performance results include attentional control, attentional focus, selective attention, task performance metrics and working-memory performance.

The data were extracted by two authors (XZ and ZC) respectively, and the differences were resolved through consultation with the third and fourth authors (SZ and ZN). The results are presented in the form of mean ± standard deviation (M ± SD). For the data that were not initially provided in M ± SD format, we used an online tool called Meta Analysis Accelerator for conversion (Abbas et al., 2024). Since none of the included studies reported correlation coefficients, a correlation coefficient of 0.5 was assumed for all analyses, following the recommendation of Follmann et al. (1992). When data were not provided in numerical form, we used GetData Graph Digitizer to extract the corresponding values from the figures (Digitizer, 2020).

### Risk bias assessment

The risk of bias in all included studies was independently evaluated according to the criteria in the Cochrane Handbook of Systematic Reviews of Interventions (Higgins et al., 2011). Two authors (XZ and ZC) evaluated the studies in randomized controlled trials (RCTs) through the Covidence tool in accordance with the Cochrane Risk of bias Assessment Criteria (ROB2), covering seven areas of bias: (1) Random sequence generation; (2) Allocation concealment (3) Blinding of participants and staff; (4) Blinding of outcome assessment; (5) incompleted data; (6) Selective Reporting (7) Other biases. The risk of bias is classified as low, unclear or high. All the assessment results were agreed upon through discussion and recorded in the Excel template. Subsequently, the data were input into the R software, and the bias risk summary graph was generated using the robvis package (McGuinness and Higgins, 2021).

### GRADE evidence grade evaluation

In the field of athletic performance research, this study adopts the GRADE method to systematically assess the quality of evidence from four core dimensions (Higgins et al., 2011). Firstly, the potential risk of bias in the included studies was assessed-specifically, the systematic errors that may arise during research design, implementation, or result reporting, which could compromise the validity of the conclusions. Secondly, heterogeneity across studies was evaluated using the I^2^ statistic to assess the degree of inconsistency in the results. Thirdly, the indirectness of the evidence was evaluated-specifically, whether the interventions, study populations, and outcome measures included in the reviewed studies directly addressed the core questions of this analysis. Finally, the imprecision of the effect estimates was assessed. The robustness and reliability of the conclusions were primarily evaluated based on the width of the confidence intervals around the effect sizes and the sample sizes of the included studies.

According to the GRADE standard, the quality of evidence is divided into four grades: “high,” “medium,” “low” and “very low,” reflecting the gradient level of evidence credibility from highly certain to highly uncertain.

### Data analysis

This study conducted a meta-analysis within the framework of Bayesian statistics to integrate prior information more comprehensively and quantify the uncertainty of the estimated values.

The overall meta-analysis was conducted using the bmeta package (version 4.5.1) in R software (Higgins et al., 2009). Firstly, with the help of the escalc() function of the meta forpackage, calculate the standardized mean difference (SMD) and its variance (vi) of each study, and then calculate the Standardized error (sei) and accuracy (1/vi). After establishing the data object, the bmeta() function is used to fit the random effects model (type = “ran”), and the model type is specified as the standard normal variance structure (model = “std.mv”). The total number of sampling iterations of MCMC is set at 50,000 times, and the burn in period is set to 20,000 iterations. The model output includes the estimation of the posterior mean of the effect size, the 95% Credible interval (CrI), and the heterogeneity parameter (tau). The heterogeneity level was supplemented and evaluated simultaneously by calculating the *I*^2^ value through the rma() function in the metafor package.

Subgroup analysis was accomplished using the bayesmeta (Röver, 2020) package (version 4.5.1). In addition, subgroup analyses were performed by athlete competitive level (elite vs. amateur) to examine potential differences in mental health, athletic performance, and cognitive performance outcomes. Furthermore, subgroup analyses were also performed according to blinding procedures (open-label vs. adequate blinding) to examine whether trial design characteristics influenced the observed effects.

Furthermore, Bayesian meta-regression models were fitted using the brms package to evaluate potential moderators of heterogeneity, including gender (percentage of female participants) and age (mean age of participants) (Bürkner, 2017; Carpenter et al., 2017). The model specification was yi | se(sei) ~ 1 + Moderator + (1 | Study), where yi denotes the standardized mean difference and see the corresponding standard error. Weakly informative priors (Normal(0,2) for intercepts, Cauchy(0,1) for random-effect SDs) were applied. Models were run with 8 chains, 4,000 iterations each (2000 warm-up), with adapt_delta set to 0.999 and max_treedepth set to 15. Model performance was assessed using Bayesian R^2^ with 95% credible intervals to quantify the proportion of variance explained.

The input includes the effect size (yi) and the standardized error (sei). The prior of the overall effect size is set as a normal distribution with a mean of 0 and a Standardized deviation of 5 (mu.prior.mean = 0,mu.prior.sd = 5) (Röver, 2020; Röver and Friede, 2023), and the heterogeneity parameter *τ* is set as a non-information uniform distribution (tau.prior = “uniform”). The bayesmeta() function was used to fit the model, and the prior and posterior images of the overall effect, heterogeneity, predicted distribution and their combined distribution were plotted to comprehensively present the uncertainty structure. All analyses were completed in R software (version 4.5.1). The 95% confidence interval (CrI) is interpreted as the probability that the true value of the parameter falls within this interval under the given data and model being 95%. The overall analysis process considers model transparency, estimation accuracy and bias test, providing solid statistical support for the research conclusion. The Bayesian meta-analysis used the Bmeta and Metafor escalc R packages to calculate effect size (SMD) and variance reciprocal in each study. The Bayesian approach is considered suitable for meta analyses including few studies, providing evidence for both null and alternative hypotheses, and offering complete information about credible parameter values and the probability of any given value (Higgins et al., 2009; Röver, 2020; Harrer et al., 2021; Kruschke and Liddell, 2018).

### Publication bias

To assess whether there was publication bias in the included studies, this study used the bmeta package and the bayesmeta package for the visualization analysis of funnel plots. Specifically, in the bmeta analysis, the funnel.plot() function is used to draw the funnel plot with the effect size as the horizontal axis and the Standardized error as the vertical axis, and the symmetry is visually checked to determine whether there is bias. In Bayesmeta analysis, the funnel.bayesmeta() function is used to further verify the existence of the small sample effect or potential bias within the Bayesian framework. Through visual examination of the symmetry of the funnel plot, if significant asymmetry is observed, it may suggest the existence of publication bias.

Furthermore, Egger’s regression test was performed, and both contour-enhanced funnel plots and sunset (power-enhanced) funnel plots were applied as complementary approaches. These methods enabled visualization of significance contours and study-level statistical power, thereby providing a more comprehensive assessment of potential publication bias (Duval and Tweedie, 2000). Specifically using the trimfill() function in the metafor package of the R language, combined with iterative operations, the number of missing studies is estimated, and the effect size is corrected accordingly, thereby enhancing the robustness of the research results and further improving the scientific nature of the conclusion.

## Results

The results of this study consist of six parts: literature screening process, summary of research characteristics, Risk of bias assessment, results of meta-analysis, publication bias test and GRADE evidence classification.

### Literature screening process

**Figure 1:**
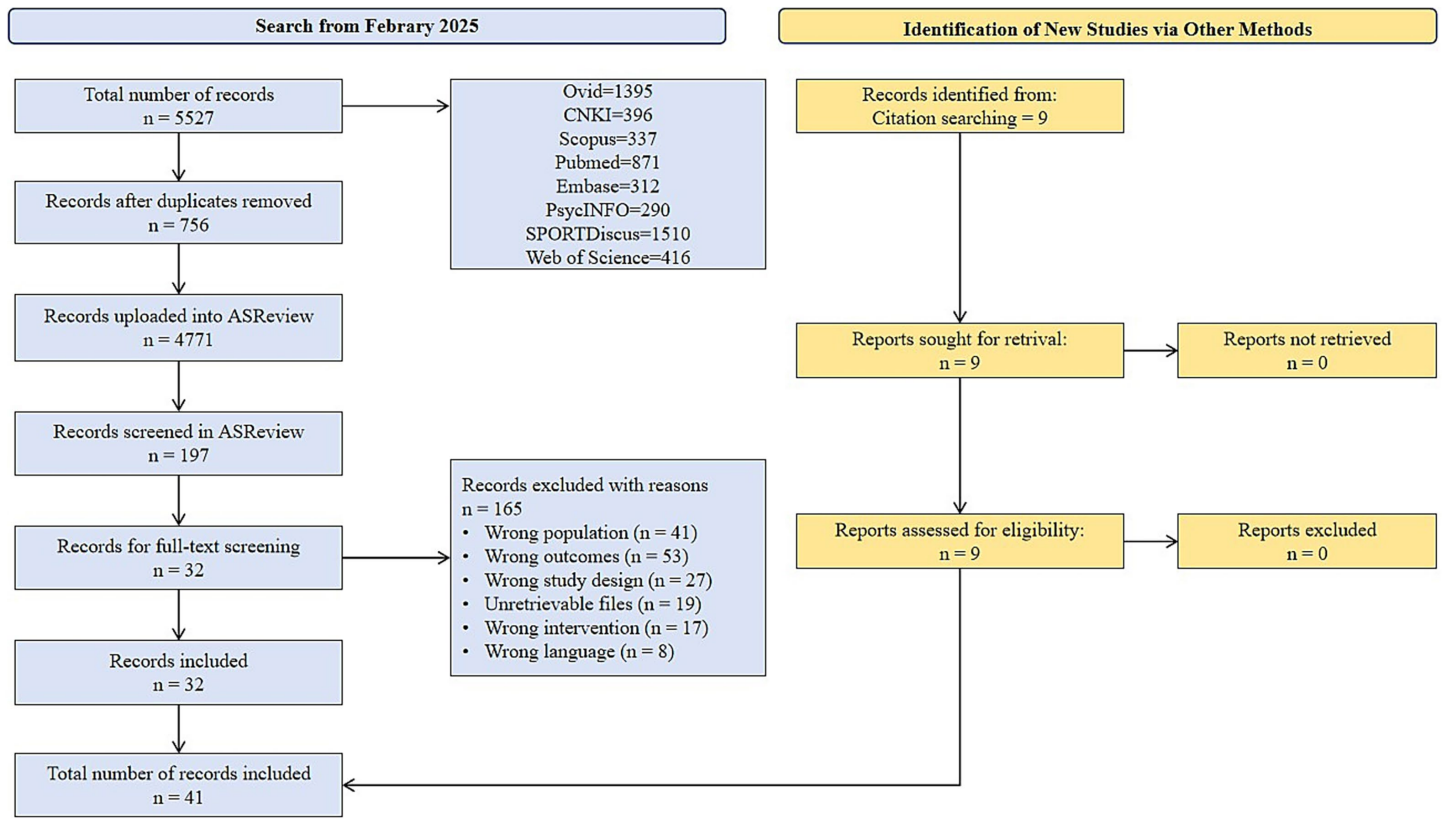
PRISMA flowchart of study selection.

Through systematic retrieval of eight databases (Ovid, CNKI, Scopus, Pubmed, Embase, PsycINFO, SPORTDiscus and Web of Science), 5,527 related literatures were initially obtained. EndNote X9 software was used to remove duplicates. 756 duplicate literatures were eliminated, and the remaining 4,771 entered the initial screening. The initial screening adopted ASReview for title and abstract screening. The machine learning model automatically evaluated 1,256 literatures, and finally 197 entered the full text screening stage. After reading the full text, 164 studies that did not meet the inclusion criteria were excluded, and 32 qualified studies were initially retained. To further ensure the completeness of the literature, an additional 9 related studies were included through citation retrospective supplementary search. Ultimately, 41 studies met the inclusion criteria of the meta analysis. The research screening process is detailed in Figure 1 (PRISMA flowchart).

### Characteristics included in the study

A total of 41 randomized controlled trials were included in this study, involving 1,230 athletes, including 905 males and 282 females. Additionally, gender data were missing for 43 participants (3.50% of the total sample), as reported in two of the included studies (Mikicin et al., 2015; Maszczyk et al., 2020). Among all the studies, 29 used biofeedback training as an intervention, and 12 used neurofeedback training. In terms of the geographical distribution of the studies, 22 studies were from Asia (accounting for 53.66%), 14 from Europe (accounting for 34.15%), 4 from North America (accounting for 9.76%), and 1 from Oceania (accounting for 2.44%). It should be noted that the age information of the subjects was not reported in 12 studies, accounting for 29.27% of the total included trials. For detailed characteristics of each study, please refer to the Supplementary Material S2.

### Risk of bias

**Figure 2:**
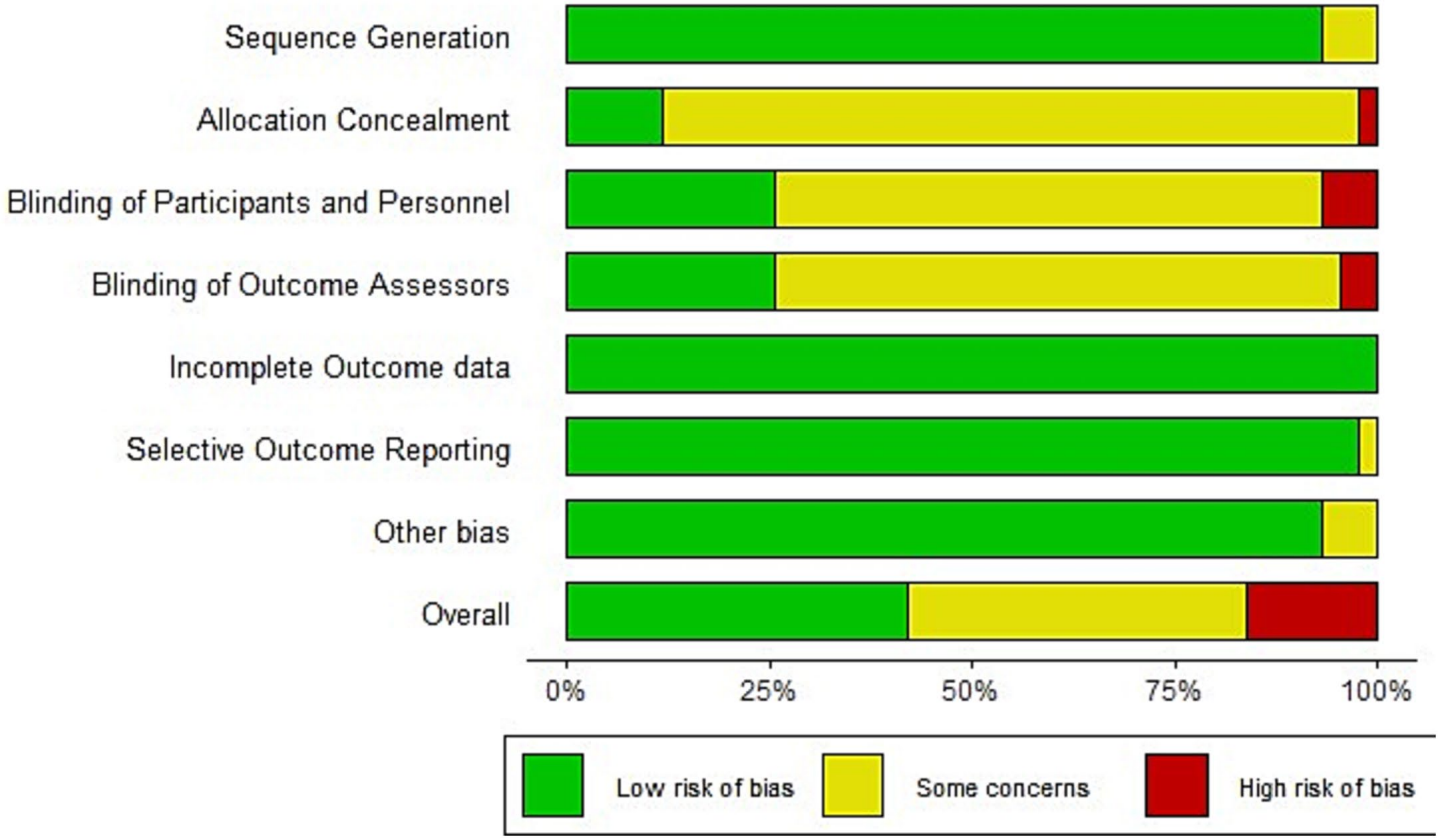
Risk of bias summary.

As shown in Figure 2, the risk of bias was evaluated across key methodological domains. In Sequence Generation, a small proportion (7.1%) of studies were rated as having some concerns due to insufficient detail about the randomization process; no study was considered high risk. In the field of allocation concealment, most studies were rated as high concern, with 85.4% rated as some concerns and 2.4% as high risk, mainly because concealment methods were not reported or were clearly inadequate. In the field of Blinding of Participants and Personnel, 65.9% of studies had some concerns and 4.9% were at high risk, often due to a lack of reported blinding in trials involving subjective outcomes. Similarly, in the field of Blinding of Outcome Assessors, 56.1% had some concerns and 4.9% were rated as high risk due to insufficient reporting on whether blinding was performed or absent when outcome evaluation could be influenced. For Incomplete Outcome Data and Selective Reporting, all studies were at low risk, reflecting proper data handling and transparent reporting. Overall, more than 45% of studies had at least some risk of bias, primarily due to missing or insufficient reporting on allocation and blinding procedures.

### Meta-analysis

The meta-analysis included 41 studies and focused on three primary outcomes: mental health, athletic performance, and cognitive performance in athletes. Specifically, 15 studies with 394 athletes examined the effects on mental health, 24 studies involving 2,320 athletes focused on athletic performance, and 11 studies with 348 athletes assessed cognitive performance. The results indicate that biofeedback and neurofeedback training have positive effects across all three domains, effectively improving athletes’ mental health, enhancing athletic performance, and strengthening cognitive performance.

#### Mental health

**Figure 3:**
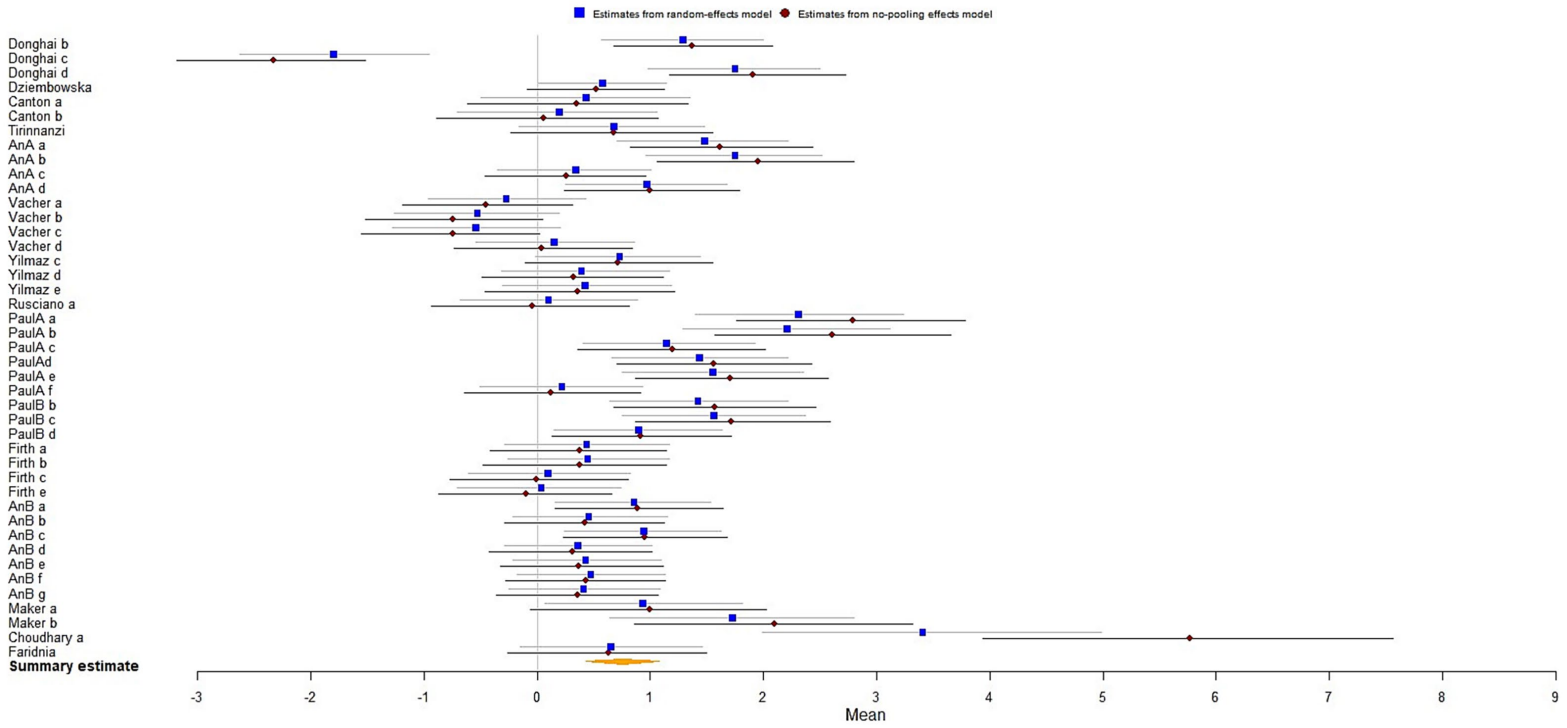
The forest plot in mental health.

The results analysis revealed that the biofeedback intervention had a significant moderate effect on improving the mental health of athletes [*μ*(SMD) = 0.76; 95% CrI: 0.44 to 1.09; *τ*(tau) = 0.99; Rhat = 1.001], indicating an overall positive impact on psychological well-being (Figure 3).

#### Athletic performance

**Figure 4:**
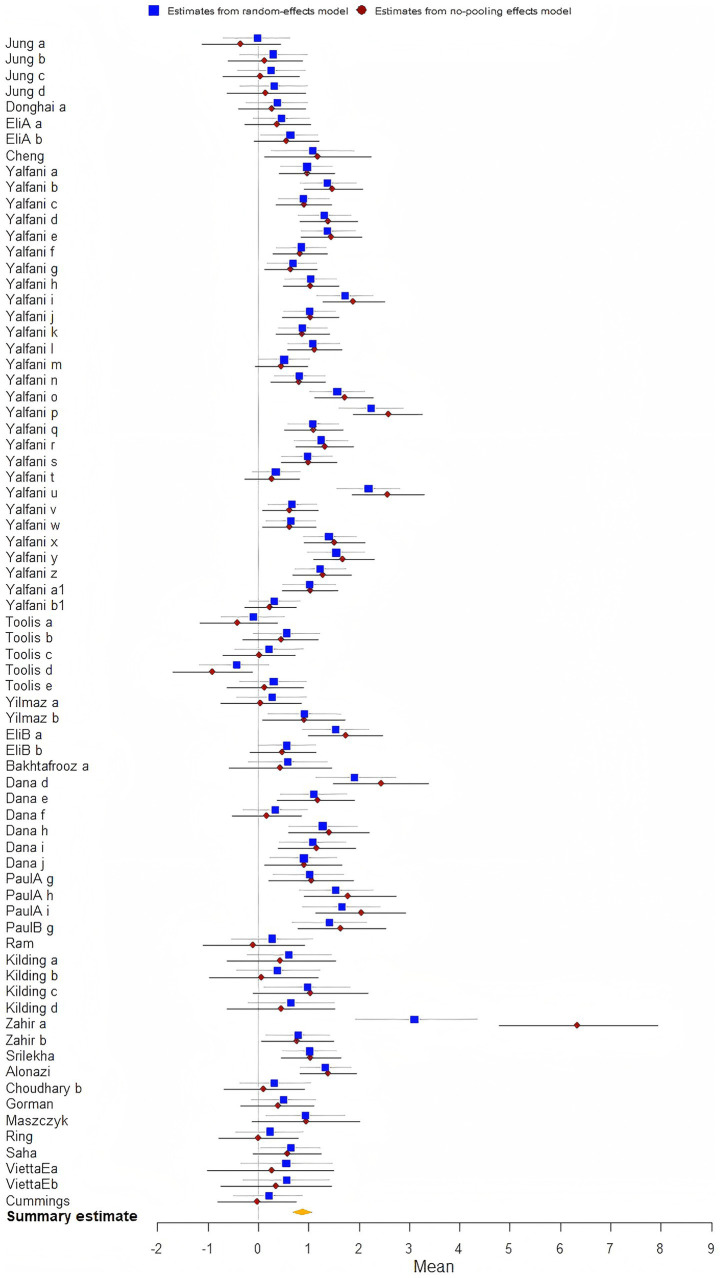
The forest plot in athletic performance.

The analysis demonstrated that the biofeedback intervention was found a statistical significance on enhancing athletic performance [*μ*(SMD) = 0.88; 95% CrI: 0.69 to 1.05; *τ*(tau) = 2.24; Rhat = 1.001], indicating strong evidence of improved Athletic performance among athletes (Figure 4).

#### Cognitive performance

**Figure 5:**
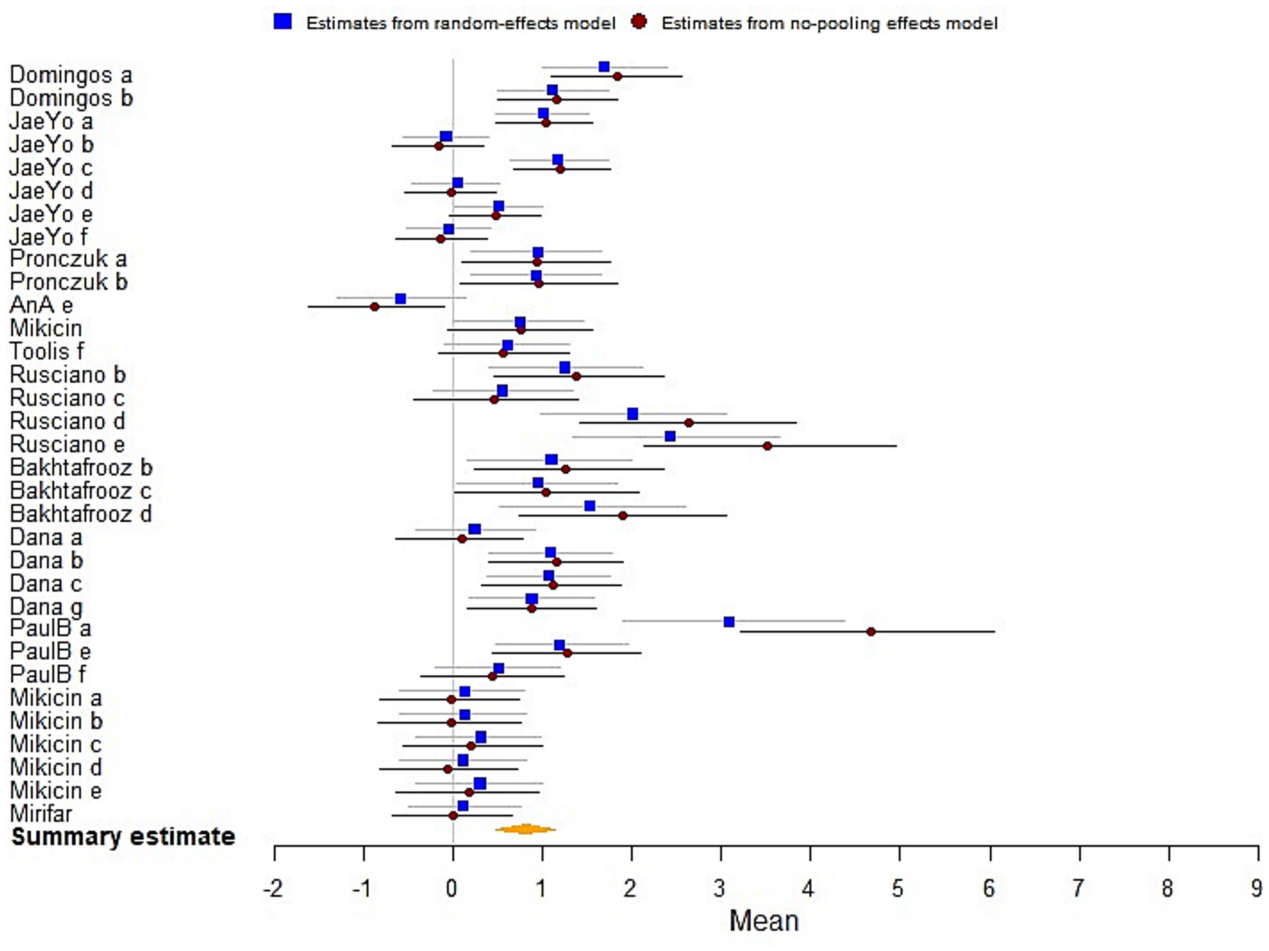
The forest plot in cognitive performance.

The results indicated statistically significant effects of biofeedback and neurofeedback training on cognitive performance [*μ*(SMD) = 0.81; 95% CrI: 0.48 to 1.14; *τ*(tau) = 1.42; Rhat = 1.001], demonstrating overall enhancements in cognitive performance (Figure 5).

### Subgroup analysis based on intervention type (biofeedback vs. neurofeedback)

#### Mental health (biofeedback and neurofeedback)

In terms of mental health, biofeedback interventions demonstrated a statistically significant effect [*μ*(SMD) = 0.76; 95% CrI: 0.42 to 1.10; *τ*(tau) = 1.01], indicating robust improvements in athletes’ psychological well-being. Neurofeedback, however, was represented by a single study only, yielding an effect estimate of [*μ*(SMD) = 0.61; 95% CrI: −0.28 to 1.51; *τ*(tau) not estimable]. The forest plots for each subgroup are provided in Supplementary Document S4.

#### Athletic performance (biofeedback and neurofeedback)

In terms of athletic performance, both biofeedback and neurofeedback interventions demonstrated statistically significant effects, with biofeedback yielding an effect size of [*μ*(SMD) = 0.85; 95% CrI: 0.46 to 1.26; *τ*(tau) = 0.89] and neurofeedback training [*μ*(SMD) = 0.89; 95% CrI: 0.68 to 1.09; *τ*(tau) = 0.64]. The forest plots for each subgroup are provided in Supplementary Document S4.

#### Cognitive performance (biofeedback and neurofeedback)

In terms of cognitive performance, both biofeedback [*μ*(SMD) = 0.97; 95% CrI: 0.40 to 1.54; *τ*(tau) = 1.44] and neurofeedback [*μ*(SMD) = 0.81; 95% CrI: 0.50 to 1.12; *τ*(tau) = 0.58] demonstrated Statistical significance. Forest plots for each subgroup are presented in Supplementary Document S4.

### Subgroup analysis based on specific psychological and performance outcomes

#### Biofeedback training

A total of 10 outcome domains were included in the subgroup analysis of biofeedback training. Statistically significant effects were observed in basketball performance [*μ*(SMD) = 1.59; 95% CrI: 0.61–2.59; *τ*(tau) = 0.66], pressure reduction [*μ*(SMD) = 0.72; 95% CrI: 0.35–1.10; *τ*(tau) = 0.89] and anxiety reduction [*μ*(SMD) = 1.02; 95% CrI: 0–2.04; *τ*(tau) = 1.56]. No statistical significance was found in other outcomes. Forest plots are presented in Supplementary Document S4.

#### Neurofeedback training

A total of six outcome domains were included in the neurofeedback analysis. Statistically significant effects were observed in both balance [*μ*(SMD) = 1.17; 95% CrI: 0.95 to 1.40; *τ*(tau) = 0.52] and attentional control [*μ*(SMD) = 0.68; 95% CrI: 0.03 to 1.39; *τ*(tau) = 0.69]. No statistically significant effects were found in the remaining athletic performance or cognitive performance domains. Forest plots are provided in Supplementary Document S4.

#### Subgroup analysis based on athlete competitive level

For mental health, elite athletes showed an estimated effect of *μ* = 0.86 (95% CrI: 0.46–1.25; *τ* = 1.11, 95% CrI: 0.78–1.46), while amateur athletes showed *μ* = 0.29 (95% CrI: −0.08–0.66; *τ* = 0.22, 95% CrI: 0.00–0.58).

For athletic performance, the effect for elite athletes was *μ* = 0.76 (95% CrI: 0.23–1.31; *τ* = 1.16, 95% CrI: 0.68–1.69), compared with *μ* = 0.94 (95% CrI: 0.76–1.12; *τ* = 0.56, 95% CrI: 0.40–0.72) for amateur athletes.

For cognitive performance, elite athletes showed *μ* = 1.01 (95% CrI: 0.47–1.58; *τ* = 1.15, 95% CrI: 0.69–1.66), whereas amateur athletes showed *μ* = 0.52 (95% CrI: 0.17–0.89; *τ* = 0.44, 95% CrI: 0.00–0.83).

Overall, these findings suggest some variation by competitive level, with elite athletes tending to show higher estimates in mental health and cognitive performance, and amateur athletes showing relatively higher estimates in athletic performance. Forest plots for these subgroups are provided in Supplementary Document S4.

#### Subgroup analysis based on blinding

Subgroup analyses based on blinding procedures revealed differential patterns across outcome domains. For mental health, open-label studies indicated a moderate effect with greater uncertainty [*μ*(SMD) = 0.63; 95% CrI: −0.16 to 1.43; *τ*(tau) = 1.58], whereas adequately blinded trials demonstrated a more precise and significant effect [*μ*(SMD) = 0.83; 95% CrI: 0.46 to 1.21; *τ*(tau) = 0.99].

For athletic performance, open-label studies showed negligible effects [*μ*(SMD) = 0.01; 95% CrI: −2.17 to 2.18; *τ*(tau) = 1.45], while adequately blinded trials yielded significant improvements [*μ*(SMD) = 0.86; 95% CrI: 0.48 to 1.25; *τ*(tau) = 0.97].

Regarding cognitive performance, adequately blinded studies showed statistically significant benefits [*μ*(SMD) = 0.86; 95% CrI: 0.44–1.29; *τ* = 1.00].

Overall, adequately blinded trials consistently yielded statistically significant effects, whereas open-label trials did not show significant results. Forest plots for these subgroups are provided in Supplementary Document S4.

### Subgroup analysis based on biofeedback dose

This section presents exploratory analyses of the relationship between different intervention dosages, categorized by intervention time (weeks), session length (minutes), and weekly frequency (sessions/week), and their effects on mental health, athletic performance, and cognitive performance outcomes. In the subgroup analyses across these outcome domains, interventions were consistently classified according to three dimensions to allow for systematic comparison. Based on intervention time (weeks), interventions were divided into three groups: less than 5 weeks, 6 to 10 weeks, and more than 10 weeks. In terms of session length, they were categorized as sessions lasting less than 20 min, 21 to 40 min, or 41 to 60 min. Regarding weekly frequency, interventions were classified as occurring 3 or fewer times per week, 4 to 5 times per week, or 6 to 7 times per week. These consistent classification criteria provided a comprehensive basis for evaluating the effectiveness of interventions across varying time frames and intensities. These analyses are exploratory and are not intended as dosage recommendations.

#### Mental health

Statistically significant effects were observed for duration <5 weeks [*μ*(SMD) = 0.76; 95% CrI: 0.48–1.04], weekly frequency 4–5 sessions/week [*μ*(SMD) = 1.06; 95% CrI: 0.73–1.41], and session length 21–40 min [*μ*(SMD) = 1.06; 95% CrI: 0.58–1.55]. Other bins did not consistently reach statistical significance. Detailed results and corresponding forest plots are presented in Supplementary Documents S4, S5.

#### Athletic performance

Statistically significant effects were observed for duration 6–10 weeks [*μ*(SMD) = 1.11; 95% CrI: 0.89–1.34], weekly frequency 4–5 sessions/week [*μ*(SMD) = 1.25; 95% CrI: 0.62–1.83], and session length 21–40 min [*μ*(SMD) = 0.81; 95% CrI: 0.25–1.37]. Other bins did not consistently reach statistical significance. Detailed results and corresponding forest plots are presented in Supplementary Documents S4, S5.

#### Cognitive performance

Statistically significant effects were observed for duration <5 weeks [*μ*(SMD) = 0.98; 95% CrI: 0.34–1.64], weekly frequency 3 sessions/week [*μ*(SMD) = 0.91; 95% CrI: 0.58–1.27], and session length <20 min [*μ*(SMD) = 0.53; 95% CrI: 0.05–1.02] and 41–60 min [*μ*(SMD) = 1.03; 95% CrI: 0.41–1.63]. Other bins did not consistently reach statistical significance. Detailed results and corresponding forest plots are presented in Supplementary Documents S4, S5.

### Moderator analyses

Using Bayesian meta-regressions, we examined demographic moderators. In these models, *R*^2^ denotes the proportion of between-study variance explained by the moderator, with higher values indicating that more of the heterogeneity across studies is accounted for.

For gender (percentage female), the estimated associations were: mental health, 0.48 (95% CrI: −0.91 to 1.92; *R*^2^ = 0.74, 95% CrI: 0.55–0.88); athletic performance, −1.08 (−1.87 to 0.31; *R*^2^ = 0.59, 0.42–0.76); cognitive performance, −0.97 (−2.47 to 0.56; *R*^2^ = 0.72, 0.46–0.89). None of these associations reached statistical significance, although negative trends were observed for athletic performance and cognitive performance.

For age (mean years), estimates were close to zero across domains-mental health, 0.00 (−0.08 to 0.09; *R*^2^ = 0.83, 0.65–0.94); athletic performance, 0.01 (−0.07 to 0.08; *R*^2^ = 0.61, 0.42–0.78); cognitive performance, 0.05 (−0.07 to 0.16; *R*^2^ = 0.74, 0.40–0.93). Moderator effect plots are provided in Supplementary Document S6.

### Publication bias

In this meta-analysis, we assessed potential publication bias across the three outcome domains (mental health, athletic performance, and cognitive performance).

Mental health: The funnel plot showed clear asymmetry, and Egger’s regression confirmed statistical significance (intercept = −2.48, 95% CI [−3.33, −1.62], *p* < 0.001). However, the trim-and-fill method did not impute additional studies, and the adjusted pooled effect size remained significant (SMD = 0.75, *p* < 0.001), suggesting that the main conclusions were not driven by publication bias.

Athletic performance: Egger’s test did not detect evidence of asymmetry (intercept = 0.89, 95% CI [0.53, 1.25], *p* = 0.98), and visual inspection of the funnel plot also suggested a symmetrical distribution of effect sizes, supporting the robustness of findings in this domain.

**Figure 6:**
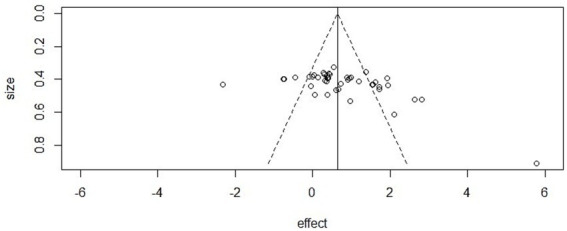
Funnel plot in mental health.

**Figure 7:**
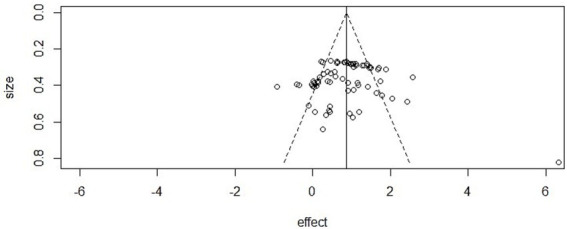
Funnel plot in athletic performance.

**Figure 8:**
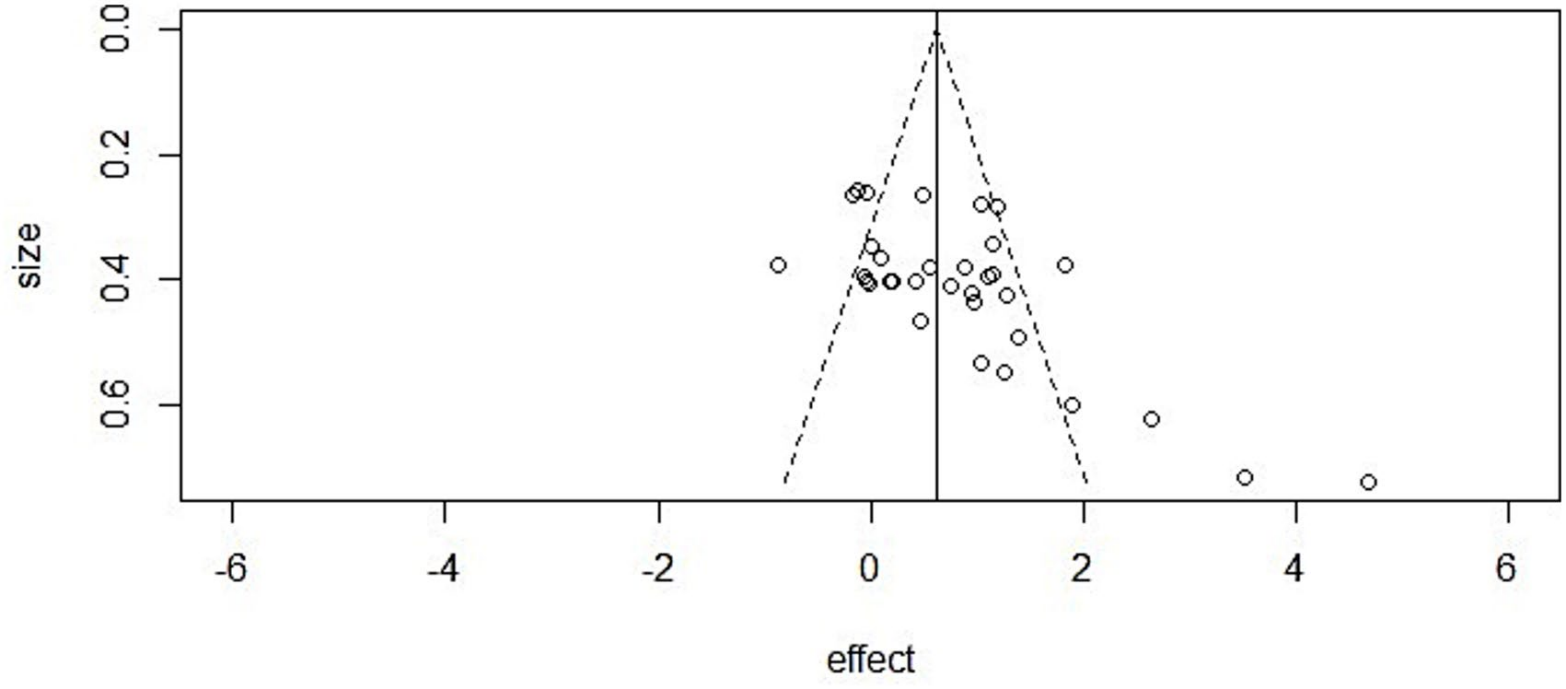
Funnel plot in cognitive performance.

Cognitive performance: The funnel plot appeared asymmetric, and Egger’s regression provided evidence of small-study effects (intercept = −1.15, 95% CI [−1.66, −0.65], *p* < 0.001). Nonetheless, the trim-and-fill method did not impute additional studies, and the adjusted effect remained statistically significant (*p* < 0.001) (Figures 6–8).

Beyond these conventional approaches, complementary analyses using contour-enhanced and sunset (power-enhanced) funnel plots also indicated potential small-study effects in mental health and cognitive performance, whereas results for athletic performance remained symmetrical. All extended funnel plot analyses are provided in Supplementary Figure S7.

### GRADE evidence grade evaluation

**Figure 9:**
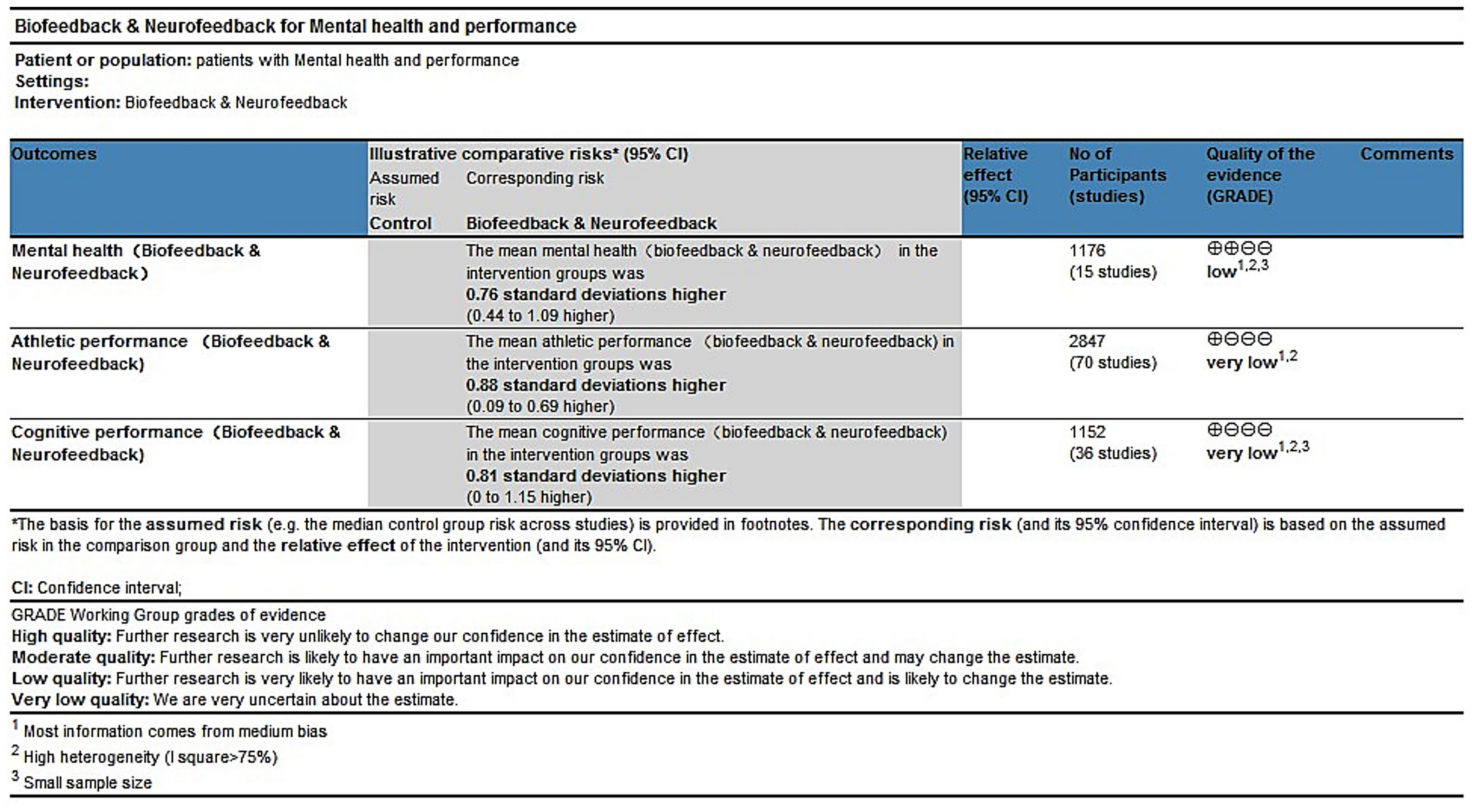
The GRADE summary in mental health, athletic performance and cognitive ability.

The GRADE assessment of the evidence regarding the effects of biofeedback and neurofeedback training on mental health, athletic performance, and cognitive performance indicated that the overall certainty of evidence was low to very low. Specifically, the quality of evidence for mental health outcomes was rated as low, while the evidence for athletic performance and cognitive performance was rated as very low. This downgrading was primarily due to moderate risk of bias in most studies, high heterogeneity, and insufficient sample sizes (Figure 9). The subgroup GRADE assessment charts are provided in Supplementary Document S8.

## Discussion

This study is the first to employ the Bayesian meta-analysis to explore the effects of biofeedback training on the mental health and performance of athletes. This systematic review and meta-analysis synthesize information about the impact of (1) biofeedback or neurofeedback on mental health, (2) athletic performance (3) and cognitive performance.

### Research findings

The results of this meta-analysis demonstrate that both biofeedback and neurofeedback training have statistically significant effects on athletes’ mental health, athletic performance, and cognitive performance. Subgroup analyses further elucidated the specific effectiveness of these interventions across different outcome domains.

Biofeedback training demonstrated statistically significant effects in improving mental health and enhancing athletic performance. These effects were most pronounced in improvements in anxiety reduction and basketball performance. Other outcome domains did not exhibit statistical significance under biofeedback interventions.

Neurofeedback training produced statistical significance in cognitive performance, particularly in enhancing attentional control. No other outcome domains reached statistical significance in the neurofeedback subgroup.

In the main analysis, the heterogeneity for cognitive performance was high (*τ* = 1.4). However, subgroup analysis further revealed that biofeedback contributed a higher heterogeneity (*τ* = 1.44) compared to neurofeedback (*τ* = 0.58), indicating that most of the heterogeneity in cognitive performance outcomes stemmed from biofeedback interventions.

Exploratory analyses suggested that some intervention dosage ranges may be associated with larger improvements. For mental health, effects appeared greater when interventions lasted 5 weeks or less, were delivered 4–5 times per week, and each session lasted 21–40 min. For athletic performance, relatively larger effects were observed with interventions lasting 6–10 weeks, conducted 4–5 times per week, with sessions of 41–60 min. For cognitive performance, improvements were observed in subgroups with interventions lasting 5 weeks or less, performed 3 times per week, and with sessions of either 20 min or less or 41–60 min. However, these patterns were not consistent across outcomes and the certainty of evidence was low; thus, they should be interpreted as exploratory findings and do not constitute dosage recommendations. Subgroup analyses based on athlete competitive level indicated that elite athletes benefited more in mental health and cognitive outcomes, while amateur athletes showed greater improvements in athletic performance. These findings suggest that the competitive background of athletes may moderate the effectiveness of biofeedback and neurofeedback interventions.

Moderator analyses revealed that gender did not significantly influence the effectiveness of biofeedback and neurofeedback interventions, while age accounted for a comparatively larger share of variance in athletic performance outcomes, although its effect was not statistically significant. According to the *R*^2^ values, part of the heterogeneity across studies may be explained by demographic factors such as age and gender. These findings suggest that demographic characteristics should be considered as potential contributors to heterogeneity in future research. In addition, the subgroup analysis by blinding suggested that adequately blinded trials tended to produce more consistent and reliable estimates with lower heterogeneity, underscoring the importance of rigorous blinding procedures in minimizing bias.

The observed domain-specific effects may be explained by underlying neurophysiological mechanisms. Biofeedback interventions, particularly heart rate variability and stress-regulation protocols, primarily target autonomic nervous system activity. By enhancing vagal tone and promoting parasympathetic dominance, biofeedback improves emotional regulation and stress recovery, which are especially relevant for psychological outcomes such as anxiety reduction and for sports like basketball where mental resilience and decision-making under pressure are crucial (Lehrer et al., 2003; Goessl et al., 2017; Paul and Garg, 2012). In contrast, neurofeedback protocols directly modulate cortical activity patterns, particularly within EEG frequency per bands associated with attentional control and sensorimotor integration. By reinforcing adaptive brain states-such as increasing SMR or frontal midline theta power while reducing maladaptive theta activity neurofeedback strengthens attentional focus and postural control, which may explain its stronger effects on cognitive outcomes and balance-related performance (Cheng et al., 2015; Dana et al., 2019; Gong et al., 2021; Yalfani et al., 2024; Enriquez-Geppert et al., 2017; Ros et al., 2020). Together, these mechanistic differences suggest that biofeedback and neurofeedback optimize complementary domains of athletic functioning, with biofeedback more closely aligned to stress resilience and psychological regulation, and neurofeedback more directly enhancing neural efficiency in attention and balance.

These findings suggest domain specific strengths for different types of biofeedback-based interventions.

### The impact of biofeedback training on mental health and performance

The results of this meta-analysis confirm that biofeedback training significantly enhances athletes’ mental health. Specifically, it helps reduce anxiety and alleviate stress. Biofeedback is not merely a tool for physiological regulation; it also plays a critical role in managing psychological stress. Existing studies support these findings, showing that biofeedback can effectively help athletes cope with anxiety and stress (Donghai et al., 2024a; Dziembowska, 2015; Tirinnanzi, 2022), By fostering greater interoceptive awareness and top down control over stress reactivity, Biofeedback helps athletes shift from reactive to proactive coping strategies by enhancing self-regulation and physiological awareness, thereby improving their mental toughness in high pressure environments (Donghai et al., 2024a). Notably, biofeedback induced improvements in autonomic regulation are closely linked to enhanced emotional regulation and cognitive control, both of which are critical in moderating anxiety responses during performance situations (Goessl et al., 2017; Teufel et al., 2013). These mechanisms offer a compelling explanation for the expanding role of biofeedback in sports psychology and athlete preparation.

Recent studies increasingly support the psychological and performance benefits of biofeedback training in athletic contexts (Yılmaz et al., 2025; Makaracı et al., 2023). This paragraph reviews key meta-analytic findings that validate its effectiveness, particularly in cognitively demanding sports (Tosti et al., 2024). Biofeedback training has demonstrated clear benefits across various sports, including basketball, football, swimming, and endurance disciplines (Saha et al., 2013; Paul and Garg, 2012). A recent systematic review (Pagaduan et al., 2022) highlight the positive effects of heart rate variability biofeedback on improving physiological regulation and performance outcomes in athletes particularly in basketball, shooting, and long distance running by enhancing respiratory mechanics, improving autonomic regulation, and reducing psychophysiological stress. Through the application of this technique, athletes can adjust their breathing frequency per week and enhance parasympathetic nervous system activity, optimizing both physiological responses and mental states. The importance of emotional regulation in improving performance is also underscored, especially under interventions that promote optimal heart rate variability and autonomic regulation. Therefore, the foundational concept proposed by Bar-Eli (2004) and later echoed by Yılmaz et al. (2025) which posits that biofeedback enhances athletic performance by simultaneously optimizing psychological and physiological states (Bar-Eli, 2004; Yılmaz et al., 2025; Pagaduan et al., 2022).

Biofeedback, particularly heart rate variability and biomechanical biofeedback, plays a critical role in enhancing both cognitive and athletic performance in athletes. This has been supported by multiple studies, including those by Gorman et al. (2021), Paul et al. (2012), and Saha et al. (2013), which collectively underscore the effectiveness of real-time physiological feedback in improving reaction time, concentration, and overall athletic execution. These findings highlight biofeedback as a valuable tool for athletes-not only for enhancing physical performance but also for sharpening cognitive functions under pressure. By training individuals to regulate both physiological responses and emotional states, biofeedback helps athletes achieve a state of optimal performance, particularly in high-stress, cognitively demanding sports contexts (Saha et al., 2013; Gorman et al., 2021; Paul et al., 2012).

In summary, current evidence highlights biofeedback as an effective intervention for improving both mental health and performance in athletes (Donghai et al., 2024b). By supporting physiological regulation and emotional control, it helps athletes manage stress, maintain focus, and perform more effectively under pressure (Goessl et al., 2017). These findings suggest that biofeedback holds strong potential for integration into athlete training and performance enhancement programs (Pagaduan et al., 2020).

### The impact of neurofeedback training on performance

Compared with the scarcity of research in the field of mental health, the promoting effect of neurofeedback training on athletic performance has received broader empirical support. Multiple studies have shown that neurofeedback training has significant effects in a series of sports that have high requirements for fine motor control, sensory and perceptual integration, and attention regulation, especially in golf, shooting, sprinting, static and dynamic balance events (Toolis et al., 2024; Dana et al., 2019; Yalfani et al., 2024; Bakhtafrooz et al., 2025; Chen et al., 2022). These sports usually require athletes to have a high degree of sensorimotor coordination, continuous concentration and moderate muscle relaxation to achieve precise and stable movement performance (Tosti et al., 2024). Neurofeedback training can improve these key neural mechanisms by regulating the characteristics of electroencephalogram (EEG) activities, thereby optimizing motor performance (Gong et al., 2021). Consistent with our subgroup findings, recent studies have demonstrated that neurofeedback training significantly improves balance-related athletic performance, particularly in sports requiring postural control and stability (Dana et al., 2019; Yalfani et al., 2024).

In athlete populations, neurofeedback training has shown notable efficacy in enhancing core cognitive performance that are closely tied to athletic performance, such as attention. Cognitive performance are crucial for optimizing decision making under pressure, maintaining performance consistency, and adapting rapidly to dynamic competitive environments (Tosti et al., 2024). Beyond motor performance, neurofeedback training has also been shown to significantly enhance attentional functioning, which is a key cognitive factor influencing athletic success. In sports settings, attention is critical for maintaining situational awareness, making rapid decisions, and sustaining consistent performance under pressure (Rydzik et al., 2023). Empirical studies have found that neurofeedback training protocols targeting the modulation of specific EEG bands such as enhancing sensorimotor rhythm (SMR) and beta activity while suppressing theta waves can lead to measurable improvements in various aspects of attention, including alertness, orienting efficiency, and conflict monitoring (Dana et al., 2019; Mikicin et al., 2015; Bakhtafrooz et al., 2025). These improvements reflect neurofeedback training capacity to promote adaptive cortical arousal states, reduce cognitive interference, and strengthen athletes’ ability to maintain task relevant focus (Cheng et al., 2024). Collectively, this evidence suggests that neurofeedback training serves as a dual function intervention, meanwhile, optimize the performance of the athlete population (Tosti et al., 2024).

Therefore, neurofeedback training may enhance performance through a dual mechanism: by optimizing sensorimotor control essential for precise physical execution, and by reinforcing attentional regulation that supports consistency and adaptability in high-pressure environments (Cheng et al., 2024). This convergence of motor and cognitive improvements reinforces neurofeedback’s unique value in sports contexts where both physical precision and mental toughness are critical for success (Tosti et al., 2024).

### Dose reporting

While the majority of studies in our meta-analysis support the effectiveness of biofeedback and neurofeedback in improving psychological self-regulation and athletic performance, significant variability was observed across intervention duration (weeks), weekly frequency (sessions/week), and session length (minutes). This variation underscores the need for a more standardized approach to biofeedback intervention protocols and reporting. These subgroup findings are exploratory and do not constitute dosage recommendations.

Furthermore, Onagawa et al. (2023) highlighted the importance of intervention duration, noting that programs extending beyond 10 weeks may yield reduced incremental benefits (Onagawa et al., 2023) Our findings are consistent with this possibility in some subgroups; however, the evidence is limited. These ranges need to be tested in preregistered randomized trials that systematically manipulate duration, session length, and weekly frequency.

Taken together, the results suggest that intervention duration, weekly frequency, and session length may influence the effectiveness of biofeedback. To ensure consistent evaluation and replication, future studies should adopt standardized dose reporting [e.g., the Consensus on the Reporting and Experimental Design of Neurofeedback studies, CRED-nf checklist (Ros et al., 2020; Onagawa et al., 2023; Yu et al., 2025; Schulz et al., 2010; Hassan et al., 2022), and preregister trial protocols, specifying planned dose ranges and analyses to enable robust assessment of long-term impact].

### Strengths and limitations

This study adopted the Bayesian meta-analysis for the first time to systematically evaluate the effects of biofeedback and neurofeedback training on athletes’ mental health, athletic performance and cognitive performance. Compared with the traditional Frequentist statistical methods, Bayesian analysis can provide more robust effect estimation and allow for the direct calculation of the probability distribution of the intervention effect, thereby enhancing the interpretability of the research conclusion (Van De Schoot et al., 2021). To ensure high data quality and reliability of the results, a rigorous literature screening process was implemented using the PICOS framework, comprehensive subgroup analyses, and a transparent inclusion protocol. In particular, this study employed ASReview, a machine learning–assisted systematic review tool, to improve efficiency and objectivity in the screening process. ASReview significantly reduces reviewer bias and enhances reproducibility by prioritizing relevant studies based on active learning algorithms, making the screening both faster and more evidence driven compared to traditional manual methods.

Although biofeedback training demonstrated statistically significant improvements in anxiety reduction, basketball performance, and pressure management, the benefits did not generalize across all measured domains. Among the 10 outcome indicators analyzed under biofeedback, only three reached statistical significance, suggesting domain specificity in its effectiveness. Additionally, this study did not systematically compare the differential effects of various types of biofeedback, such as heart rate variability feedback and electromyography feedback, limiting our ability to identify which modalities are most effective.

Interpretation of the findings is complicated by high between-study heterogeneity across all three primary outcomes (*τ* = 0.99 for mental health; 2.24 for athletic performance; 1.42 for cognitive performance). Subgroup analyses by intervention type, dose, blinding, and competitive level, as well as moderator analyses (age, gender), reduced-but did not eliminate this variability. Inconsistencies in blinding procedures and variability in study quality may also have contributed to the instability of the results, underscoring the need for future high-quality, rigorously blinded trials. Trim-and-fill adjustments did not materially alter the pooled estimates, whereas contour- and power-enhanced funnel plots indicated small study effects in the mental health and cognitive domains; findings for these outcomes should therefore be interpreted with caution. Given the diversity of study designs, intervention protocols, participant characteristics, and outcome measures across the included RCTs, residual heterogeneity remained despite these analytic controls. In addition, a few studies reported extremely small variances, which disproportionately increased their statistical weights and led to unstable estimates of between-study heterogeneity (τ) in some subgroups. These cases should therefore be interpreted with caution (Pronczuk et al., 2023; Dziembowska, 2015; Bakhtafrooz et al., 2025). Future studies should adopt more consistent protocols and standardized outcome definitions to improve comparability and precision.

The number of studies using neurofeedback training as an intervention was relatively limited, and their sample sizes were generally small. Among the outcome domains analyzed, only attentional control and balance reached statistical significance, whereas other domains did not demonstrate consistent effects. In the domain of mental health, only one study on neurofeedback training (Faridnia et al., 2012) was available, which limited the reliability of subgroup findings. Although an effect size could be estimated, the scarcity of evidence precludes firm conclusions, thereby restricting the generalizability of neurofeedback’s effects on mental health. Future research with larger samples and more rigorous designs is necessary to better understand the effectiveness of neurofeedback across various domains.

While the dose–response analysis indicated statistically significant effects for certain categories of intervention time (weeks), frequency per week, and time per session, most other subgroups did not reach significance, reflecting variability in the impact of intervention doses. In addition, only six trials included any form of follow up, and none extended beyond 6 months, which limited the ability to evaluate the long-term sustainability of intervention effects. Furthermore, the certainty of evidence assessed by the GRADE framework was rated as low for mental health outcomes and very low for both athletic and cognitive performance outcomes. These ratings underscore that, despite statistically significant pooled effects, the strength of evidence remains limited due to factors such as risk of bias, heterogeneity, small sample sizes, and potential publication bias. Consequently, the findings should be interpreted with caution and future high-quality trials are warranted to strengthen the evidence base.

Future research should address several limitations identified in this study. First, more detailed comparisons of different biofeedback modalities (e.g., heart rate variability vs. electromyography) are needed to determine which are most effective for various outcomes. Larger sample sizes and more neurofeedback studies, especially in mental health, would increase the reliability of findings. Standardizing outcome measures and reducing study heterogeneity through subgroup analyses could improve consistency across studies. Long-term follow-ups should be incorporated to assess the sustainability of intervention effects, as most current studies only track short-term outcomes. Additionally, more robust dose–response analyses are needed to identify the optimal intervention time (weeks), frequency per week, and frequency per week of interventions. Addressing these gaps will help refine the understanding of biofeedback and neurofeedback training’s effects on athletes. Future studies should also adopt standardized methodological frameworks such as the BEST toolbox and the CONSORT guidelines, in addition to the CRED-nf checklist, to further improve methodological consistency, reduce heterogeneity, and enhance the reproducibility of findings in this field (Ros et al., 2020; Schulz et al., 2010; Hassan et al., 2022).

## Conclusion

This meta-analysis provides evidence that biofeedback training has statistically significant effects on improving athletes’ mental health, particularly through reductions in anxiety and improved pressure management. Significant improvements were also found in athletic performance, especially in basketball. Meanwhile, neurofeedback training demonstrated statistically significant effects primarily in the domain of cognitive performance, with notable gains in attentional control and balance.

Exploratory subgroup analyses suggested that intervention dosage may influence the observed effects. Some dose ranges appeared to show larger improvements (e.g., mental health: intervention time <5 weeks, frequency 4–5 times per week, time per session 21–40 min; athletic performance: intervention time 6–10 weeks, frequency 4–5 times per week, time per session 41–60 min; cognitive performance: intervention time <5 weeks, frequency 3 times per week, time per session either <20 min or 41–60 min). These findings provide preliminary evidence of potential dosage effects, particularly for biofeedback on mental health. Future studies are needed to generate more robust and confirmatory evidence.

These findings support the use of biofeedback and neurofeedback as targeted interventions to improve specific psychological and performance outcomes in athletes. Further research is needed to explore the mental health effects of neurofeedback, standardize intervention protocols, and evaluate long-term outcomes. This study provides a comprehensive evaluation of the effects of biofeedback training on athletes’ mental health and performance through systematic review and Bayesian meta-analysis, offering practical guidance and significant theoretical and practical value.

## Data Availability

The original contributions presented in the study are included in the article/Supplementary material, further inquiries can be directed to the corresponding author.
