# Supplementary material for: The effects of biofeedback training on athletes’ mental health and performance: a systematic review and Bayesian meta-analysis
Source: Front Psychol. 2025 Oct 21;16:1662868. doi: 10.3389/fpsyg.2025.1662868 (PMC12583207; doi:10.3389/fpsyg.2025.1662868)
Supplement: Supplementary file 1 [file Data_Sheet_1.ZIP › Supplementary file S1 Search strategy.pdf]

Supplementary File S1 : Search Strategy

1.Pudmed

1. Search: ((((((((((((((((((((((((Biofeedback, Psychology) OR (Biofeedbacks, Psychology)) OR (Psychology Biofeedback)) OR (Psychology Biofeedbacks)) OR (Biofeedback)) OR (Biofeedbacks)) OR (Biofeedback (Psychology))) OR (Biofeedbacks (Psychology))) OR (Feedback, Psychophysiologic)) OR (Feedback, Psychophysiological)) OR (Psychophysiologic Feedback)) OR (False Physiological Feedback)) OR (False Physiological Feedbacks)) OR (Feedback, False Physiological)) OR (Feedbacks, False Physiological)) OR (Physiological Feedback, False)) OR (Physiological Feedbacks, False)) OR (Bogus Physiological Feedback)) OR (Bogus Physiological Feedbacks)) OR (Feedback, Bogus Physiological)) OR (Feedbacks, Bogus Physiological)) OR (Physiological Feedback, Bogus)) OR (Physiological Feedbacks, Bogus)) OR (Myofeedback)) OR (Myofeedbacks)

2. Search: ((((((((((((((((((((((((((Neurofeedback) OR (Neurofeedbacks)) OR (Brainwave Biofeedback)) OR (Biofeedback, Brainwave)) OR (Biofeedbacks, Brainwave)) OR (Brainwave Biofeedbacks)) OR (Alpha Biofeedback)) OR (Alpha Biofeedbacks)) OR (Biofeedback, Alpha)) OR (Biofeedbacks, Alpha)) OR (Alpha Feedback)) OR (Alpha Feedbacks)) OR (Feedback, Alpha)) OR (Feedbacks, Alpha)) OR (Brainwave Feedback)) OR (Brainwave Feedbacks)) OR (Feedback, Brainwave)) OR (Feedbacks, Brainwave)) OR (EEG Feedback)) OR (EEG Feedbacks)) OR (Feedback, EEG)) OR (Feedbacks, EEG)) OR (Electroencephalography Biofeedback)) OR (Biofeedback, Electroencephalography)) OR (Biofeedbacks, Electroencephalography)) OR (Electroencephalography Biofeedbacks)) OR (Electromyography Feedback)

3. Search: ((((((((Athletic Performance) OR (Athletic Performances)) OR (Performance, Athletic)) OR (Performances, Athletic)) OR (Sports Performance)) OR (Performance, Sports)) OR (Performances, Sports)) OR (Sports Performances)) OR ((((((((((((((((((((((Psychomotor Performance) OR (Performance, Psychomotor)) OR (Performances, Psychomotor)) OR (Psychomotor Performances)) OR (Perceptual Motor Performance)) OR (Motor Performance, Perceptual)) OR (Motor Performances, Perceptual)) OR (Perceptual Motor Performances)) OR (Performance, Perceptual Motor)) OR (Performances, Perceptual Motor)) OR (Sensory Motor Performance)) OR (Motor Performance, Sensory)) OR (Motor Performances, Sensory)) OR (Performance, Sensory Motor)) OR (Performances, Sensory Motor)) OR (Sensory Motor Performances)) OR (Visual Motor Coordination)) OR (Coordinations, Visual Motor)) OR (Coordination, Visual Motor)) OR (Motor Coordinations, Visual)) OR (Motor Coordination, Visual)) OR (Visual Motor Coordinations))

4. Search: ((((((((((((((Mental Health) OR (Health, Mental)) OR (Mental Hygiene)) OR (Hygiene, Mental)) OR (anxiety)) OR (depression)) OR (happ)) OR (mental toughness)) OR (Cognitive abilities)) OR (HRV)) OR(psychological well-being)) OR (mental well-being)) OR (cognitive abililty)) OR (emotion)) OR (mood)

5. Search: (((((((((((((Cognitive performance) OR (Cognitive function)) OR (Cognitive ability)) OR (Cognitive processing)) OR (Cognitive assessment)) OR (Cognitive load)) OR (Cognitive impairment)) OR (Executive function)) OR (Memory performance)) OR (Attention)) OR (Neurocognitive performance)) OR (Mental performance)) OR (Cognitive enhancement)) OR

(Cognitive training)
6. Search: ((((((((((((Athletes) OR (Athlete)) OR (Professional Athletes)) OR (Athlete, Professional)) OR (Athletes, Professional)) OR (Professional Athlete)) OR (Elite Athletes)) OR (Athlete, Elite)) OR (Athletes, Elite)) OR (Elite Athlete)) OR (College Athletes)) OR (Athlete, College)) OR (Athletes, College)) OR (College Athlete)
1 OR 2
3 OR 4 OR 5
7 AND 8 AND 9

2.OVID

1.("Biofeedback Psychology" or "Biofeedbacks Psychology" or "Psychology Biofeedback" or "Psychology Biofeedbacks" or Biofeedback or Biofeedbacks or "Biofeedback Psychology" or "Biofeedbacks Psychology" or "Feedback Psychophysiologic" or "Feedback Psychophysiological" or "Psychophysiologic Feedback" or "False Physiological Feedback" or "False Physiological Feedbacks" or "Feedback False Physiological" or "Feedbacks False Physiological" or "Physiological Feedback False" or "Physiological Feedbacks False" or "Bogus Physiological Feedback" or "Bogus Physiological Feedbacks" or "Feedback Bogus Physiological" or "Feedbacks Bogus Physiological" or "Physiological Feedback Bogus" or "Physiological Feedbacks Bogus" or Myofeedback or Myofeedbacks).af.

2.(Neurofeedback or Neurofeedbacks or "Brainwave Biofeedback" or "Biofeedback Brainwave" or "Biofeedbacks Brainwave" or "Brainwave Biofeedbacks" or "Alpha Biofeedback" or "Alpha Biofeedbacks" or "Biofeedback Alpha" or "Biofeedbacks Alpha" or "Alpha Feedback" or "Alpha Feedbacks" or "Feedback Alpha" or "Feedbacks Alpha" or "Brainwave Feedback" or "Brainwave Feedbacks" or "Feedback Brainwave" or "Feedbacks Brainwave" or "EEG Feedback" or "EEG Feedbacks" or "Feedback EEG" or "Feedbacks EEG" or "Electroencephalography Biofeedback" or "Biofeedback Electroencephalography" or "Biofeedbacks Electroencephalography" or "Electroencephalography Biofeedbacks" or "Electromyography Feedback").af.

3.("Athletic Performance" or "Athletic Performances" or "Performance Athletic" or "Performances Athletic" or "Sports Performance" or "Performance Sports" or "Performances Sports" or "Sports Performances" or "Psychomotor Performance" or "Performance Psychomotor" or "Performances Psychomotor" or "Psychomotor Performances" or "Perceptual Motor Performance" or "Motor Performance Perceptual" or "Motor Performances Perceptual" or "Perceptual Motor Performances" or "Performance Perceptual Motor" or "Performances Perceptual Motor" or "Sensory Motor Performance" or "Motor Performance Sensory" or "Motor Performances Sensory" or "Performance Sensory Motor" or "Performances Sensory Motor" or "Sensory Motor Performances" or "Visual Motor Coordination" or "Coordinations Visual Motor" or "Coordination Visual Motor" or "Motor Coordinations Visual" or "Motor Coordination Visual" or "Visual Motor Coordinations").af.

4.("Mental Health" or "Health Mental" or "Mental Hygiene" or "Hygiene Mental" or Anxiety or depression or happ or Stress or "mental toughness" or "Cognitive abilities" or "psychological well being" or HRV or "mental well being" or "cognitive abililty" or Emotion or Mood).af.

5.("Cognitive Ability" or "Cognitive Function" or "Cognitive Assessment" or "Cognitive

Impairment" or "Cognitive Training" or "Working Memory" or "Attention Control" or "Decision Making Ability" or "Information Processing Speed" or "Learning and Memory" or "Executive Function" or "Cognitive Load" or "Psychomotor Reaction Time" or "Brain Plasticity" or "Cognitive Decline" or Neurocognition or "Cognitive Aging" or "Emotional Impact on Cognitive Performance" or "Cognitive Performance Assessment Tools" or "Neuroscience and Cognition").af.

6.(Athletes or Athlete or "Professional Athletes" or "Athlete Professional" or "Athletes Professional" or "Professional Athlete" or "Elite Athletes" or "Athlete Elite" or "Athletes Elite" or "Elite Athlete" or "College Athletes" or "Athlete College" or "Athletes College" or "College Athlete").af.

7. 1 or 2

8. 3 or 4 or 5

9. 6 and 7 and 8

3.Psycinfo

1. (biofeedback* OR "biofeedback therapy" OR "biofeedback intervention") OR TX (biofeedback* OR "biofeedback therapy" OR "biofeedback intervention") OR (neurofeedback* OR "neurofeedback therapy") OR TX (neurofeedback* OR)

2. TX (exerci* OR exercise* OR "athletic performance" OR athletic* OR sports* OR sport* OR "sports performance" OR strength* OR "muscle strength" OR "endurance performance" OR endurance* OR agility* OR "cognitive ability" OR "response time" OR aerobic* OR anaerobic*)

3. TX ("mental health" OR happ* OR anxiety* OR depression* OR stress* OR "mental toughness OR motivation* OR happiness* OR "psychological well-being" OR "mental well-being" OR flow* OR pain* OR panic* OR anger* OR vividness* OR mood* OR emotion* OR concentration* OR HRV* OR cortisol*)

4. TX ("Cognitive Ability" OR "Cognitive Function" OR "Cognitive Assessment" OR "Cognitive Impairment" OR "Cognitive Training" OR "Working Memory" OR "Attention Control" OR "decision-making ability" OR "information processing speed" OR "learning and memory" OR "executive function" OR "cognitive load")

5. S2 OR S3 OR S4

6.TX (athletes* OR players* OR "para-athletes" OR cyclists* OR runners* OR golfers* OR wrestlers* OR "rugby players" OR "tennis players" OR boxers* OR "disabled athletes" OR shooters*)

7.S1 AND S5 AND S6

4.Scopus

1.(TITLE-ABS-KEY ("biofeedback, psychology") OR TITLE-ABS-KEY ("biofeedbacks, psychology") OR TITLE-ABS-KEY ("psychology biofeedback") OR TITLE-ABS-KEY ("psychology biofeedbacks") OR TITLE-ABS-KEY (biofeedback) OR TITLE-ABS-KEY (biofeedbacks) OR TITLE-ABS-KEY ("biofeedback psychology") OR TITLE-ABS-KEY ("biofeedbacks psychology") OR TITLE-ABS-KEY ("feedback, psychophysiologic") OR

TITLE-ABS-KEY ("feedback, psychophysiological") OR TITLE-ABS-KEY ("psychophysiologic feedback") OR TITLE-ABS-KEY ("false physiological feedback") OR TITLE-ABS-KEY ("false physiological feedbacks") OR TITLE-ABS-KEY ("feedback, false physiological") OR TITLE-ABS-KEY ("feedbacks, false physiological") OR TITLE-ABS-KEY ("physiological feedback, false") OR TITLE-ABS-KEY ("physiological feedbacks, false") OR TITLE-ABS-KEY ("bogus physiological feedback") OR TITLE-ABS-KEY ("bogus physiological feedbacks") OR TITLE-ABS-KEY ("feedback,bogus physiological") OR TITLE-ABS-KEY ("feedbacks,bogus physiological") OR TITLE-ABS-KEY ("physiological feedback,bogus") OR TITLE-ABS-KEY ("physiological feedbacks, bogus") OR TITLE-ABS-KEY (myofeedback) OR TITLE-ABS-KEY (myofeedbacks))

2. (TITLE-ABS-KEY (neurofeedback) OR TITLE-ABS-KEY (neurofeedbacks) OR TITLE-ABS-KEY ("Brainwave Biofeedback") OR TITLE-ABS-KEY ("Biofeedback, Brainwave") OR TITLE-ABS-KEY ("Biofeedbacks, Brainwave") OR TITLE-ABS-KEY ("Brainwave Biofeedbacks") OR TITLE-ABS-KEY ("Alpha Biofeedback") OR TITLE-ABS-KEY ("Alpha Biofeedbacks") OR TITLE-ABS-KEY ("Biofeedback, Alpha") OR TITLE-ABS-KEY ("Biofeedbacks, Alpha") OR TITLE-ABS-KEY ("Alpha Feedback") OR TITLE-ABS-KEY ("Alpha Feedbacks") OR TITLE-ABS-KEY ("Feedback, Alpha") OR TITLE-ABS-KEY ("Feedbacks, Alpha") OR TITLE-ABS-KEY ("Brainwave Feedback") OR TITLE-ABS-KEY ("Brainwave Feedbacks") OR TITLE-ABS-KEY ("Feedback, Brainwave") OR TITLE-ABS-KEY ("Feedbacks, Brainwave") OR TITLE-ABS-KEY ("EEG Feedback") OR TITLE-ABS-KEY ("EEG Feedbacks") OR TITLE-ABS-KEY ("Feedback, EEG") OR TITLE-ABS-KEY ("Feedbacks, EEG") OR TITLE-ABS-KEY ("Electroencephalography Biofeedback") OR TITLE-ABS-KEY ("Biofeedback, Electroencephalography") OR TITLE-ABS-KEY ("Biofeedbacks, Electroencephalography"))

3. (TITLE-ABS-KEY ("Athletic Performance") OR TITLE-ABS-KEY ("Athletic Performances") OR TITLE-ABS-KEY ("Performance, Athletic") OR TITLE-ABS-KEY ("Performances, Athletic") OR TITLE-ABS-KEY ("Sports Performance") OR TITLE-ABS-KEY ("Performance, Sports") OR TITLE-ABS-KEY ("Performances, Sports") OR TITLE-ABS-KEY ("Sports Performances") OR TITLE-ABS-KEY ("Psychomotor Performance") OR TITLE-ABS-KEY ("Performance, Psychomotor") OR TITLE-ABS-KEY ("Performances, Psychomotor") OR TITLE-ABS-KEY ("Psychomotor Performances") OR TITLE-ABS-KEY ("Perceptual Motor Performance") OR TITLE-ABS-KEY ("Motor Performance, Perceptual") OR TITLE-ABS-KEY ("Motor Performances, Perceptual") OR TITLE-ABS-KEY ("Perceptual Motor Performances") OR TITLE-ABS-KEY ("Performance, Perceptual Motor") OR TITLE-ABS-KEY ("Performances, Perceptual Motor") OR TITLE-ABS-KEY ("Sensory Motor Performance") OR TITLE-ABS-KEY ("Motor Performance, Sensory") OR TITLE-ABS-KEY ("Motor Performances, Sensory") OR TITLE-ABS-KEY ("Performance, Sensory Motor") OR TITLE-ABS-KEY ("Performances, Sensory Motor") OR TITLE-ABS-KEY ("Sensory Motor Performances") OR TITLE-ABS-KEY ("Visual Motor Coordination"))

4. (TITLE-ABS-KEY ("Mental Health") OR TITLE-ABS-KEY ("Health, Mental") OR TITLE-ABS-KEY ("Mental Hygiene") OR TITLE-ABS-KEY ("Hygiene, Mental") OR TITLE-ABS-KEY (anxiety) OR TITLE-ABS-KEY (depression) OR TITLE-ABS-KEY (happ) OR TITLE-ABS-KEY (stress) OR TITLE-ABS-KEY ("mental toughness") OR TITLE-ABS-KEY ("Cognitive abilities") OR TITLE-ABS-KEY (hrv) OR TITLE-ABS-KEY ("psychological

well-being") OR TITLE-ABS-KEY ("mental well-being") OR TITLE-ABS-KEY ("cognitive abililty") OR TITLE-ABS-KEY (emotion) OR TITLE-ABS-KEY (mood))
5. (TITLE-ABS-KEY ("Cognitive performance") OR TITLE-ABS-KEY ("Cognitive function") OR TITLE-ABS-KEY ("Cognitive ability") OR TITLE-ABS-KEY ("Cognitive processing") OR TITLE-ABS-KEY ("Cognitive assessment") OR TITLE-ABS-KEY ("Cognitive load") OR TITLE-ABS-KEY ("Cognitive impairment") OR TITLE-ABS-KEY ("Memory performance") OR TITLE-ABS-KEY (attention) OR TITLE-ABS-KEY ("Neurocognitive performance") OR TITLE-ABS-KEY ("Mental performance") OR TITLE-ABS-KEY ("Cognitive decline") OR TITLE-ABS-KEY ("Cognitive enhancement") OR TITLE-ABS-KEY ("Cognitive training"))
6. (TITLE-ABS-KEY (athletes) OR TITLE-ABS-KEY (athlete) OR TITLE-ABS-KEY ("Professional Athletes") OR TITLE-ABS-KEY ("Athlete, Professional") OR TITLE-ABS-KEY ("Athletes, Professiona") OR TITLE-ABS-KEY ("Professional Athlete") OR TITLE-ABS-KEY ("Elite Athletes") OR TITLE-ABS-KEY ("Athlete, Elite") OR TITLE-ABS-KEY ("Athletes, Elite") OR TITLE-ABS-KEY ("Elite Athlete") OR TITLE-ABS-KEY ("College Athletes") OR TITLE-ABS-KEY ("Athlete, College") OR TITLE-ABS-KEY ("Athletes, College") OR TITLE-ABS-KEY ("College Athlete") OR TITLE-ABS-KEY (players) OR TITLE-ABS-KEY (players) OR TITLE-ABS-KEY (runners) OR TITLE-ABS-KEY (cyclists) OR TITLE-ABS-KEY (boxers) OR TITLE-ABS-KEY (shooters) OR TITLE-ABS-KEY (wrestlers) OR TITLE-ABS-KEY (golfers)) Athlete))
7.1OR2
8.3OR4OR5
9.6AND7AND8

5.web of science
1.Biofeedback, Psychology (Topic) or Biofeedbacks, Psychology (Topic) or Psychology Biofeedback (Topic) or Psychology Biofeedbacks (Topic) or Biofeedback (Topic) or Biofeedbacks (Topic) or Biofeedback (Psychology) (Topic) or Biofeedbacks (Psychology) (Topic) or Feedback, Psychophysiologic (Topic) or Feedback, Psychophysiological (Topic) or Psychophysiologic Feedback (Topic) or False Physiological Feedback (Topic) or False Physiological Feedbacks (Topic) or Feedback, False Physiological (Topic) or Feedbacks, False Physiological (Topic) or Physiological Feedback, False (Topic) or Physiological Feedbacks, False (Topic) or Bogus Physiological Feedback (Topic) or Bogus Physiological Feedbacks (Topic) or Feedback, Bogus Physiological (Topic) or Feedbacks, Bogus Physiological (Topic) or Physiological Feedback, Bogus (Topic) or Physiological Feedbacks, Bogus (Topic) or Myofeedback (Topic) or Myofeedbacks (Topic)
2.Neurofeedback (Topic) or Neurofeedbacks (Topic) or Brainwave Biofeedback (Topic) or Biofeedback, Brainwave (Topic) or Biofeedbacks, Brainwave (Topic) or Brainwave Biofeedbacks (Topic) or Alpha Biofeedback (Topic) or Alpha Biofeedbacks (Topic) or Biofeedback, Alpha (Topic) or Biofeedbacks, Alpha (Topic) or Alpha Feedback (Topic) or Alpha Feedbacks (Topic) or Feedback, Alpha (Topic) or Feedbacks, Alpha (Topic) or Brainwave Feedback (Topic) or Brainwave Feedbacks (Topic) or Feedback, Brainwave (Topic) or Feedbacks, Brainwave (Topic) or EEG Feedback (Topic) or EEG Feedbacks (Topic) or Feedback, EEG (Topic) or Feedbacks, EEG (Topic) or Electroencephalography Biofeedback

(Topic) or Biofeedback, Electroencephalography (Topic) or Biofeedbacks, Electroencephalography (Topic) or Electroencephalography Biofeedbacks (Topic) or Electromyography Feedback (Topic) and Preprint Citation Index (Exclude – Database)

3.Athletic Performance (Topic) or Athletic Performances (Topic) or Performance, Athletic (Topic) or Performances, Athletic (Topic) or Sports Performance (Topic) or Performance, Sports (Topic) or Performances, Sports (Topic) or Sports Performances (Topic) or Psychomotor Performance (Topic) or Performance, Psychomotor (Topic) or Performances, Psychomotor (Topic) or Psychomotor Performances (Topic) or Perceptual Motor Performance (Topic) or Motor Performance, Perceptual (Topic) or Motor Performances, Perceptual (Topic) or Perceptual Motor Performances (Topic) or Performance, Perceptual Motor (Topic) or Performances, Perceptual Motor (Topic) or Sensory Motor Performance (Topic) or Motor Performance, Sensory (Topic) or Motor Performances, Sensory (Topic) or Performance, Sensory Motor (Topic) or Performances, Sensory Motor (Topic) or Sensory Motor Performances (Topic) or Visual Motor Coordination (Topic) or Coordinations, Visual Motor (Topic) or Coordination, Visual Motor (Topic) or Motor Coordinations, Visual (Topic) or Motor Coordination, Visual (Topic) or Visual Motor Coordinations (Topic) and Preprint Citation Index (Exclude – Database)

4.Mental Health (Topic) or Health, Mental (Topic) or Mental Hygiene (Topic) or Hygiene, Mental (Topic) or Anxiety (Topic) or depression (Topic) or happ (Topic) or Stress (Topic) or mental toughness (Topic) or Cognitive abilities (Topic) or HRV (Topic) or psychological well-being (Topic) or mental well-being (Topic) or cognitive abililty (Topic) or Emotion (Topic) or Mood (Topic) and Preprint Citation Index (Exclude – Database)

5.Cognitive performance (Topic) or Cognitive function (Topic) or Cognitive ability (Topic) or Cognitive processing (Topic) or Cognitive assessment (Topic) or Cognitive load (Topic) or Cognitive impairment (Topic) or Executive function (Topic) or Memory performance (Topic) or Attention (Topic) or Neurocognitive performance (Topic) or Mental performance (Topic) or Cognitive decline (Topic) or Cognitive enhancement (Topic) or Cognitive training (Topic) and Preprint Citation Index (Exclude – Database)

6.Athletes (Topic) or Athlete (Topic) or Professional Athletes (Topic) or Athlete, Professional (Topic) or Athletes, Professional (Topic) or Professional Athlete (Topic) or Elite Athletes (Topic) or Athlete, Elite (Topic) or Athletes, Elite (Topic) or Elite Athlete (Topic) or College Athletes (Topic) or Athlete, College (Topic) or Athletes, College (Topic) or College Athlete (Topic) or disabled athletes (Topic) or boxers (Topic) or tennis players (Topic) or rugby players (Topic) or wrestlers (Topic) or golfers (Topic) or cyclists (Topic) or para-athletes (Topic) and Preprint Citation Index (Exclude – Database)

7.#1 OR #2 and Preprint Citation Index (Exclude – Database)

8.#3 OR #4 OR #5 and Preprint Citation Index (Exclude – Database)

9.#6 AND #7 AND #8 and Preprint Citation Index (Exclude – Database)

6.Sportdiscus

1.(biofeedback* OR "biofeedback therapy" OR "biofeedback intervention") OR TX (biofeedback* OR "biofeedback therapy" OR "biofeedback intervention") OR (neurofeedback* OR "neurofeedback therapy") OR TX (neurofeedback* OR)

22.(exerci* OR exercise* OR "athletic performance" OR athletic* OR sports* OR sport* OR

"sports performance" OR strength* OR "muscle strength" OR "endurance performance" OR endurance* OR agility* OR "cognitive ability" OR "response time" OR aerobic* OR anaerobic*)
3.("mental health" OR happ* OR anxiety* OR depression* OR stress* OR "mental toughness OR motivation* OR happiness* OR "psychological well-being" OR "mental well-being" OR flow* OR pain* OR panic* OR anger* OR vividness* OR mood* OR emotion* OR concentration* OR HRV* OR cortisol*)
4.("Cognitive Ability" OR "Cognitive Function" OR "Cognitive Assessment" OR "Cognitive Impairment" OR "Cognitive Training" OR "Working Memory" OR "Attention Control" OR "decision-making ability" OR "information processing speed" OR "learning and memory" OR "executive function" OR "cognitive load")
5.S2 OR S3 OR S4
6.(athletes* OR players* OR "para-athletes" OR cyclists* OR runners* OR golfers* OR wrestlers* OR "rugby players" OR "tennis players" OR boxers* OR "disabled athletes" OR shooters*)
7.S1 AND S5 AND S6

7.Embase

1.'biofeedback, psychology'/exp OR 'biofeedback, psychology' OR (('biofeedback,'/exp OR biofeedback,) AND ('psychology'/exp OR psychology)) OR 'biofeedbacks, psychology' OR 'psychology biofeedback' OR 'psychology biofeedbacks' OR 'biofeedback'/exp OR biofeedback OR biofeedbacks OR 'biofeedback (psychology)' OR 'biofeedbacks (psychology)' OR 'feedback, psychophysiological' OR 'psychophysiologic feedback' OR 'false physiological feedback' OR 'false physiological feedbacks' OR 'feedback, false physiological' OR 'feedbacks, false physiological' OR 'physiological feedback, false' OR 'physiological feedbacks, false' OR 'bogus physiological feedback' OR 'bogus physiological feedbacks' OR 'feedback, bogus physiological' OR 'feedbacks, bogus physiological' OR 'physiological feedback, bogus' OR 'physiological feedbacks, bogus' OR 'myofeedback'/exp OR myofeedback OR myofeedbacks
2.'neurofeedback'/exp OR neurofeedback OR neurofeedbacks OR 'brainwave biofeedback' OR 'biofeedback, brainwave' OR 'biofeedbacks, brainwave' OR 'brainwave biofeedbacks' OR 'alpha biofeedback' OR 'alpha biofeedbacks' OR 'biofeedback, alpha' OR 'pbiofeedbacks, alpha' OR 'alpha feedback'/exp OR 'alpha feedback' OR 'alpha feedbacks' OR 'feedback, alpha' OR 'feedbacks, alpha' OR 'brainwave feedback' OR 'pbrainwave feedbacks' OR 'feedback, brainwave' OR 'feedbacks, brainwave' OR 'eeg feedback'/exp OR 'eeg feedback' OR 'eeg feedbacks' OR 'feedback, eeg' OR 'feedbacks, eeg' OR 'electroencephalography biofeedback' OR 'biofeedback, electroencephalography' OR 'biofeedbacks, electroencephalography' OR 'electroencephalography biofeedbacks' OR 'electromyography feedback'/exp OR 'electromyography feedback'
3.'athletic performance'/exp OR 'athletic performance' OR 'athletic performances' OR 'performance, athletic' OR 'performances, athletic' OR 'sports performance'/exp OR 'sports performance' OR 'performance, sports' OR 'performances, sports' OR 'sports performances' OR 'psychomotor performance' OR 'performance, psychomotor' OR 'performances, psychomotor' OR 'psychomotor performances' OR 'perceptual motor performance' OR

'motor performance, perceptual' OR 'motor performances, perceptual' OR 'perceptual motor performances' OR 'performance, perceptual motor' OR 'performances, perceptual motor' OR 'sensory motor performance' OR 'motor performance, sensory' OR 'motor performances, sensory' OR 'performance, sensory motor' OR 'performances, sensory motor' OR 'sensory motor performances' OR 'visual motor coordination'/exp OR 'visual motor coordination' OR 'coordinations, visual motor' OR 'coordination, visual motor' OR 'motor coordinations, visual' OR 'motor coordination, visual' OR 'visual motor coordinations'

4.'mental health'/exp OR 'mental health' OR 'health, mental' OR 'mental hygiene'/exp OR 'mental hygiene' OR 'hygiene, mental' OR 'anxiety'/exp OR anxiety OR 'depression'/exp OR depression OR happ OR 'stress'/exp OR stress OR 'mental toughness'/exp OR 'mental toughness' OR 'cognitive abilities' OR hrv OR 'psychological well-being'/exp OR 'psychological well-being' OR 'mental well-being'/exp OR 'mental well-being' OR 'cognitive abililty' OR 'emotion'/exp OR emotion OR 'mood'/exp OR mood

5.'cognitive performance'/exp OR 'cognitive performance' OR 'cognitive function'/exp OR 'cognitive function' OR 'cognitive ability'/exp OR 'cognitive ability' OR 'cognitive processing' OR 'cognitive assessment'/exp OR 'cognitive assessment' OR 'cognitive load'/exp OR 'cognitive load' OR 'cognitive impairment'/exp OR 'cognitive impairment' OR 'executive function'/exp OR 'executive function' OR 'memory performance'/exp OR 'memory performance' OR 'attention'/exp OR attention OR 'neurocognitive performance'/exp OR 'neurocognitive performance' OR 'mental performance'/exp OR 'mental performance' OR 'cognitive decline'/exp OR 'cognitive decline' OR 'cognitive enhancement'/exp OR 'cognitive enhancement' OR 'cognitive training'/exp OR 'cognitive training'

6.'athletes'/exp OR athletes OR athlete OR 'professional athletes' OR 'athlete, professional' OR 'athletes, professional' OR 'professional athlete'/exp OR 'professional athlete' OR 'elite athletes' OR 'athlete, elite' OR 'athletes, elite' OR 'elite athlete'/exp OR 'elite athlete' OR 'college athletes' OR 'athlete, college' OR 'athletes, college' OR 'college athlete'/exp OR 'college athlete' OR 'cognitive training'

7.#1 OR #2

8.#3 OR #4 OR #5

9.#6 AND #7 AND #8

8.CNKI

1.（主题：生物反馈）OR（全文：生物反馈(精确)）OR（主题：神经反馈）OR（全文：神经反馈(精确)）

2.（主题：运动表现）OR（全文：运动表现(精确)）OR（主题：心理健康）OR（全文：心理健康(精确)）OR（主题：认知表现）OR（全文：认知表现(精确)）

3.（主题：运动员）OR（全文：运动员(精确)）

4.1AND2AND3
