# Supplementary material for: The effects of biofeedback training on athletes’ mental health and performance: a systematic review and Bayesian meta-analysis
Source: Front Psychol. 2025 Oct 21;16:1662868. doi: 10.3389/fpsyg.2025.1662868 (PMC12583207; doi:10.3389/fpsyg.2025.1662868)
Supplement: Supplementary file 1 [file Data_Sheet_1.ZIP › Supplementary file S3 Risk of Bias in Individual Studies.pdf]

Table 1: <i> Supplement File. Risk of Bias in Individual Studies </i>

Author	Sequence generation	Allocation concealment	Blinding of Participants and Personnel	Blinding outcome assessment	Incomplete outcome	Selective reporting	Other bias	Overall bias
Alonazi	Low	Unclear	Low	Unclear	Low	Low	Low	Low
AnA	Low	Unclear	Unclear	Unclear	Low	Low	Low	Medium
AnB	Low	Unclear	Unclear	Unclear	Low	Low	Low	Medium
Bakhtafrooz	Unclear	High	Unclear	Unclear	Low	Low	Low	High
Canton	Low	Unclear	Low	Unclear	Low	Low	Low	Low
Cheng	Low	Unclear	Unclear	Low	Low	Low	Low	Low
Choudhary	Low	Unclear	Unclear	Unclear	Low	Low	Low	Medium
Cummings	Low	Unclear	Unclear	Unclear	Low	Low	Low	Medium
Dana	Low	Unclear	Unclear	Unclear	Low	Low	Low	Medium
Domingos	Low	Unclear	Unclear	LowUnclear	Low	Low	Low	Medium
Donghai	Unclear	Unclear	High	Unclear	Low	Unclear	Low	High
Dziembowska	Low	Low	Unclear	Low	Low	Low	Unclear	Low
EliA	Low	Low	Unclear	Low	Low	Low	Low	Low
EliB	Low	Unclear	Unclear	Low	Low	Low	Low	Low
Faridnia	Low	Unclear	Unclear	Low	Low	Low	Low	Low
Firth	Low	Unclear	Unclear	Low	Low	Low	Low	Low
Gorman	Low	Unclear	High	Unclear	Low	Low	Low	High
JaeYo	Low	Unclear	Unclear	Unclear	Low	Low	Low	Medium
Jung	Low	Unclear	Unclear	Unclear	Low	Low	Low	Medium
Kilding	Low	Unclear	Low	High	Low	Low	Low	High
Makaracl	Low	Unclear	Unclear	Unclear	Low	Low	Low	Medium
Maszczyk	Low	Unclear	Low	Low	Low	Low	Low	Low
Mikicin	Low	Unclear	High	Unclear	Low	Low	Low	High
Mirifar	Low	Unclear	Low	Unclear	Low	Low	Low	Low
Paula	Low	Unclear	Low	Unclear	Low	Low	Low	Low
Paulb	Low	Unclear	Low	Low	Low	Low	Low	Low
Pronczuk	Low	Low	Unclear	Unclear	Low	Low	Low	Low
Ram	Low	Unclear	Unclear	Unclear	Low	Low	Low	Medium

Author	Sequence generation	Allocation concealment	Blinding of Participants and Personnel	Blinding outcome assessment	Incomplete outcome	Selective reporting	Other bias	Overall bias
Ring	Low	Unclear	Low	Low	Low	Low	Low	Low
Rusciano	Low	Unclear	Low	Unclear	Low	Low	Low	Low
Saha	Low	Unclear	Unclear	Unclear	Low	Low	Low	Medium
Srilekha	Low	Unclear	Unclear	Unclear	Low	Low	Low	Medium
Thomas	Low	Unclear	Unclear	Unclear	Low	Low	Low	Medium
Tirinnanzi	Low	Unclear	Unclear	Low	Low	Low	Low	Low
Vacher	Low	Unclear	Unclear	Unclear	Low	Low	Low	Medium
ViettaEA	Low	Low	Unclear	Unclear	Low	Low	Unclear	Medium
ViettaEB	Low	Low	Unclear	Unclear	Low	Low	Unclear	Medium
Yalfani	Low	Unclear	Unclear	High	Low	Low	Low	High
Yilmaz	Unclear	Unclear	Unclear	Unclear	Low	Low	Low	High
Zahir	Low	Unclear	Unclear	Unclear	Low	Low	Low	Medium
