# Supplementary material for: The effects of biofeedback training on athletes’ mental health and performance: a systematic review and Bayesian meta-analysis
Source: Front Psychol. 2025 Oct 21;16:1662868. doi: 10.3389/fpsyg.2025.1662868 (PMC12583207; doi:10.3389/fpsyg.2025.1662868)
Supplement: Supplementary file 1 [file Data_Sheet_1.ZIP › Supplementary file S4 Forest plots.docx]

**Subgroup Analysis based on Intervention Type (****Biofeedback vs** **Neurofeedback)**


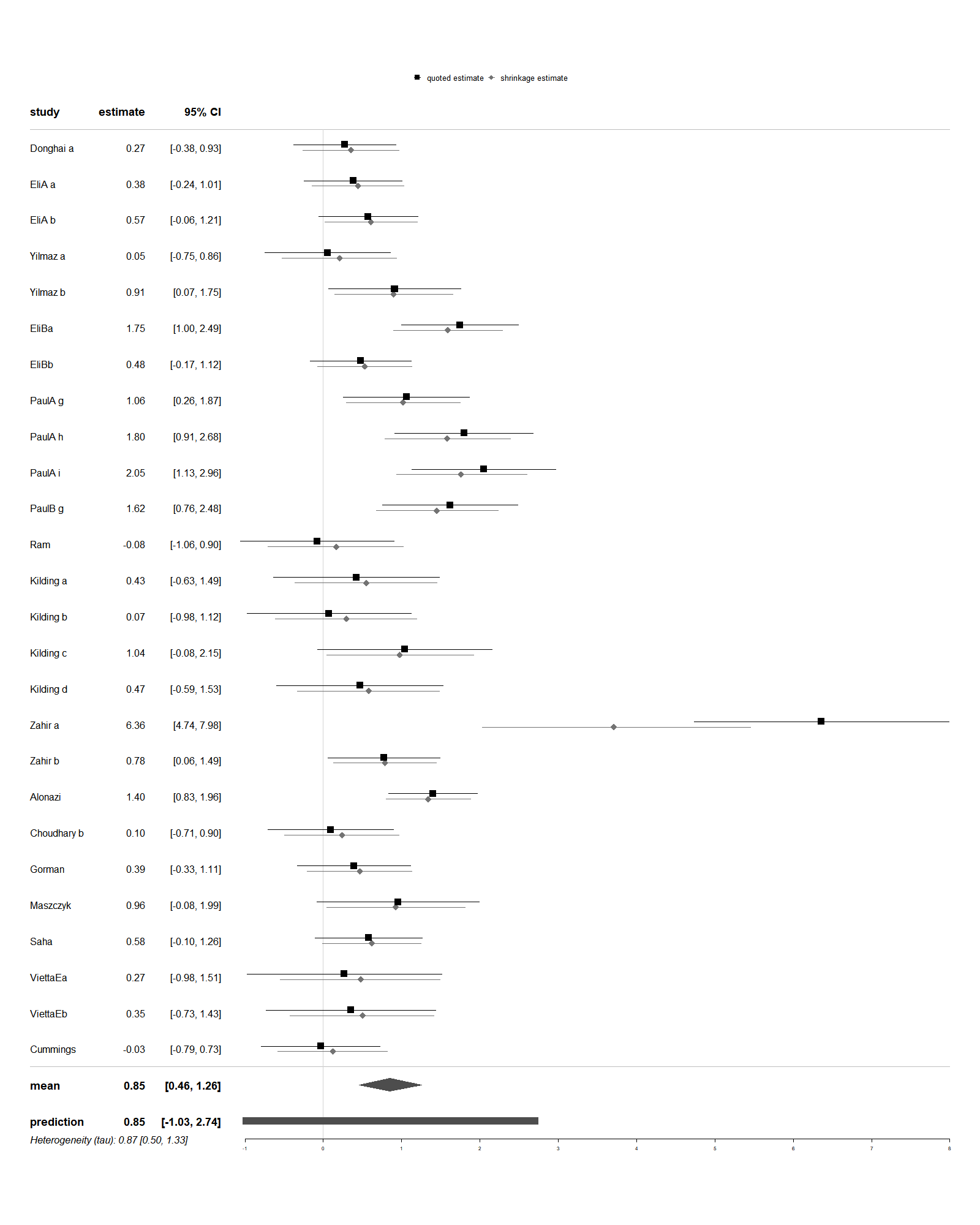


**Figure 1.** The Forest Plot in Athletic Performance Biofeedback

**
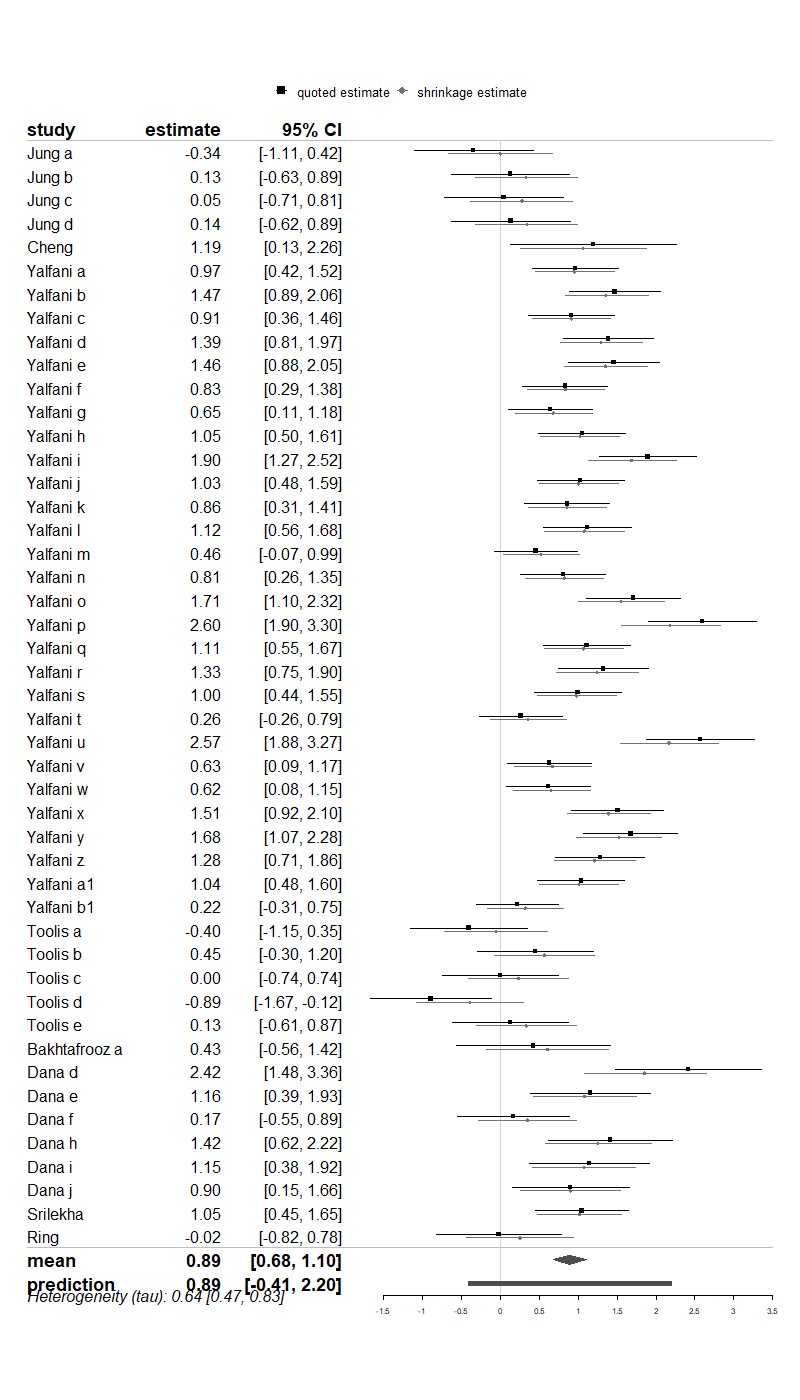
**

**Figure 2.** The Forest Plot in Athletic Performance Neurofeedback


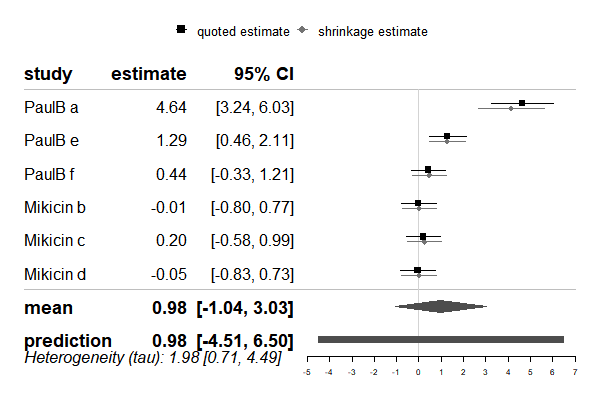


**F****igure 3.** The Forest Plot in Cognitive Performance Biofeedback

**
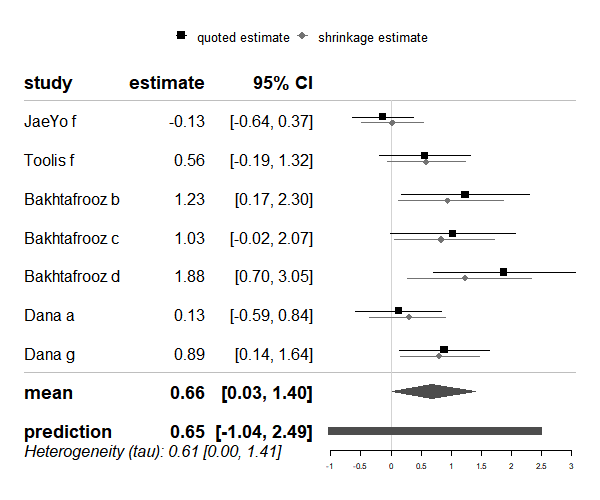
**

**Figure 4.** The Forest Plot in Cognitive Performance Neurofeedback

**Subgroup analysis based on specific psychological and Performance outcomes**

**
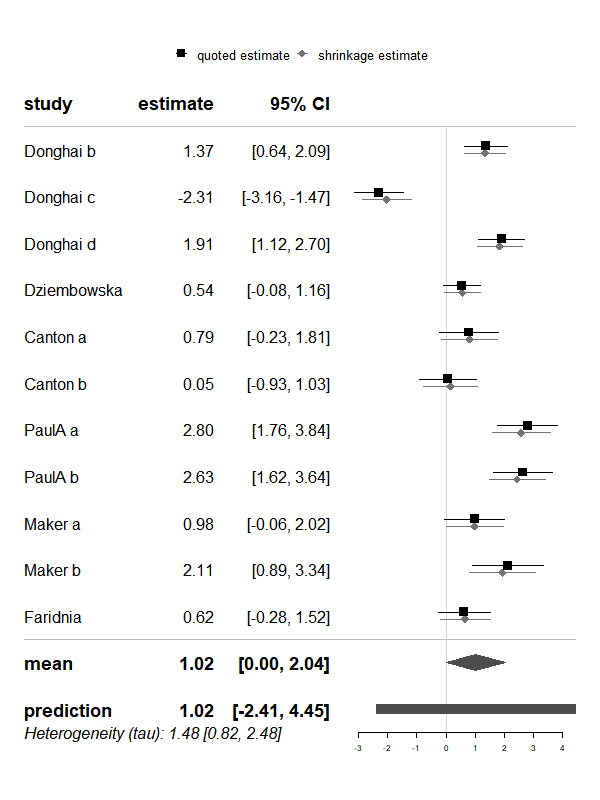
**

**Figure 5.** The Forest Plot in Anxiety Biofeedback

**
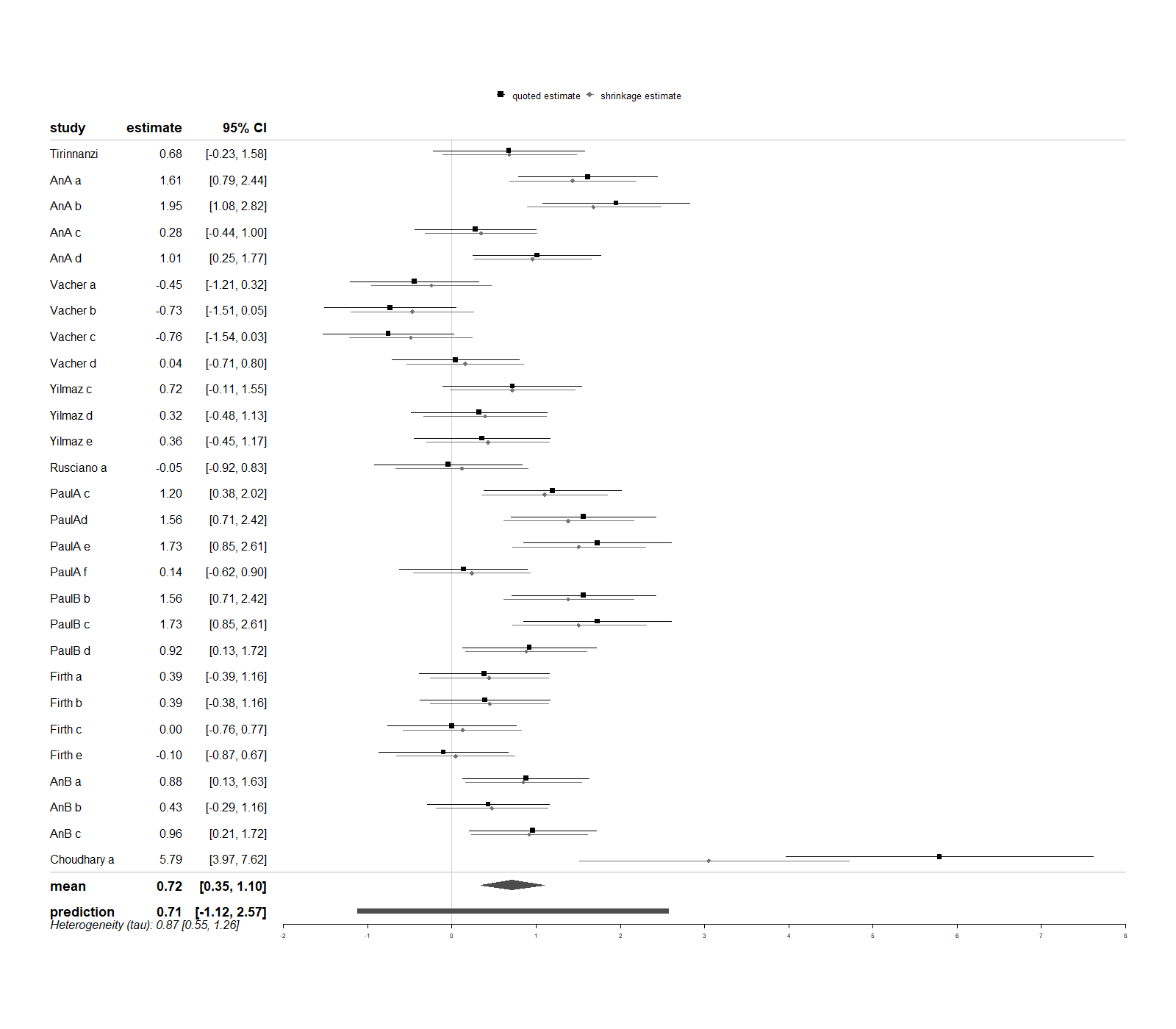
**

**Figure 6.** The Forest Plot in Basketball Performance Biofeedback

**
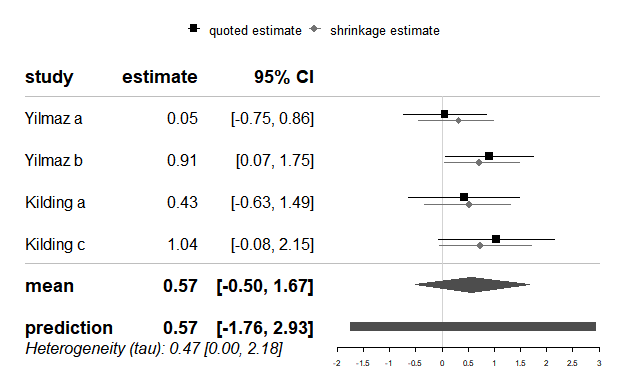
**

**Figure 7.** The Forest Plot in Endurance Biofeedback

**
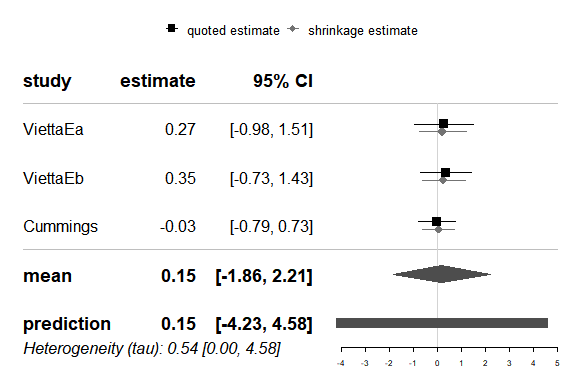
**

**Figure 8.** The Forest Plot in Flexible Biofeedback


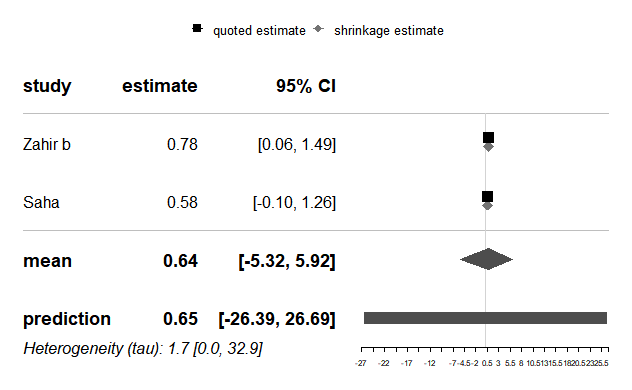


**Figure 9.** The Forest Plot in Football Performance Biofeedback

**
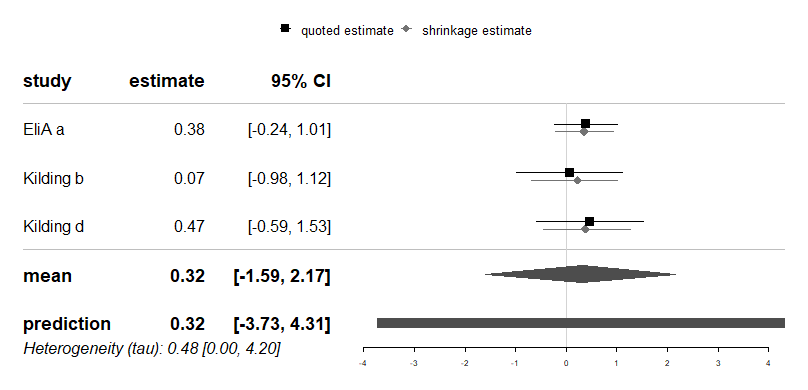
**

**Figure 10.** The Forest Plot in Speed Biofeedback

**
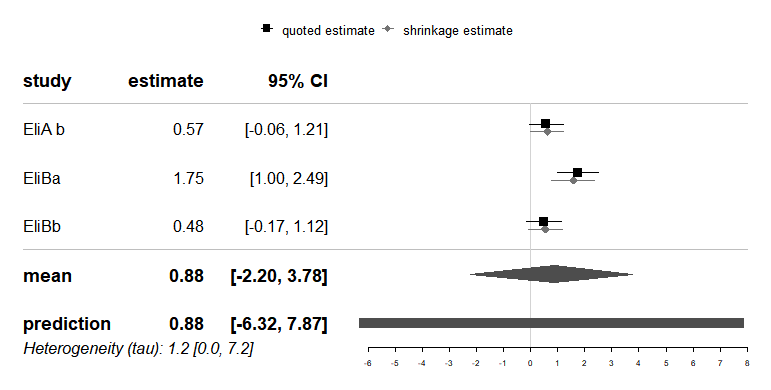
**

**Figure 11.** The Forest Plot in Swimming Performance Biofeedback


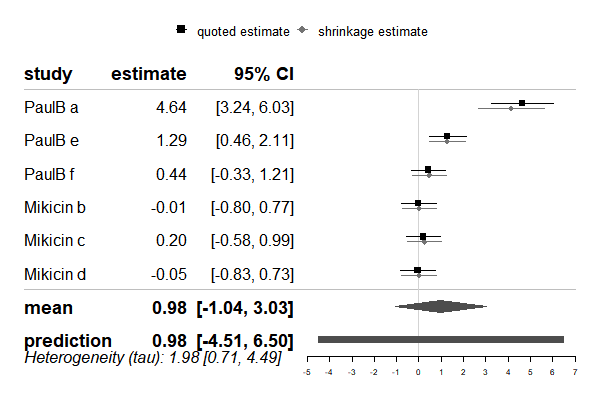


**Figure** **12.** The Forest Plot in Attentional Control Biofeedback


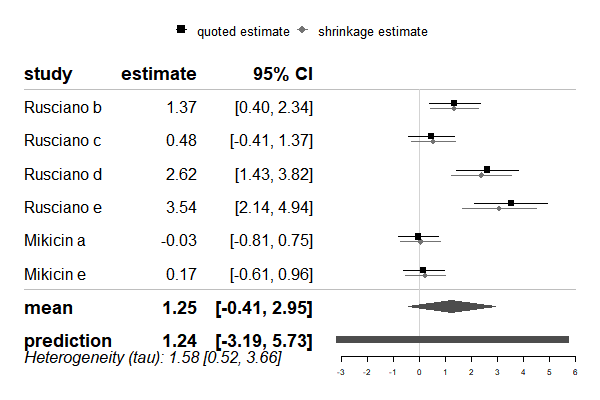


**Figure 13.** The Forest Plot in Selective Attention Biofeedback


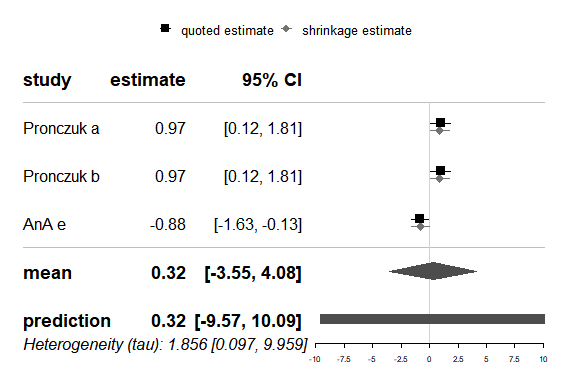


**Figure 14.** The Forest Plot in Task Performance Metrics Biofeedback


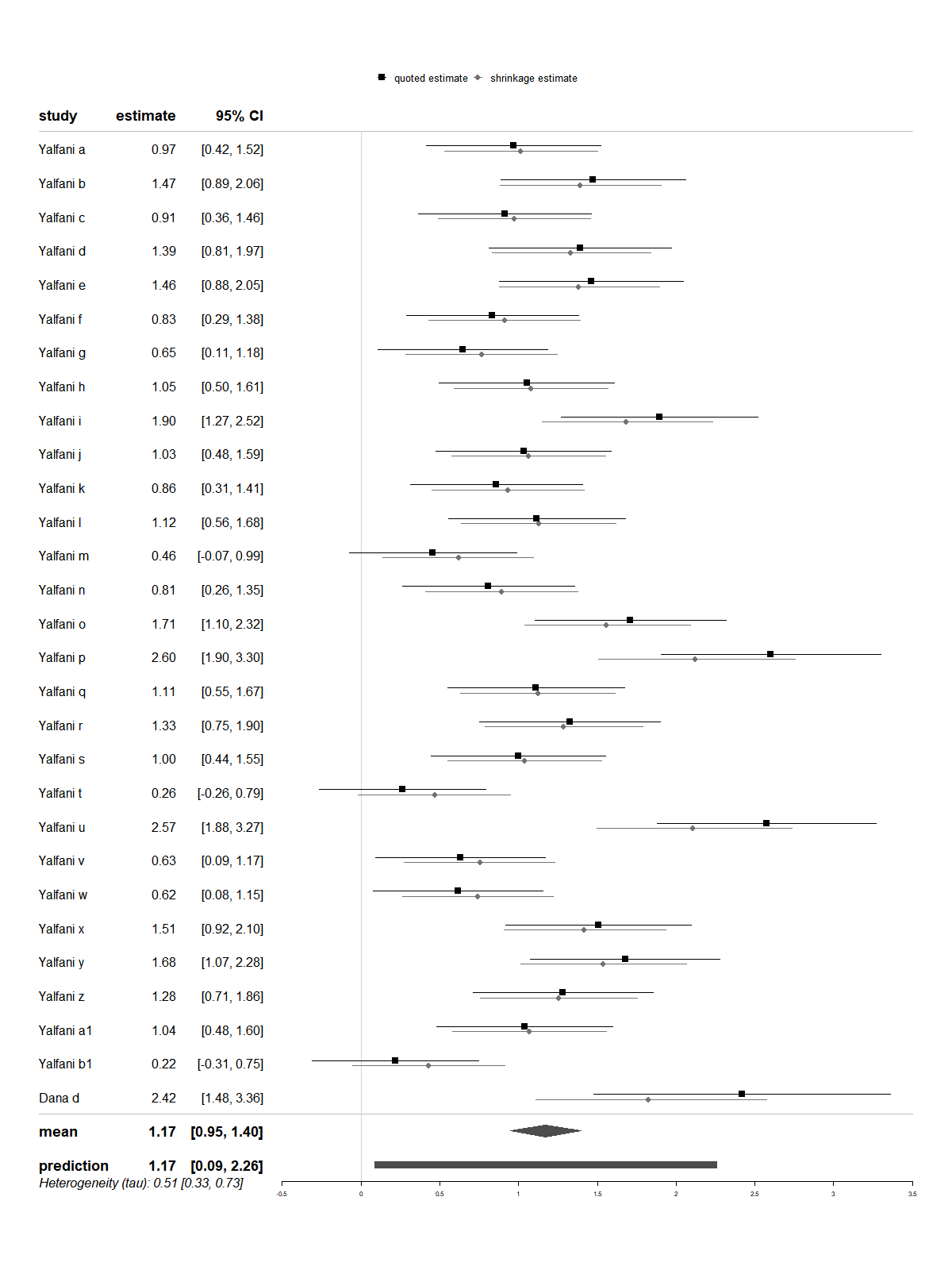


**Figure 15.** The Forest Plot in Task Balance Neurofeedback


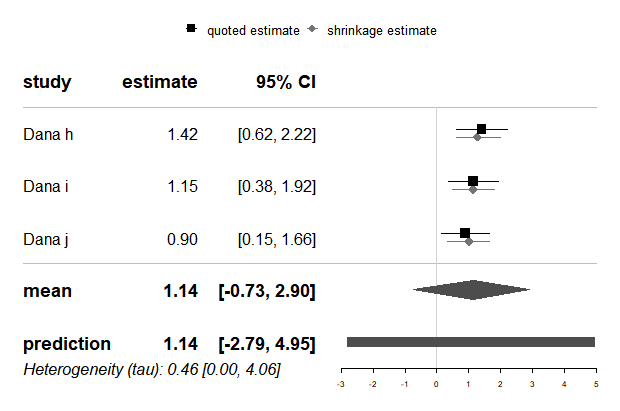


**Figure 16.** The Forest Plot in Coordination Neurofeedback


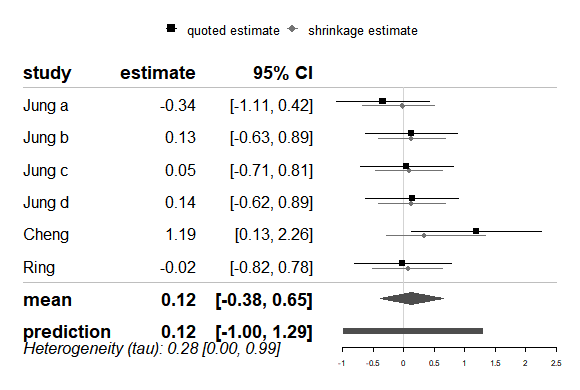


**Figure 17.** The Forest Plot in Golf Performance Neurofeedback


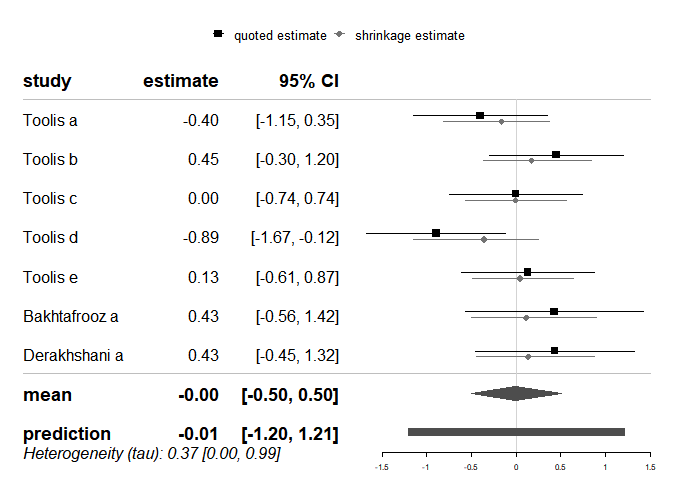


**Figure 18.** The Forest Plot in Shooting_Performance Neurofeedback


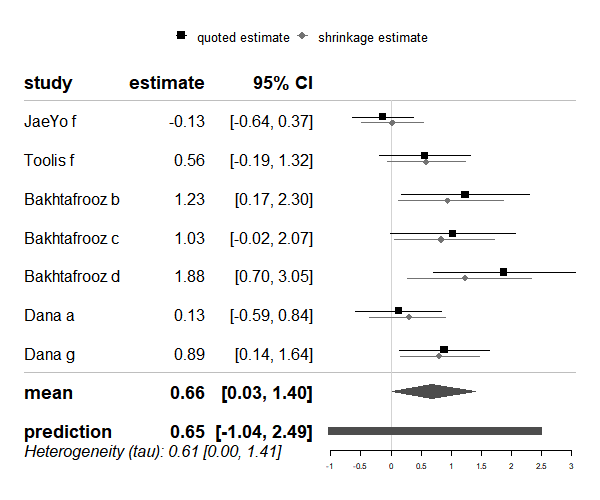


**Figure 19.** The Forest Plot in Attentional_Control Neurofeedback


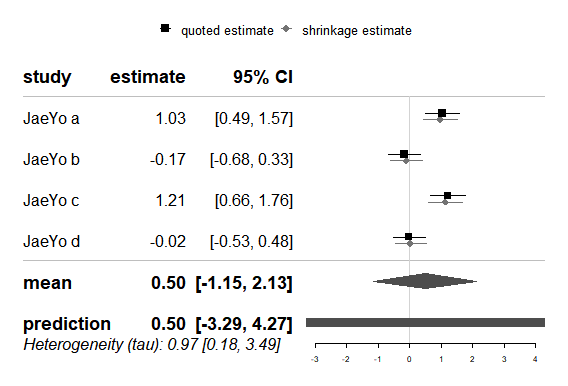


**Figure 20.** The Forest Plot in Attentional_Focus Neurofeedback

**Subgroup Analysis Based on Biofeedback Dose**


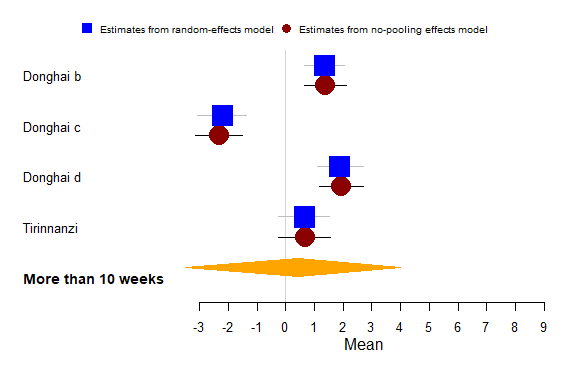


**Figure 21.** The Forest Plot in More Than 10 Weeks Mental Health


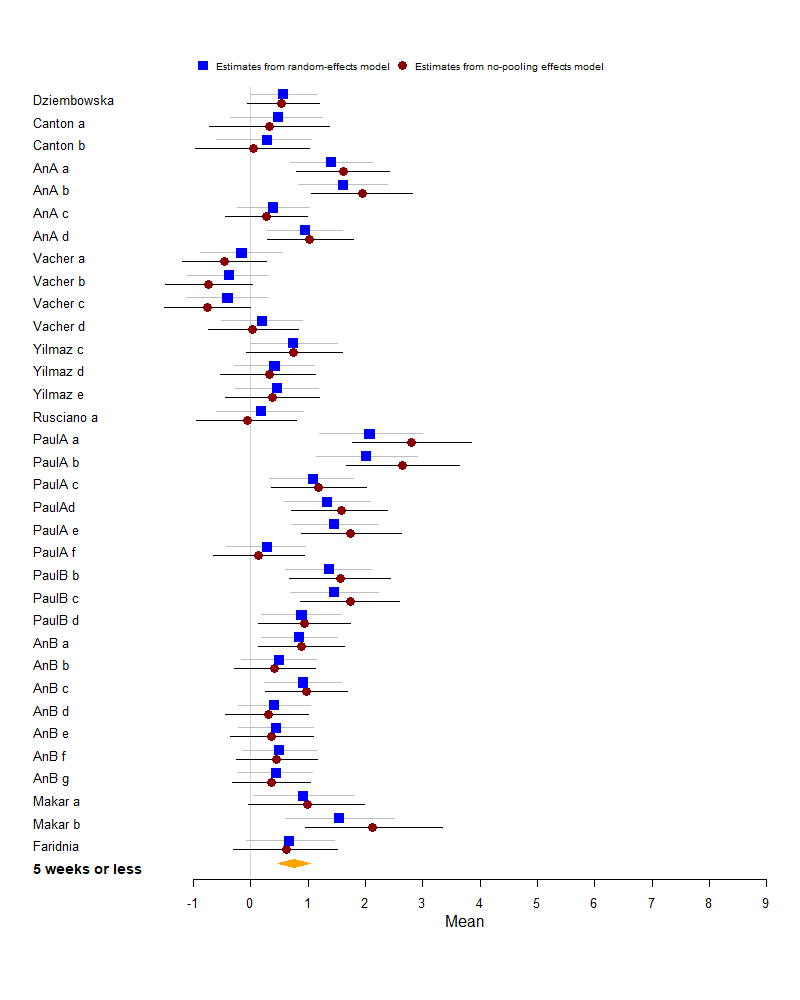


**Figure 22.** The Forest Plot in 5 Weeks or Less Mental Health

**
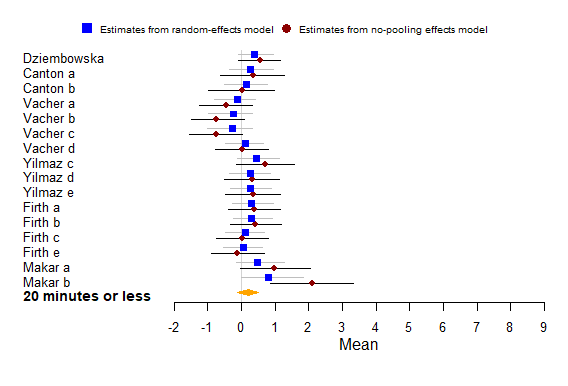
**

**Figure 23.** The Forest Plot in 20 Minutes or Less Mental Health


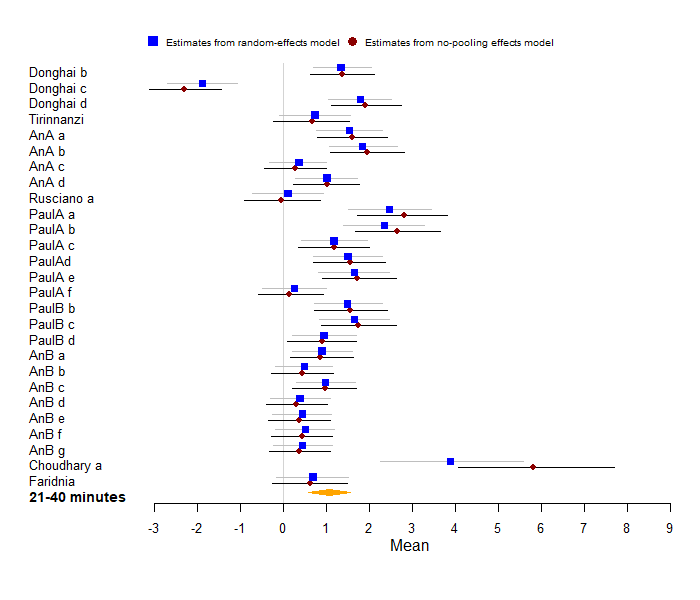


**Figure 24.** The Forest Plot in 21-40 Minute Mental Health


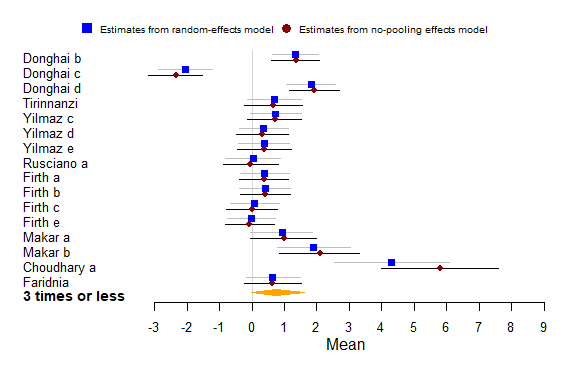


**Figure 25.** The Forest Plot in 3 Times or Less Mental Health


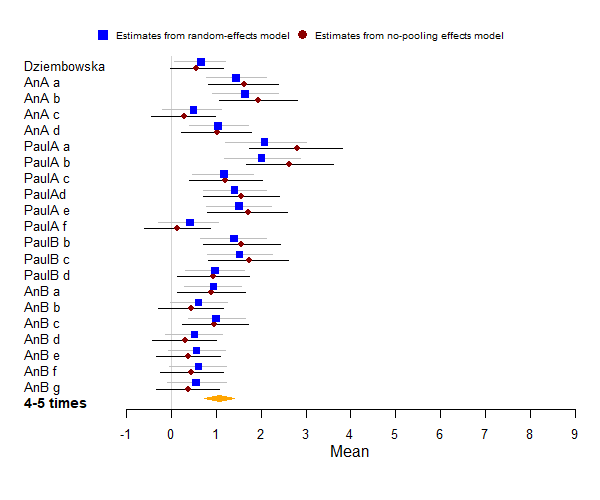


**Figure 26.** The Forest Plot in 4-5 Times Mental Health


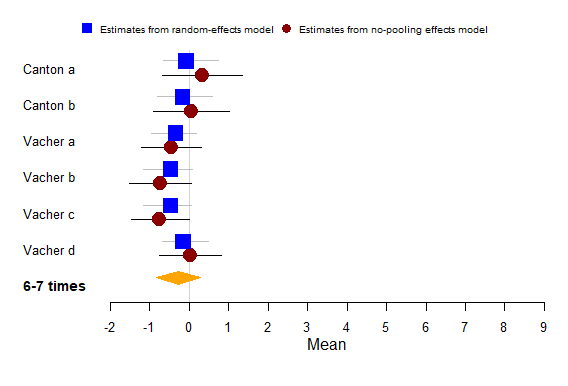


**Figure 27.** The Forest Plot in 6-7 Times Mental Health


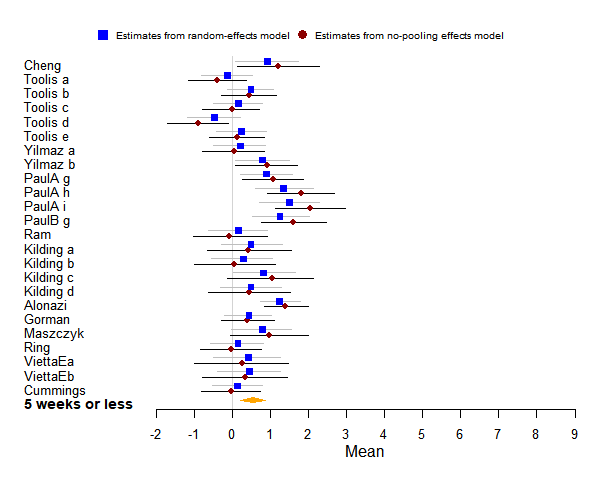


**Figure 28.** The Forest Plot in 5 Weeks or Less Athletic Performance

**
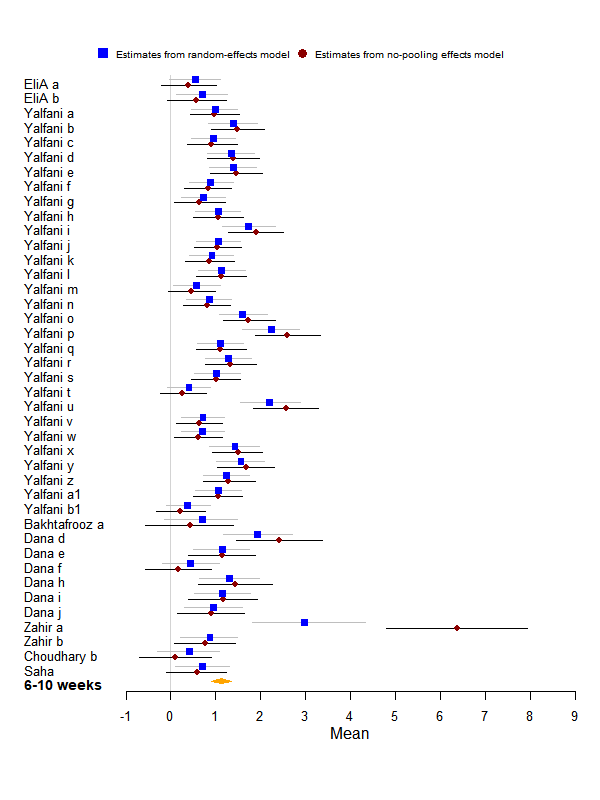
**

**Figure 29.** The Forest Plot in 6-10 Weeks Athletic Performance


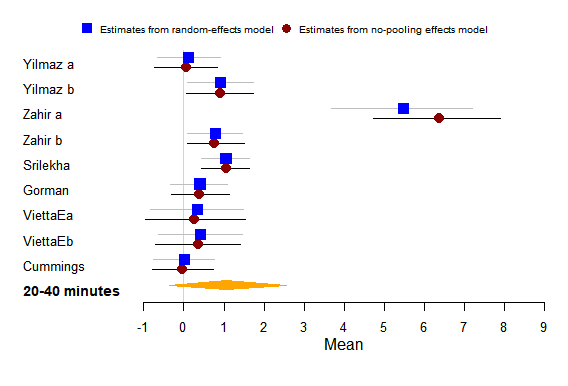


**Figure 30.** The Forest Plot in 20 Minutes or Less Athletic Performance


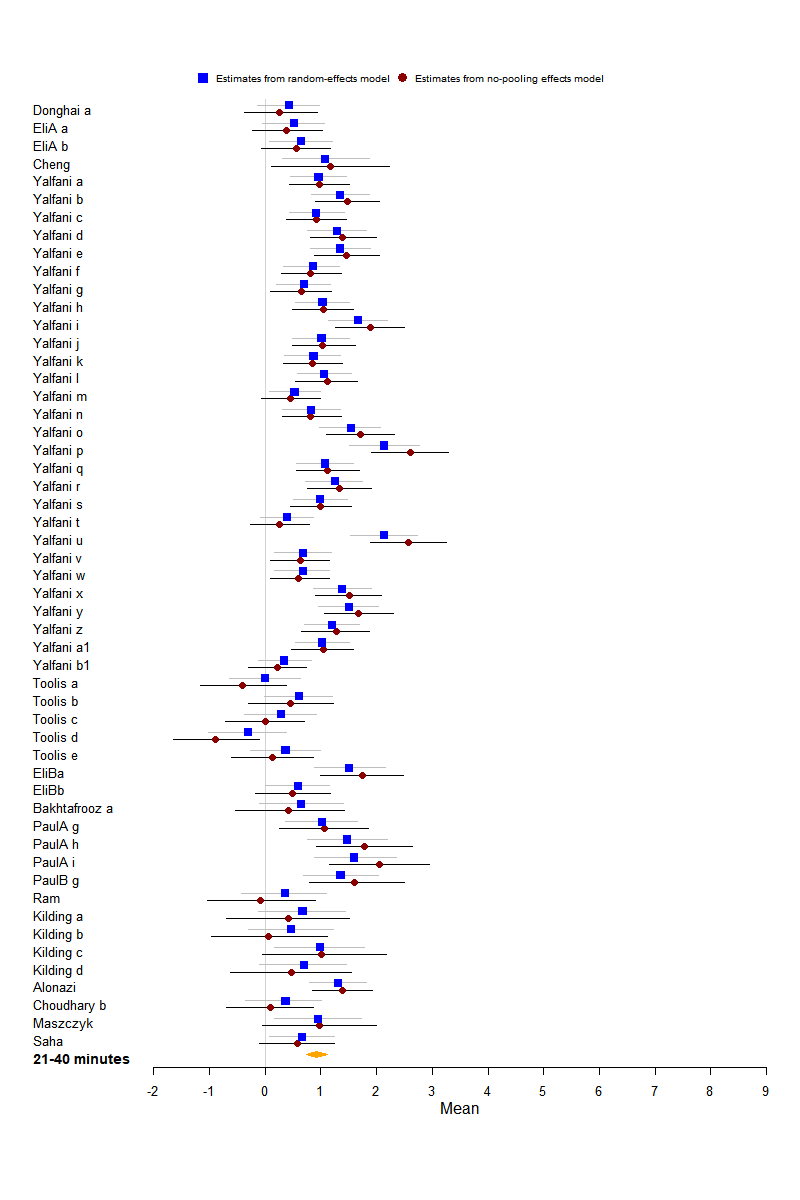


**Figure** **31.** The Forest Plot in 21-40 Minutes Athletic Performance

**
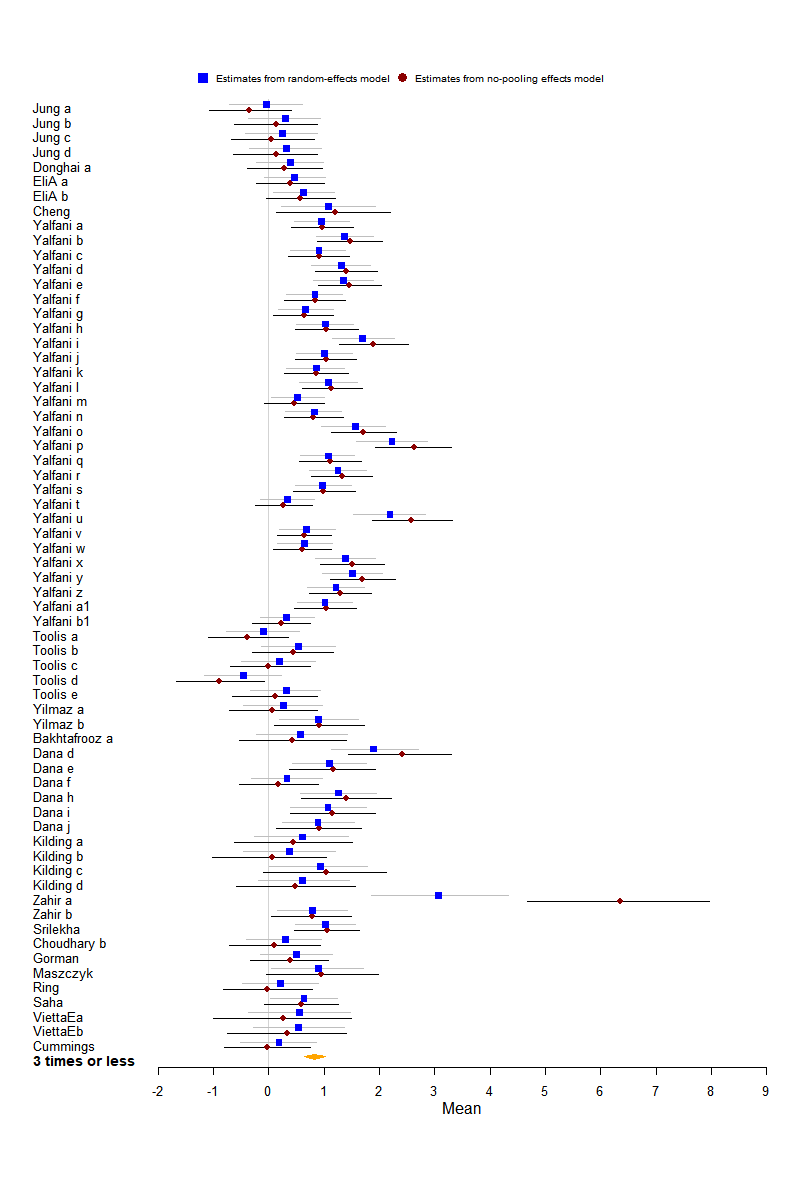
**

**Figure 32.** The Forest Plot in 3 Times or Less Athletic Performance


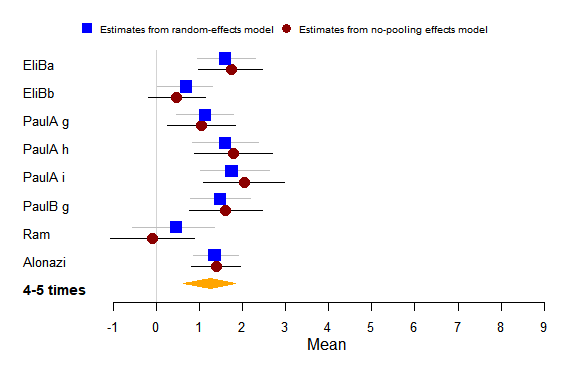


**Figure 33.** The Forest Plot in 4-5 Times Athletic Performance


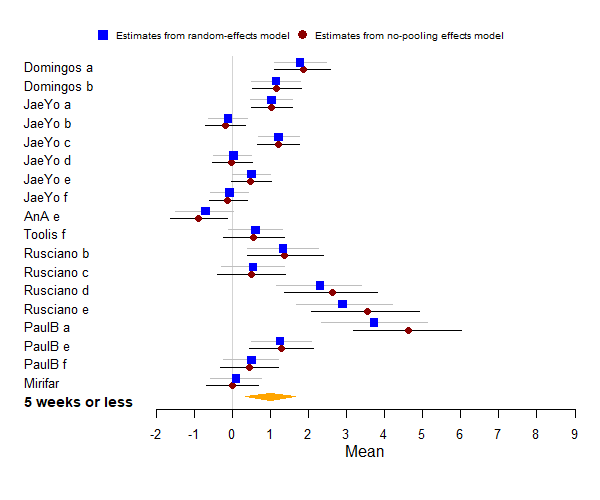


**Figure 34.** The Forest Plot in 5 Weeks or Less Cognitive Performance


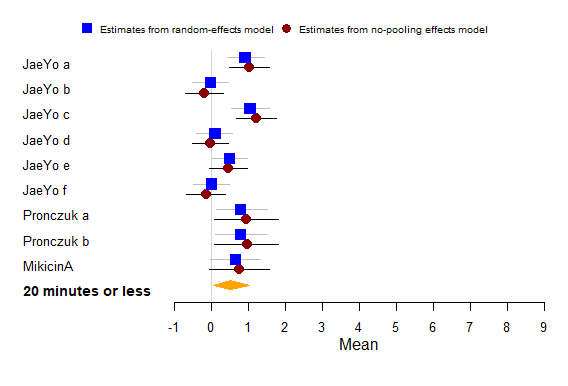


**Figure 35.** The Forest Plot in 20 Minutes or Less Cognitive Performance


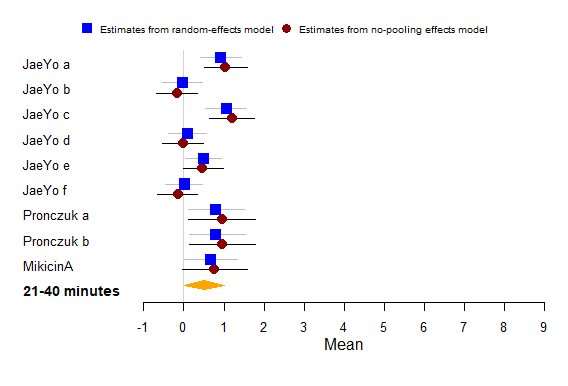


**Figure 36.** The Forest Plot in 21-40 Minutes Cognitive Performance


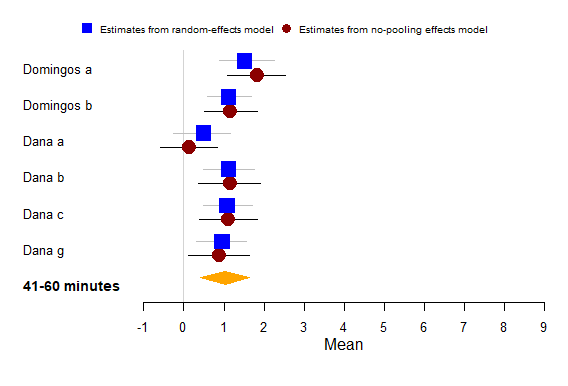


**Fi****gure 37.** The Forest Plot in 41-60 Minutes Cognitive Performance


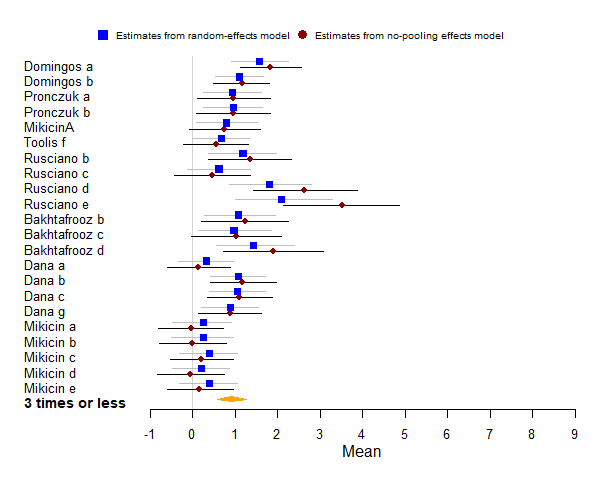


**Figure 38.** The Forest Plot in 3 Times or Less Cognitive Performance


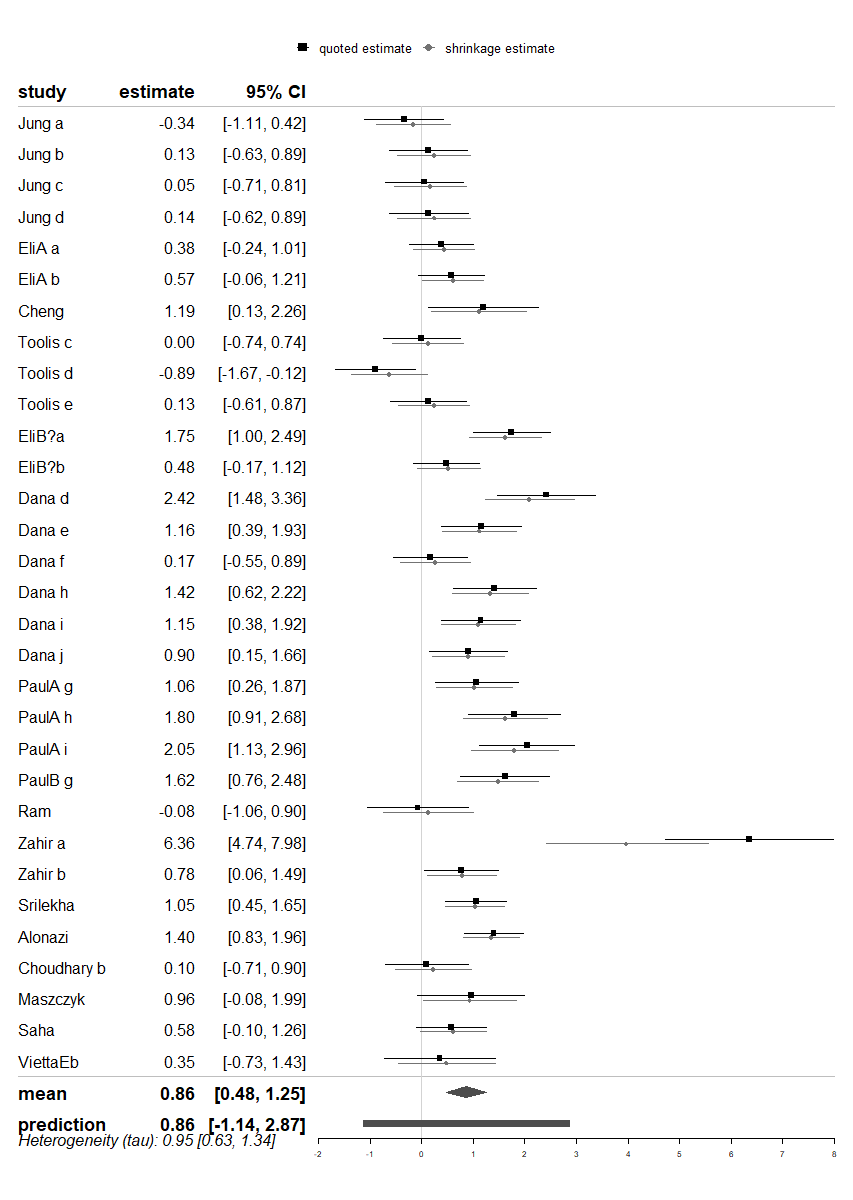


**Figure 39.** The Forest Plot in Athletic performance Adequate blinding


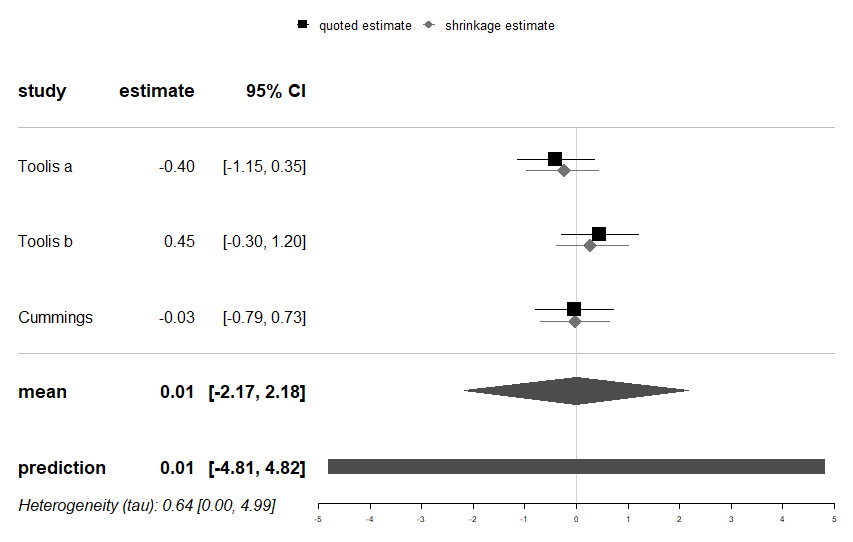


**Figure 40.** The Forest Plot in Athletic performance Open label


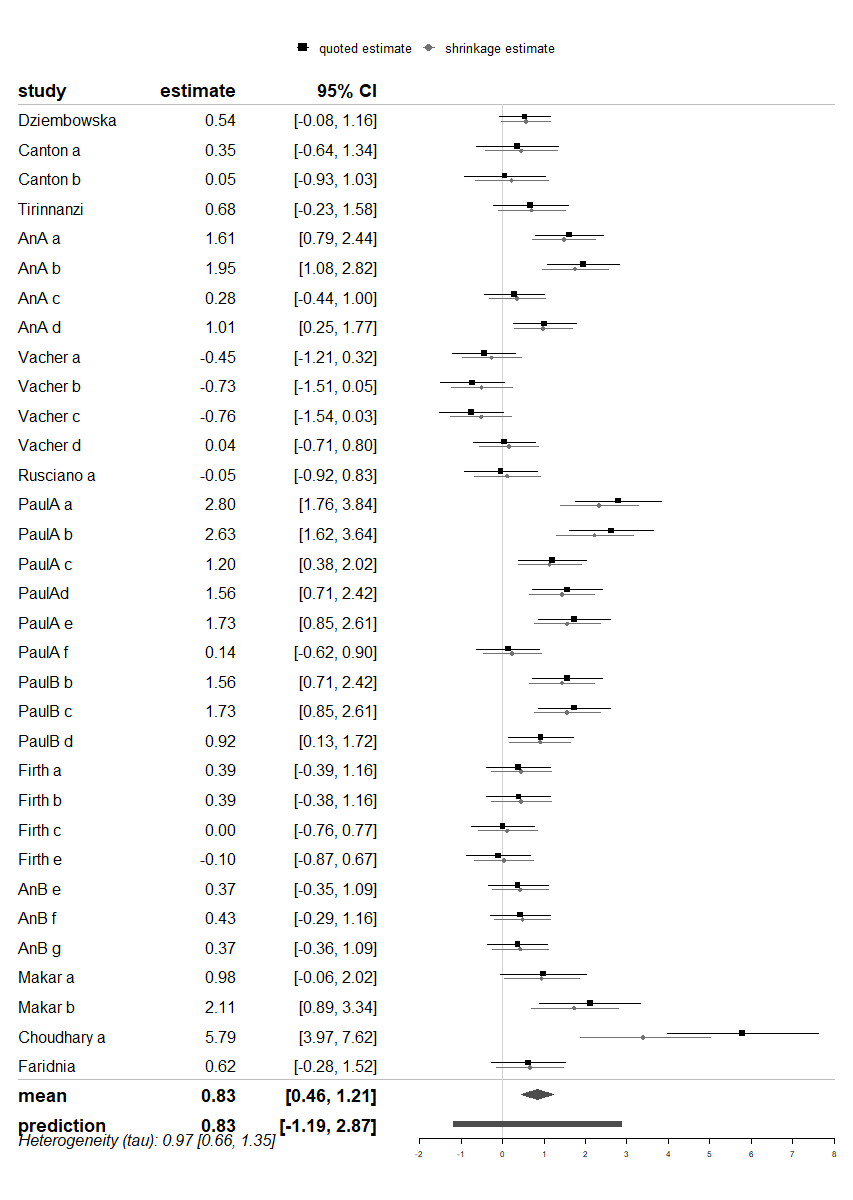


**Figure 41.** The Forest Plot in Mental health Adequate blinding


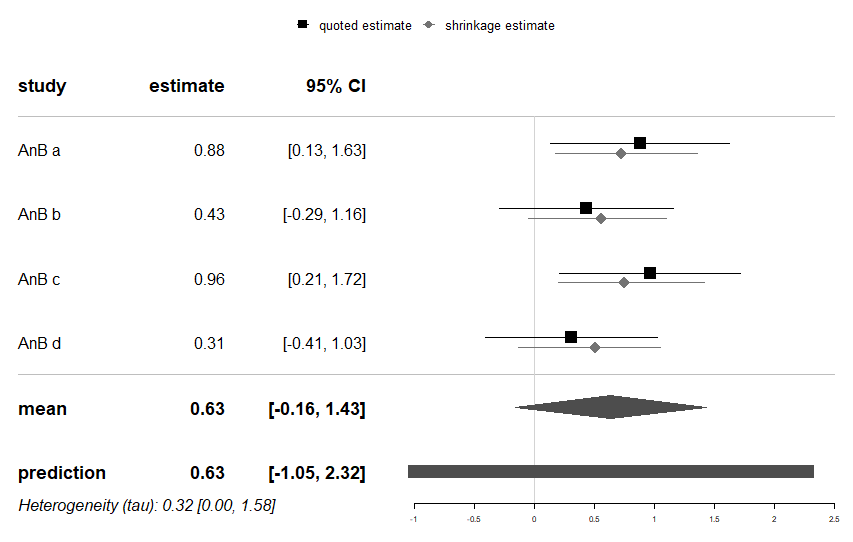


**Figure 42.** The Forest Plot in Mental health Open label


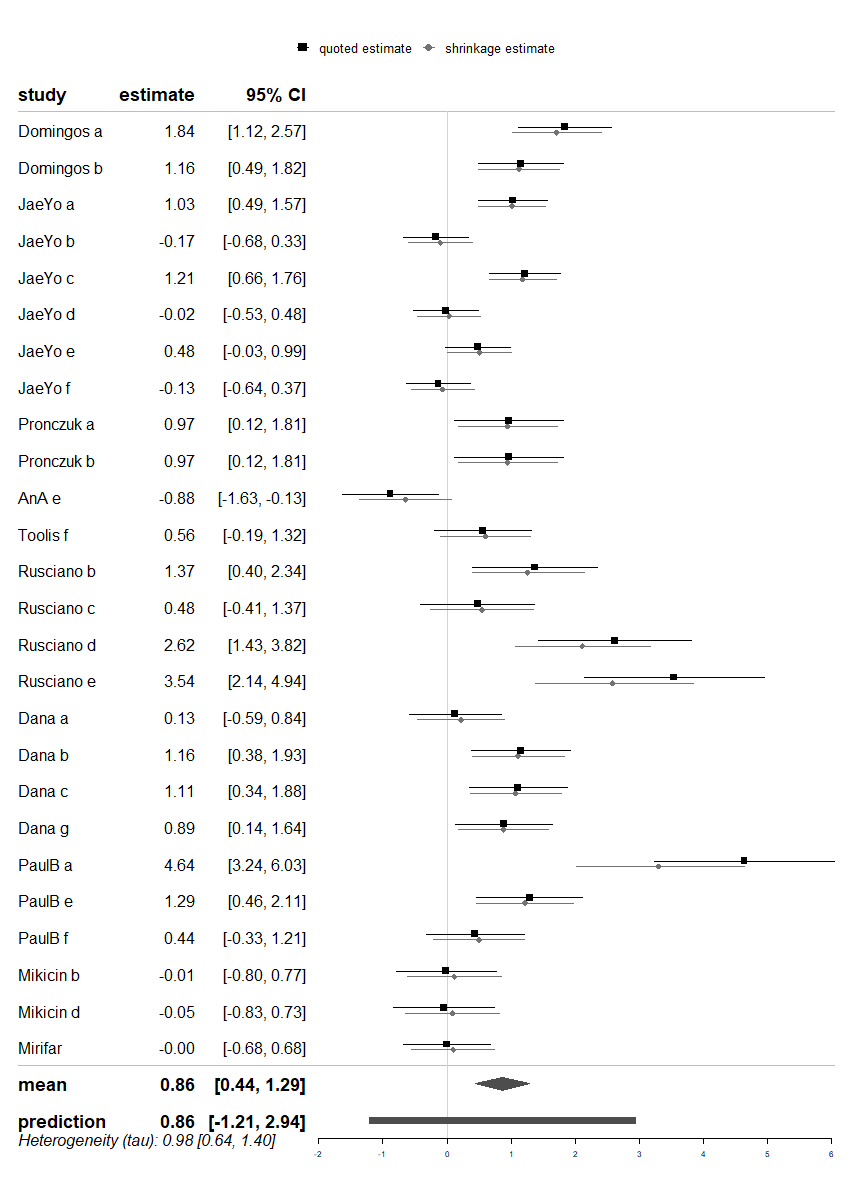


**Figure 43.** The Forest Plot in Cognitive performance Adequate blinding


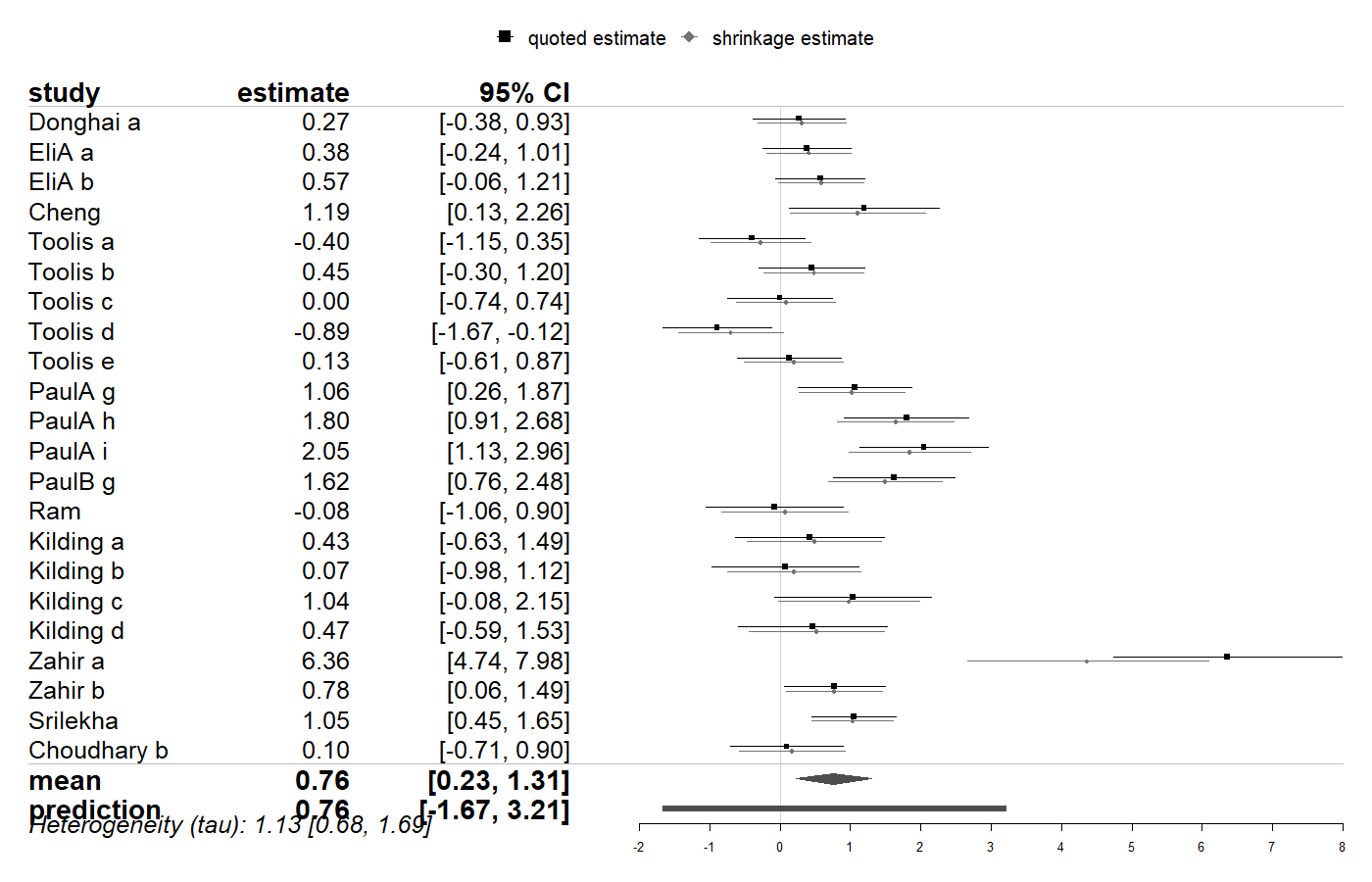


**Figure 44.** The Forest Plot in Athletic performance forest Elite


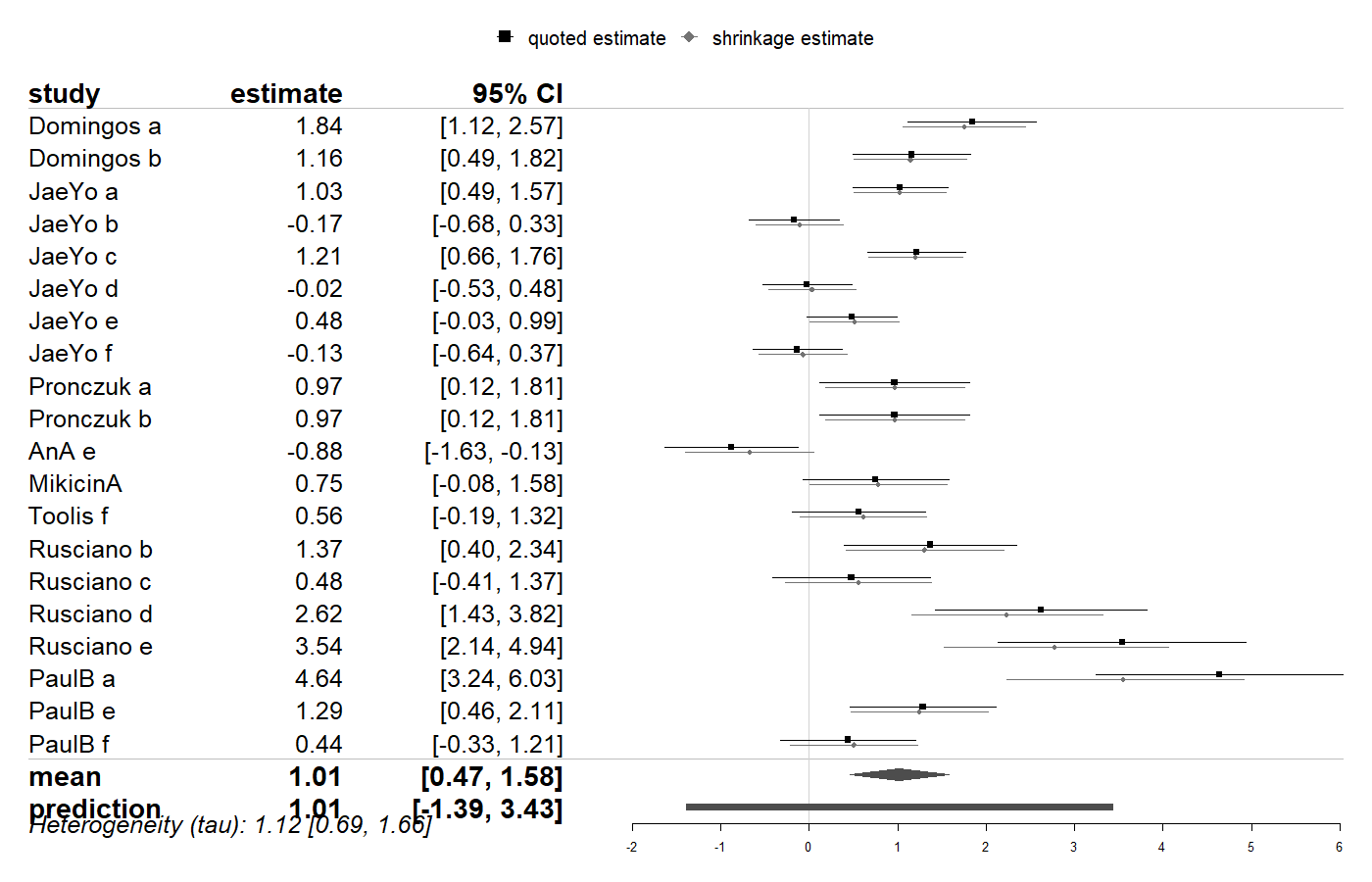


**Figure 45.** The Forest Plot in Cognitive performance forest Elite


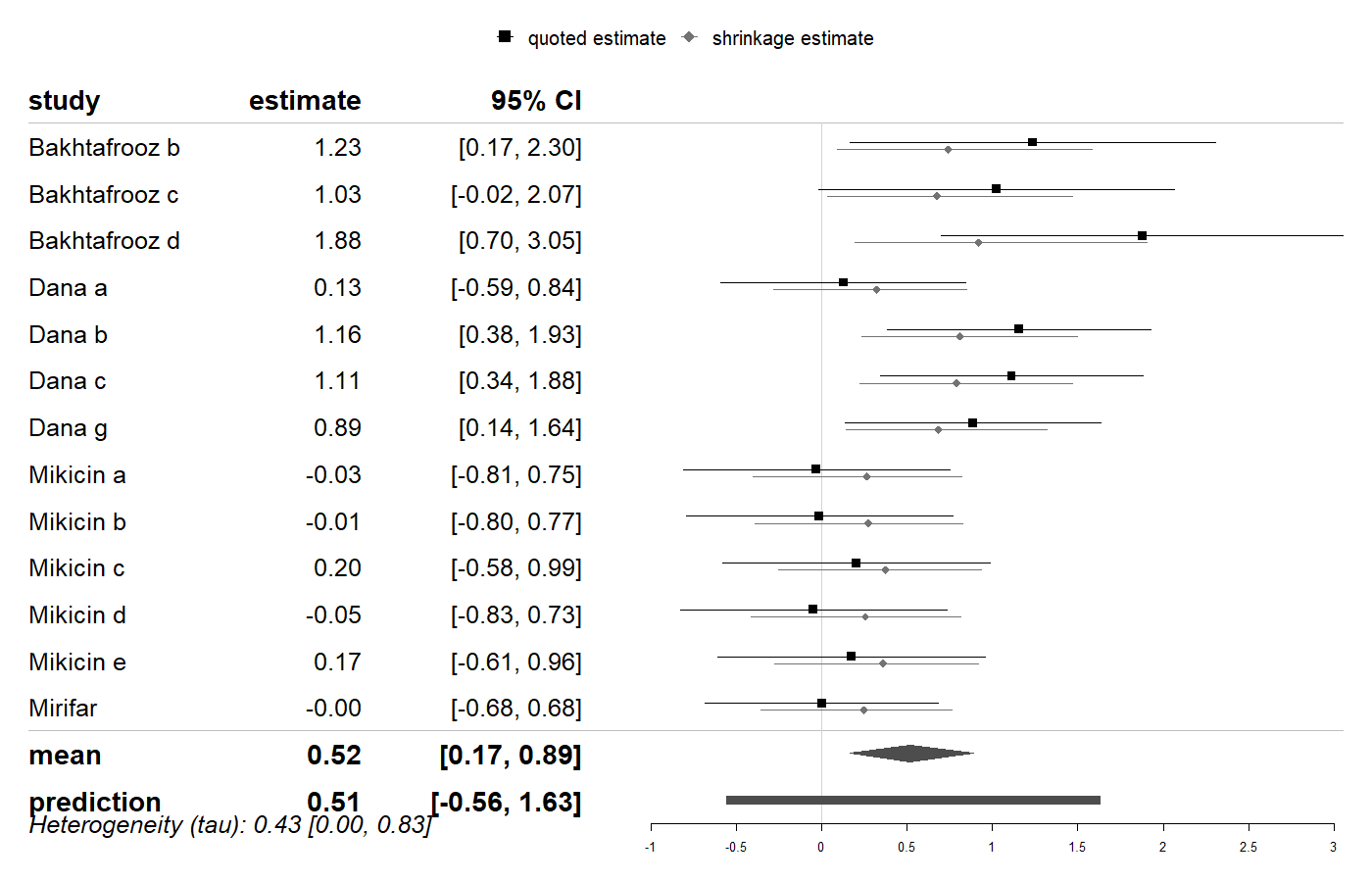


**Figure 45.** The Forest Plot in Cognitive performance forest NonElite


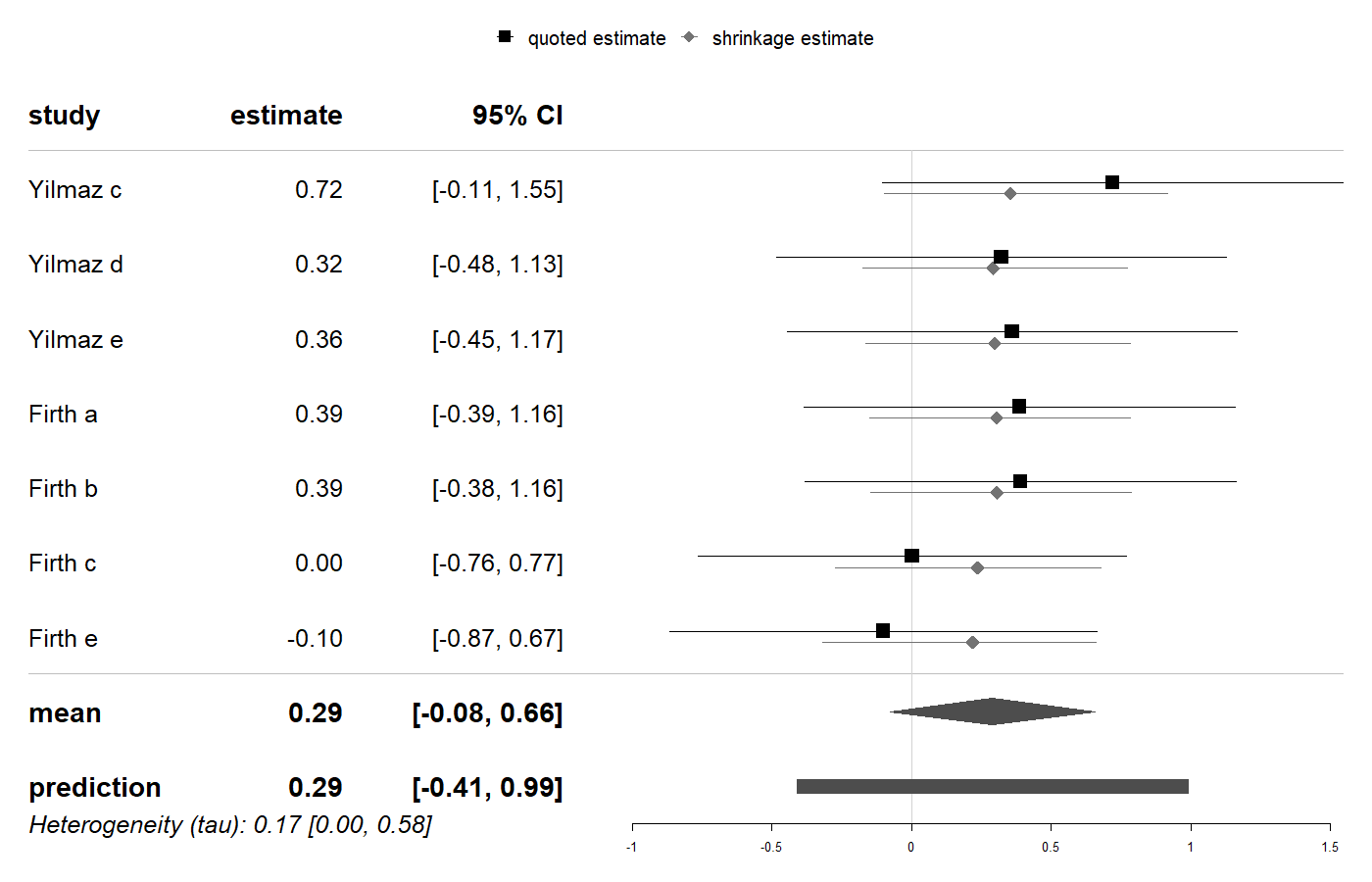


**Figure 45.** The Forest Plot in Mental health forest NonElite


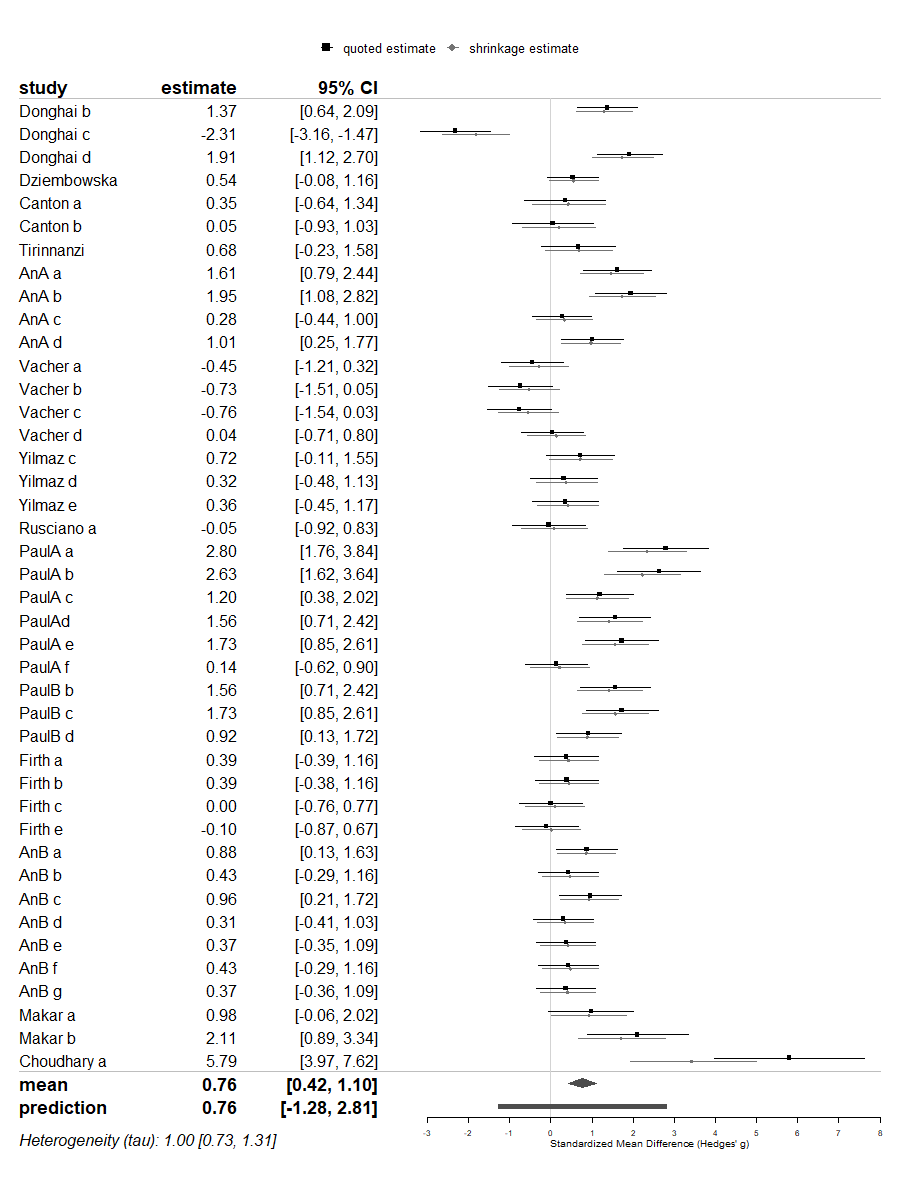


**Figure 46.** The Forest Plot in Mental Health Biofeedback


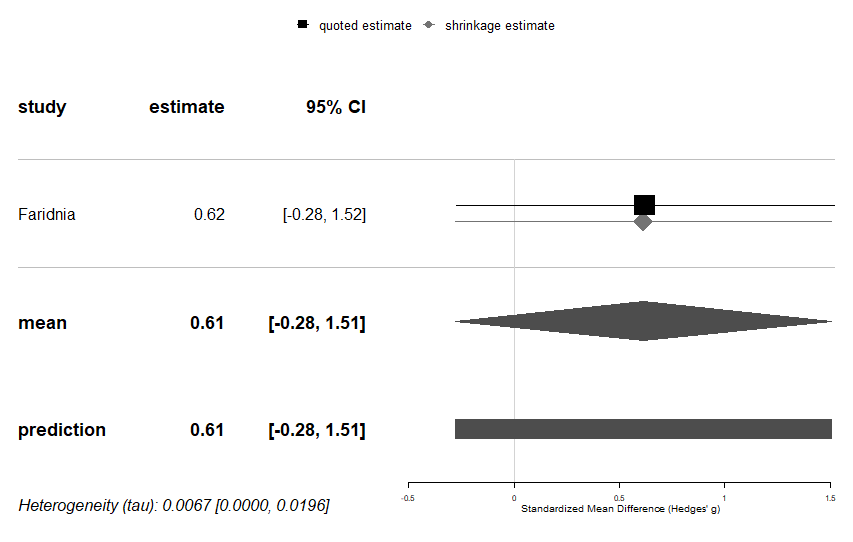


**Figure 47.** The Forest Plot in Mental Health Neurofeedback
