# Supplementary material for: The effects of biofeedback training on athletes’ mental health and performance: a systematic review and Bayesian meta-analysis
Source: Front Psychol. 2025 Oct 21;16:1662868. doi: 10.3389/fpsyg.2025.1662868 (PMC12583207; doi:10.3389/fpsyg.2025.1662868)
Supplement: Supplementary file 1 [file Data_Sheet_1.ZIP › Supplementary file S6 Sensitivity analysis chart.docx]

**Moderator analyses**


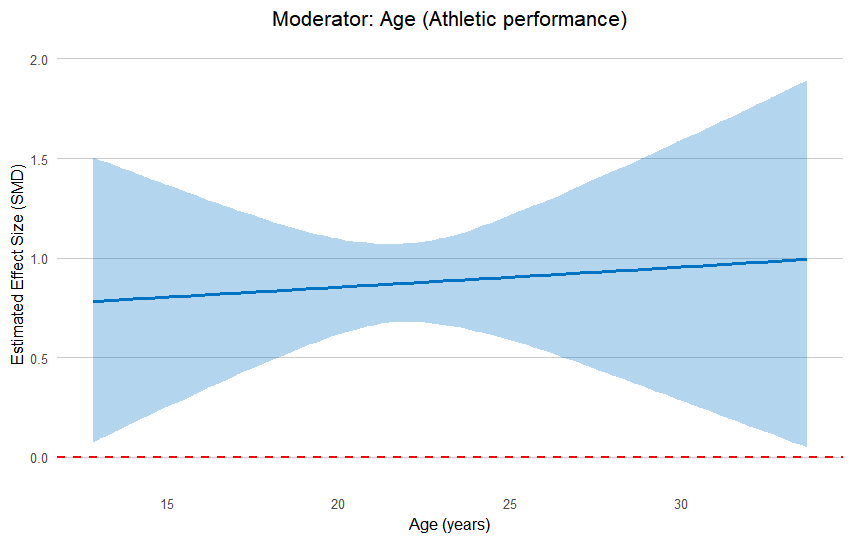


**Figure 1.Moderator analyses** Plot for Age Athletic Performance


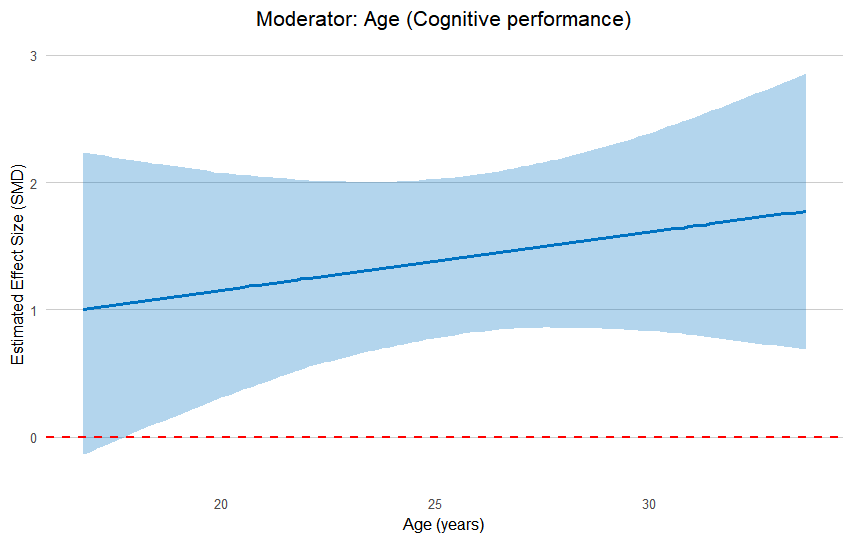


**Figure 2.** Moderator analyses Plot for Age Cognitive Performance


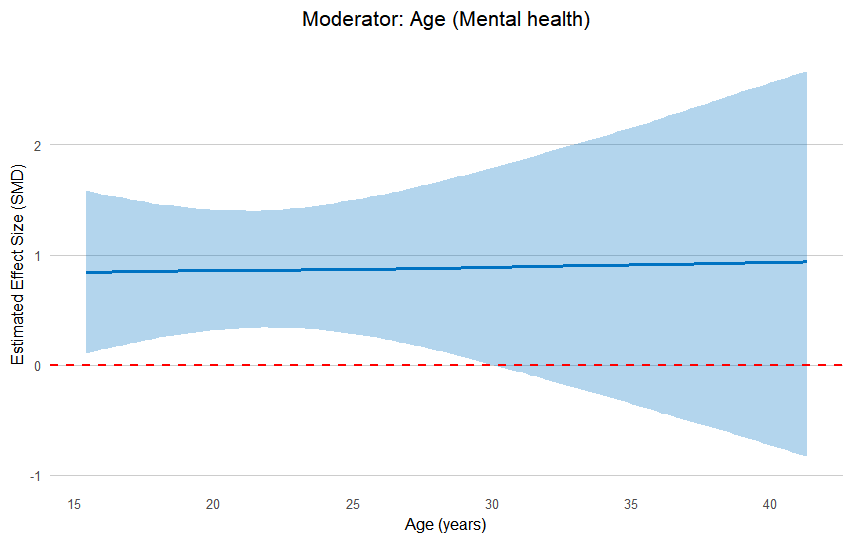
**Figure 3.** Moderator analyses Plot for Age Mental health


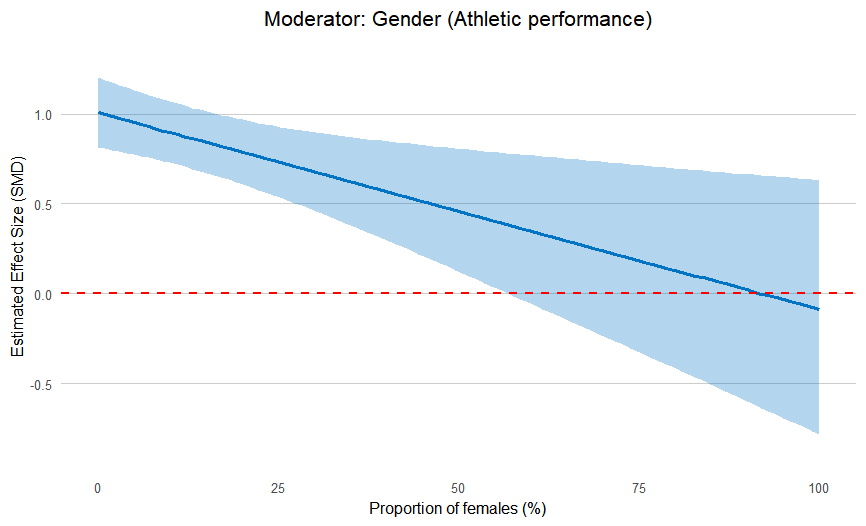


**Figure 4.** Moderator analyses Plot for Gender Athletic Performance

**
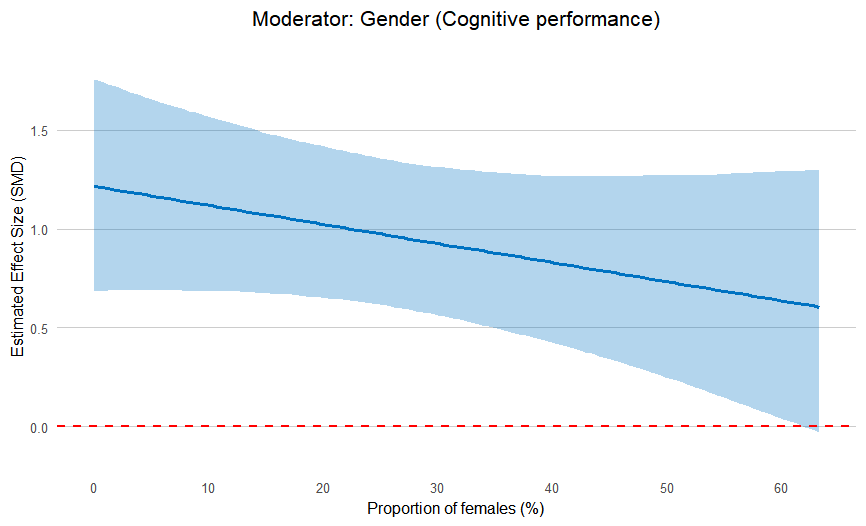
Figure 5.** Moderator analyses Plot for Gender Cognitive Performance


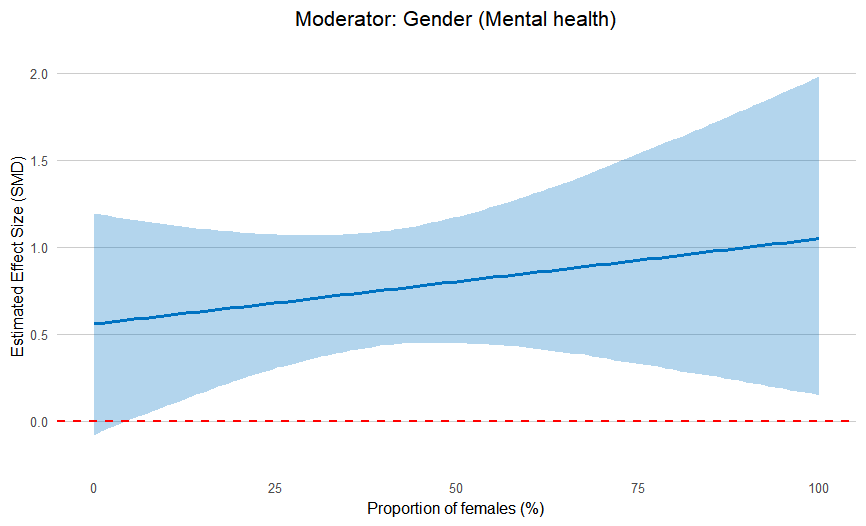


**Figure 6.** Moderator analyses Plot for Gender Mental health
