# Supplementary material for: The effects of biofeedback training on athletes’ mental health and performance: a systematic review and Bayesian meta-analysis
Source: Front Psychol. 2025 Oct 21;16:1662868. doi: 10.3389/fpsyg.2025.1662868 (PMC12583207; doi:10.3389/fpsyg.2025.1662868)
Supplement: Supplementary file 1 [file Data_Sheet_1.ZIP › Supplementary file S7 Funnel plots.docx]

**Subgroup Analysis based on Intervention Type (Biofeedback vs Neurofeedback)**


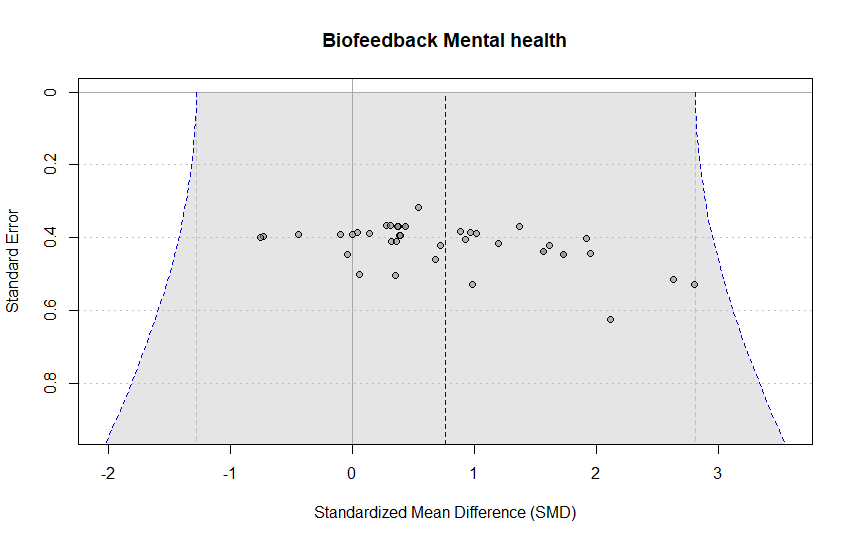


**Figure 1.** The Funnel Plot in Athletic Performance Biofeedback


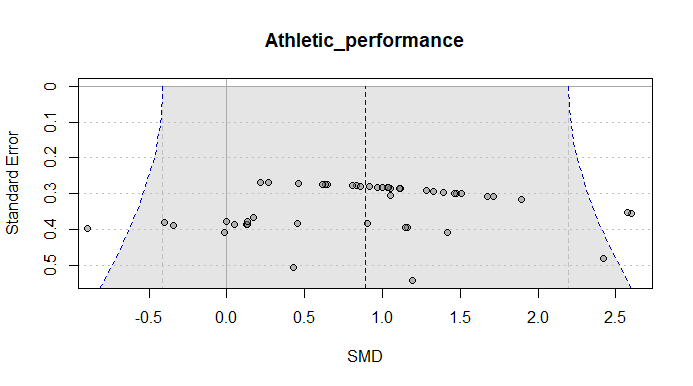


**Figure 2.** The Funnel Plot in Athletic Performance Neurofeedback


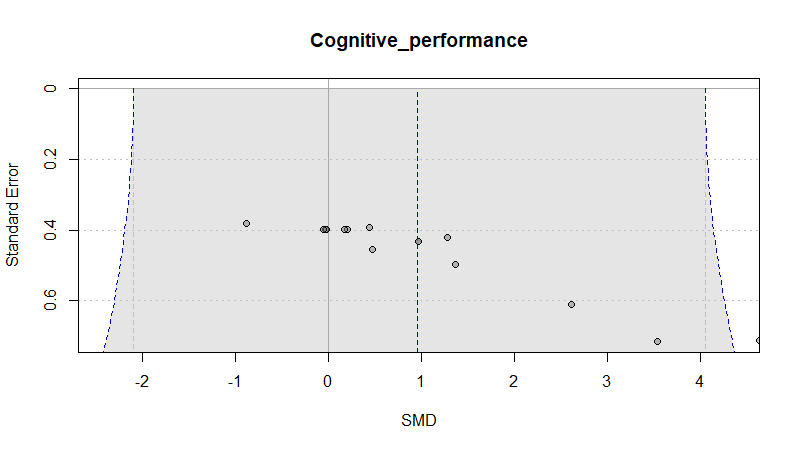


**Figure 3.** The Funnel Plot in Cognitive Performance Biofeedback


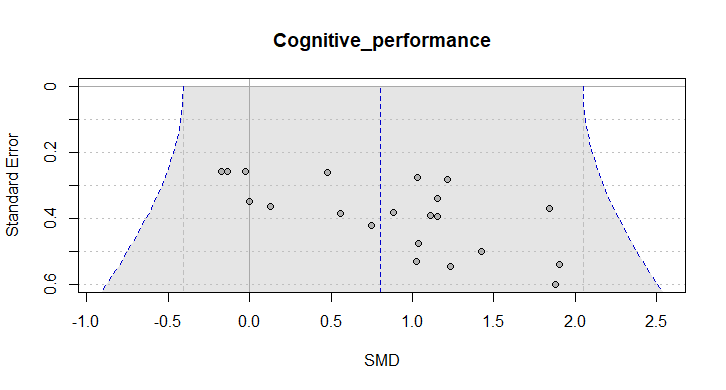


**Figure 4.** The Funnel Plot in Cognitive Performance Neurofeedback

**Subgroup analysis based on specific psychological and Performance outcomes**


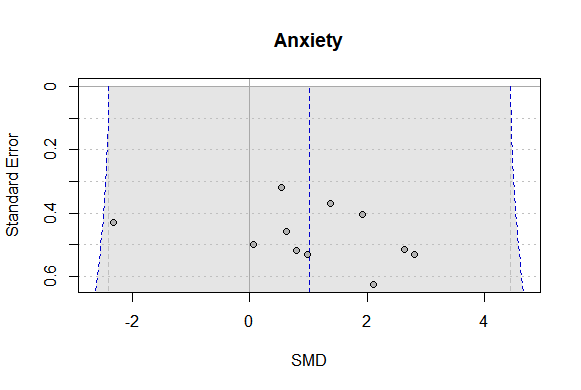


**Figure 5.** The Funnel Plot in Anxiety Biofeedback


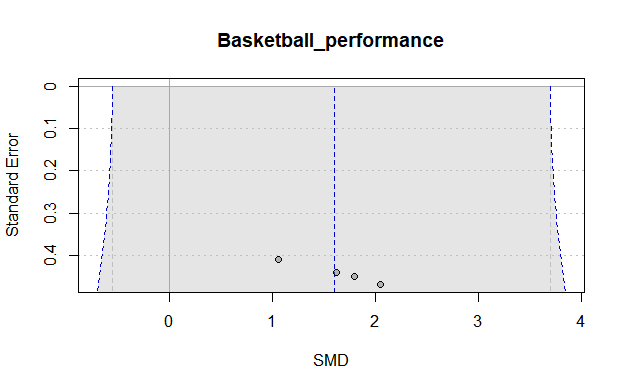


**Figure 6.** The Funnel Plot in Basketball Performance Biofeedback


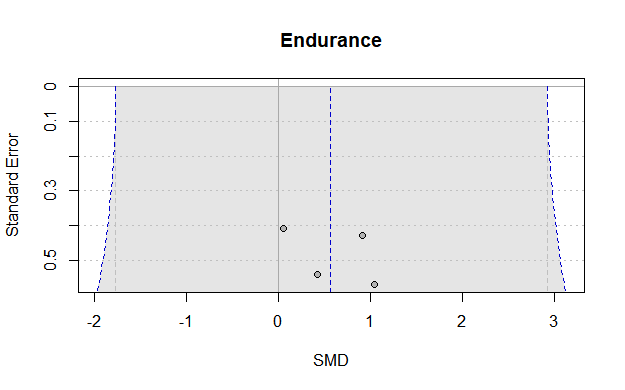


**Figure 7.** The Funnel Plot in Endurance Biofeedback


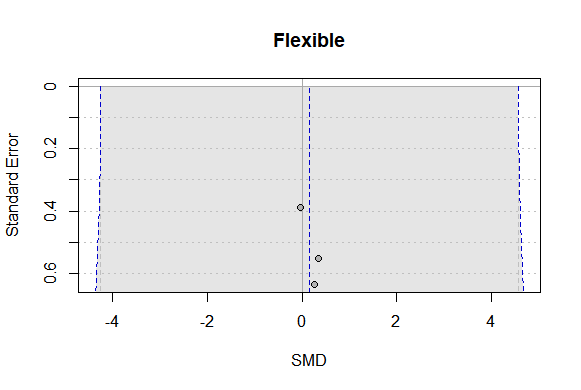


**Figure 8.** The Funnel Plot in Flexible Biofeedback


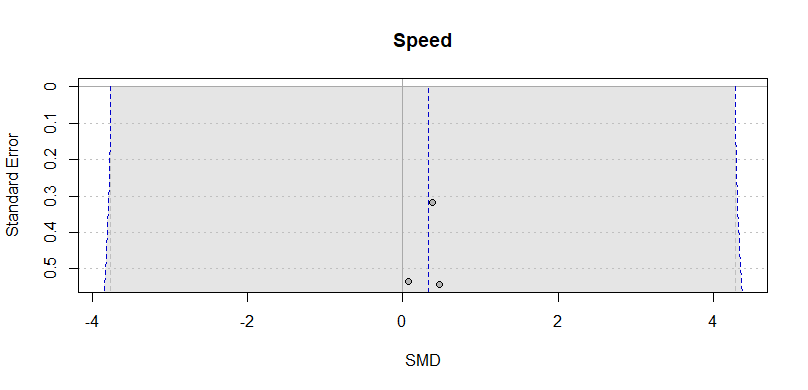


**Figure 9.** The Funnel Plot in Speed Biofeedback


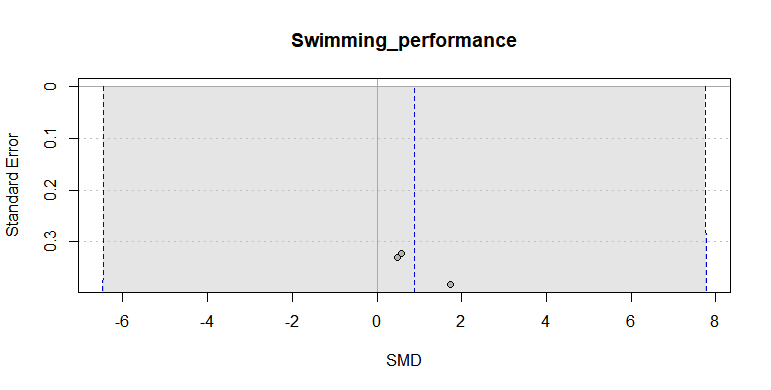


**Figure 10.** The Funnel Plot in Swimming Performance Biofeedback


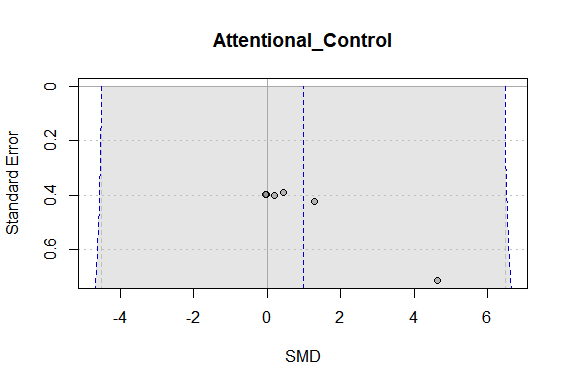


**Figure 11.** The Funnel Plot in Attentional Control Biofeedback


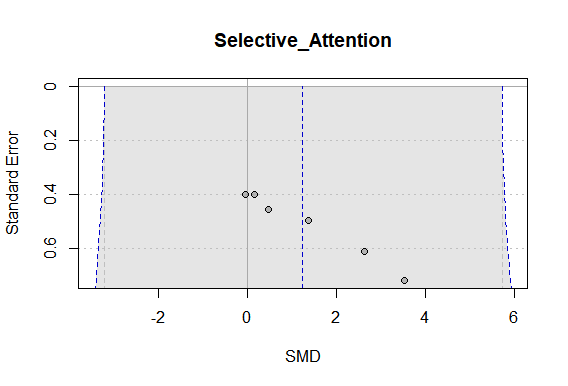


**Figure 12.** The Funnel Plot in Selective Attention Biofeedback


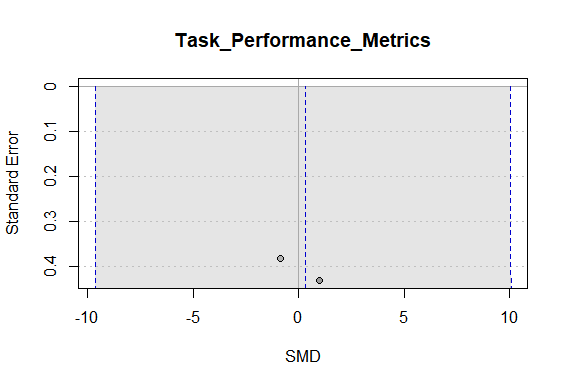


**Figure 13.** The Funnel Plot in Task Performance Metrics Biofeedback


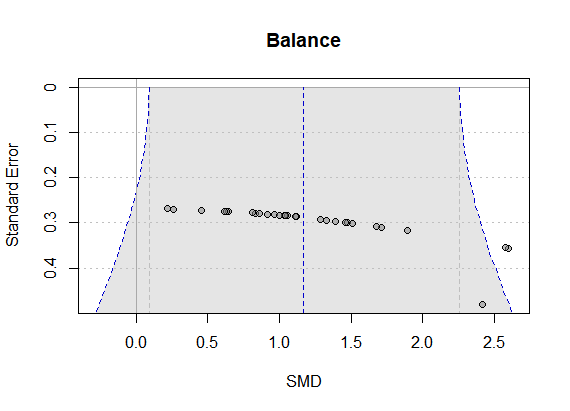


**Figure 14.** The Funnel Plot in Task Balance Neurofeedback


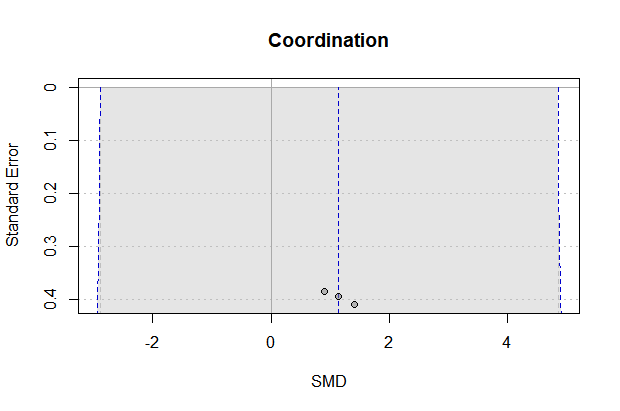


**Figure 15.** The Funnel Plot in Coordination Neurofeedback


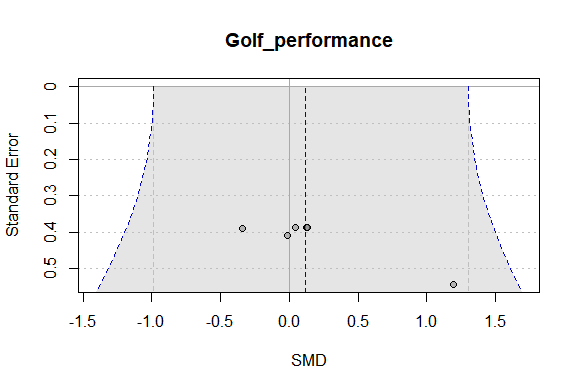


**Figure 16.** The Funnel Plot in Golf Performance Neurofeedback


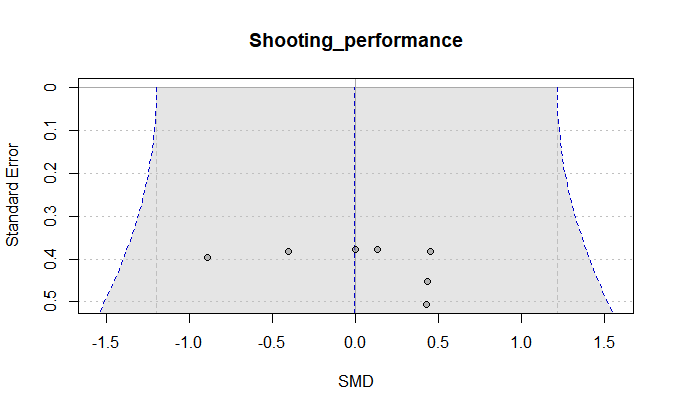


**Figure 17.** The Funnel Plot in Shooting_Performance Neurofeedback


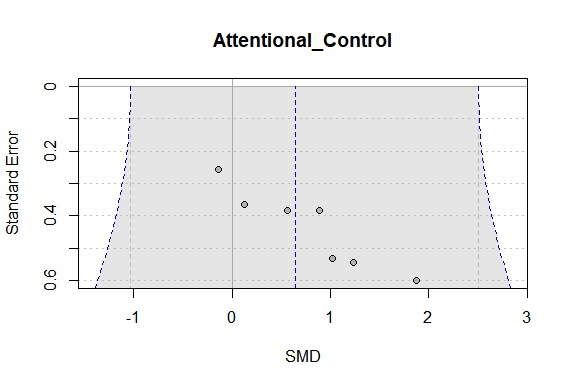


**Figure 18.** The Funnel Plot in Attentional_Control Neurofeedback

**
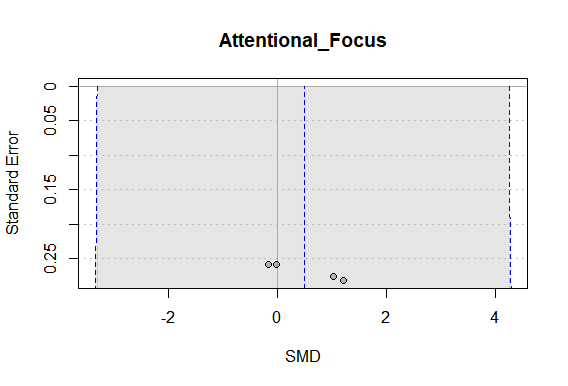
**

**Figure 19.** The Funnel Plot in Attentional_Focus Neurofeedback

**Subgroup Analysis Based on Biofeedback Dose**


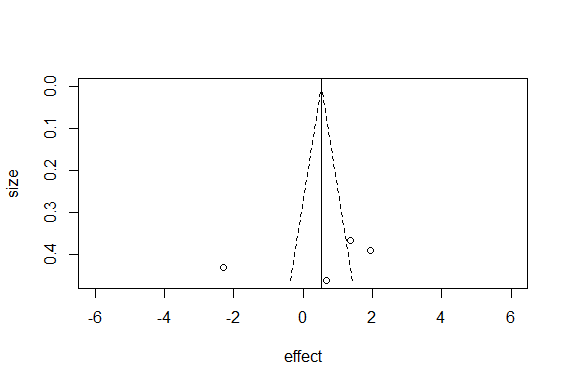


**Figure 20.** The Funnel Plot in More Than 10 Weeks Mental Health


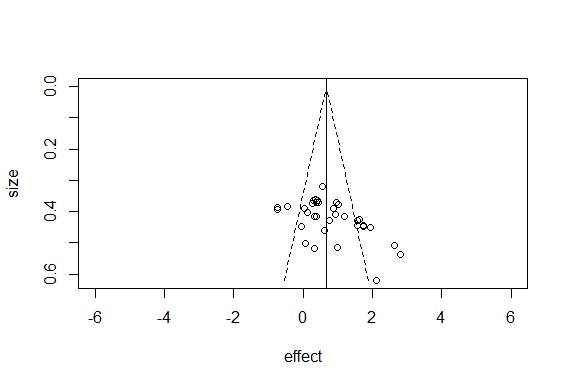


**Figure 21.** The Funnel Plot in 5 Weeks or Less Mental Health


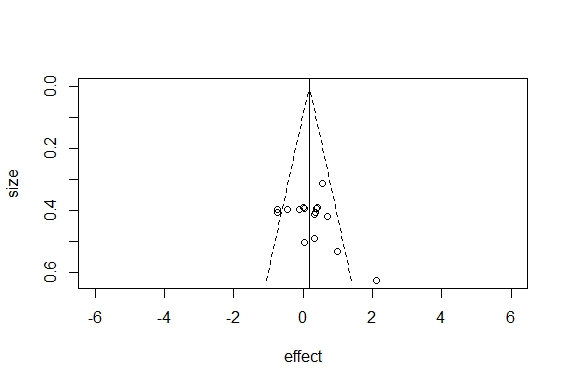


**Figure 22.** The Funnel Plot in 20 Minutes or Less Mental Health


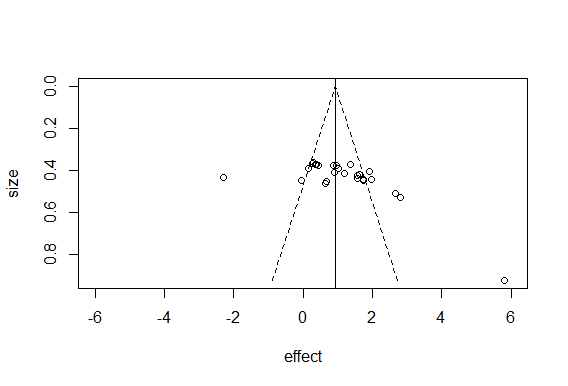


**Figure 23.** The Funnel Plot in 21-40 Minute Mental Health


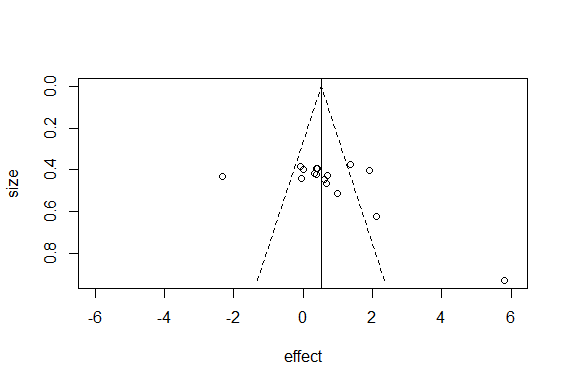


**Figure 24.** The Funnel Plot in 3 Times or Less Mental Health


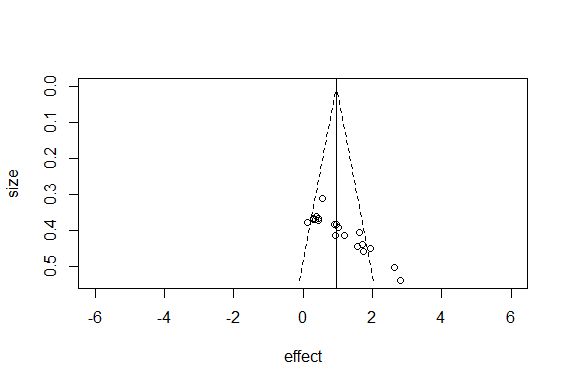


**Figure 25.** The Funnel Plot in 4-5 Times Mental Health


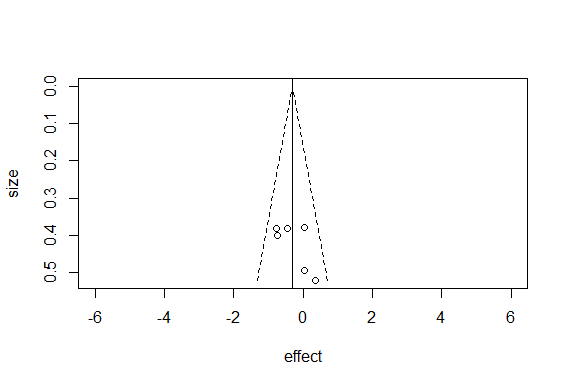


**Figure 26.** The Funnel Plot in 6-7 Times Mental Health


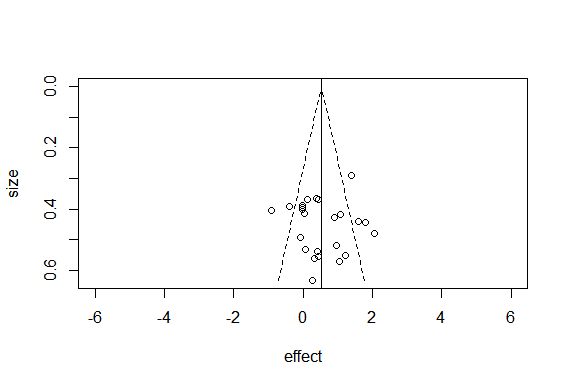


**Figure 27.** The Funnel Plot in 5 Weeks or Less Athletic Performance


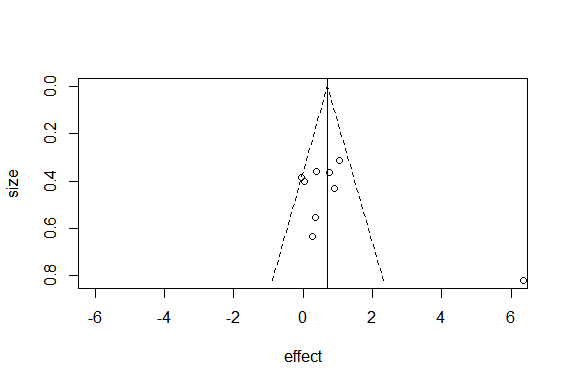


**Figure 28.** The Funnel Plot in 20 Minutes or Less Athletic Performance


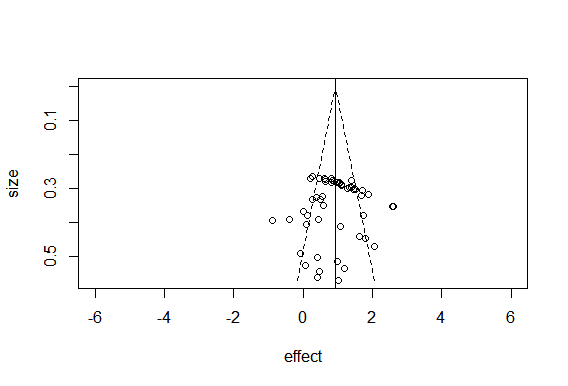


**Figure 29.** The Funnel Plot in 21-40 Minutes Athletic Performance


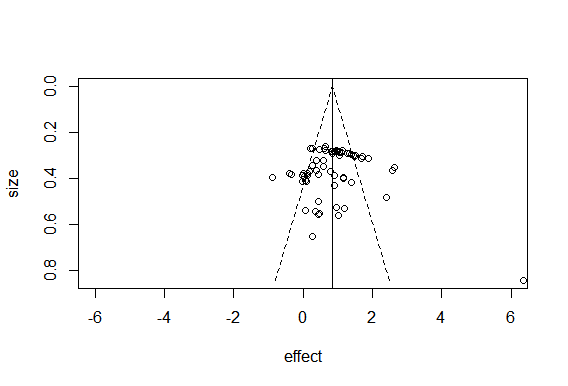


**Figure 30.** The Funnel Plot in 3 Times or Less Athletic Performance


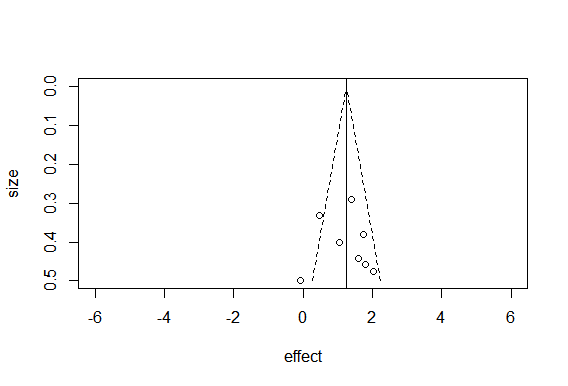


**Figure 31.** The Funnel Plot in 4-5 Times Athletic Performance


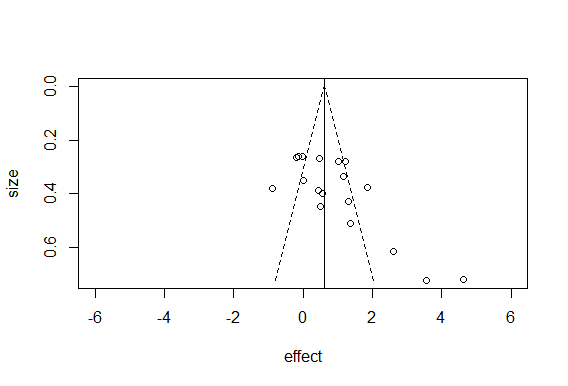


**Figure 32.** The Funnel Plot in 5 Weeks or Less Cognitive Performance


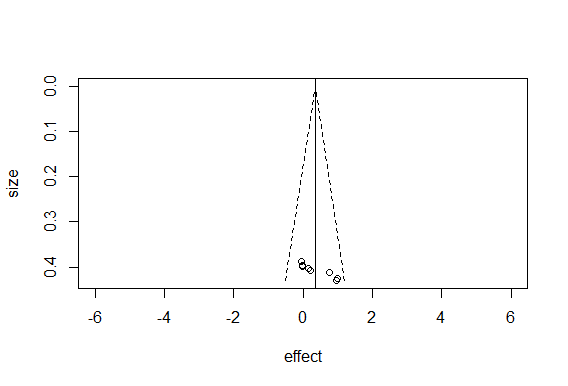


**Figure 33.** The Funnel Plot in More than 10 weeks Cognitive Performance


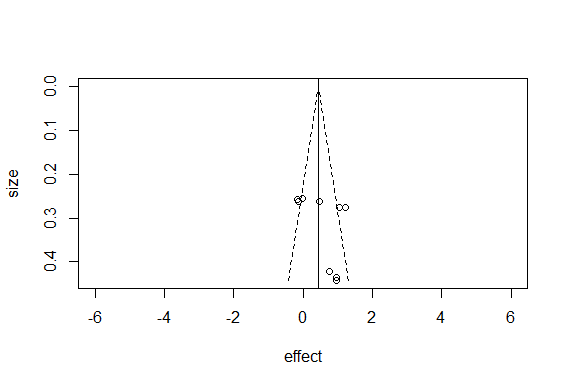


**Figure 34.** The Funnel Plot in 20 Minutes or Less Cognitive Performance


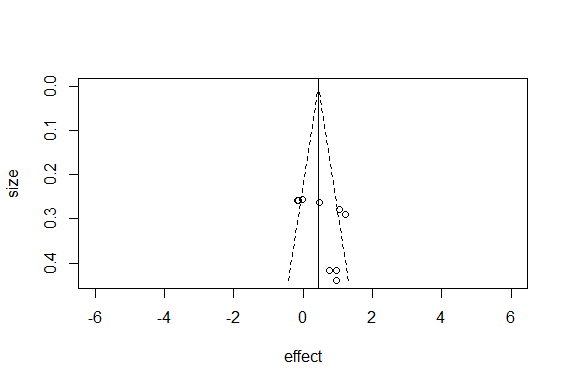


**Figure 35.** The Funnel Plot in 21-40 Minutes Cognitive Performance


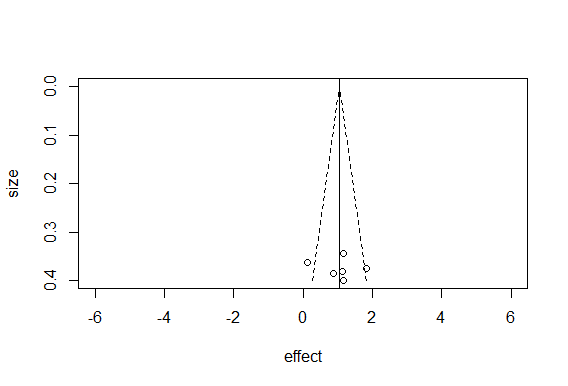


**Figure 36.** The Funnel Plot in 41-60 Minutes Cognitive Performance


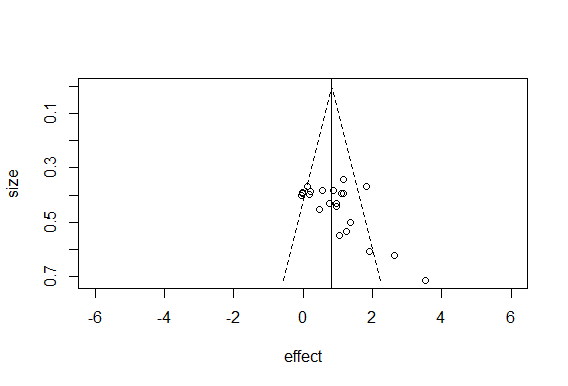


**Figure 37.** The Funnel Plot in 3 Times or Less Cognitive Performance


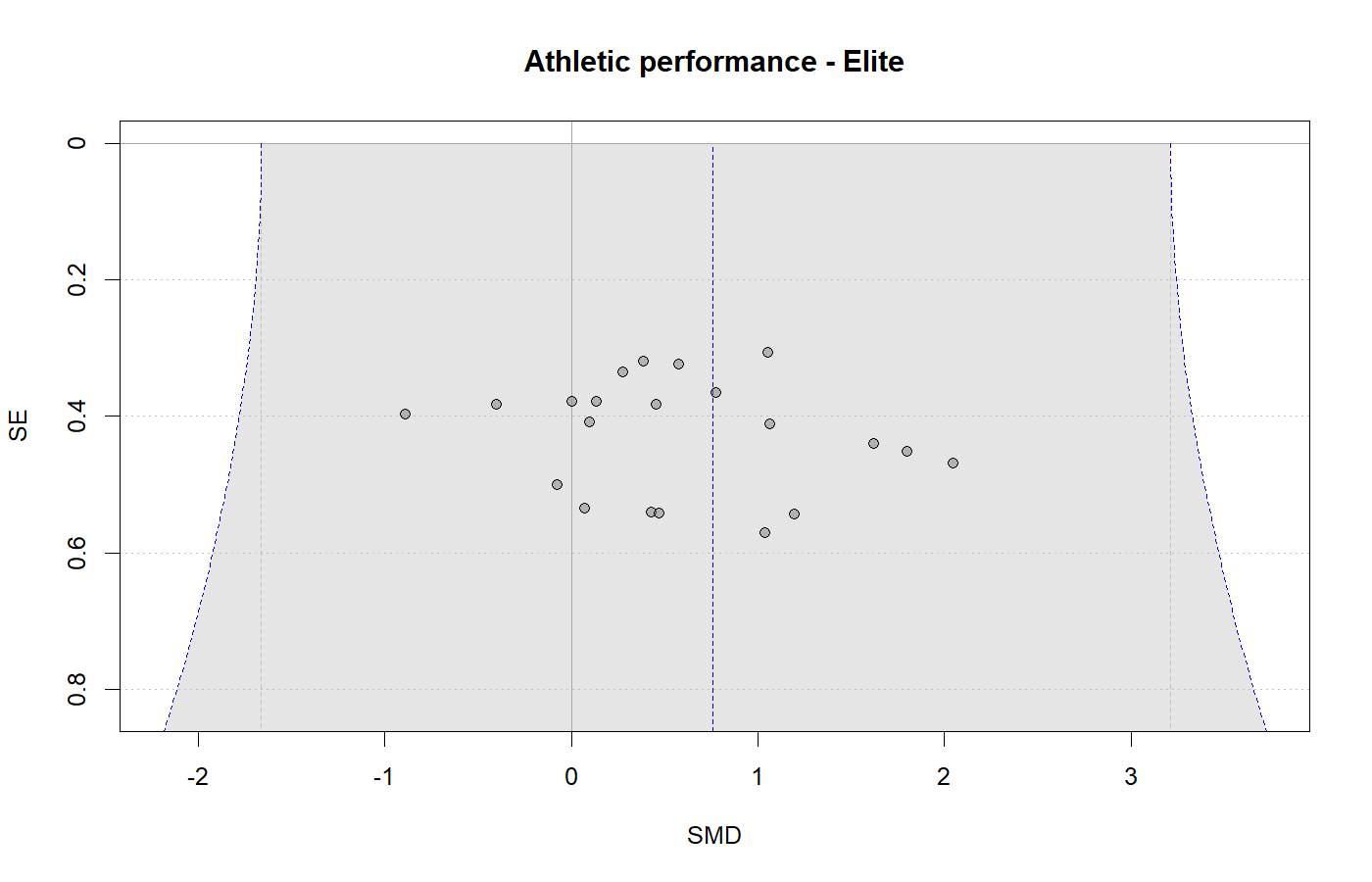


**Figure 38.** The Funnel Plot in Athletic performance funnel Elite


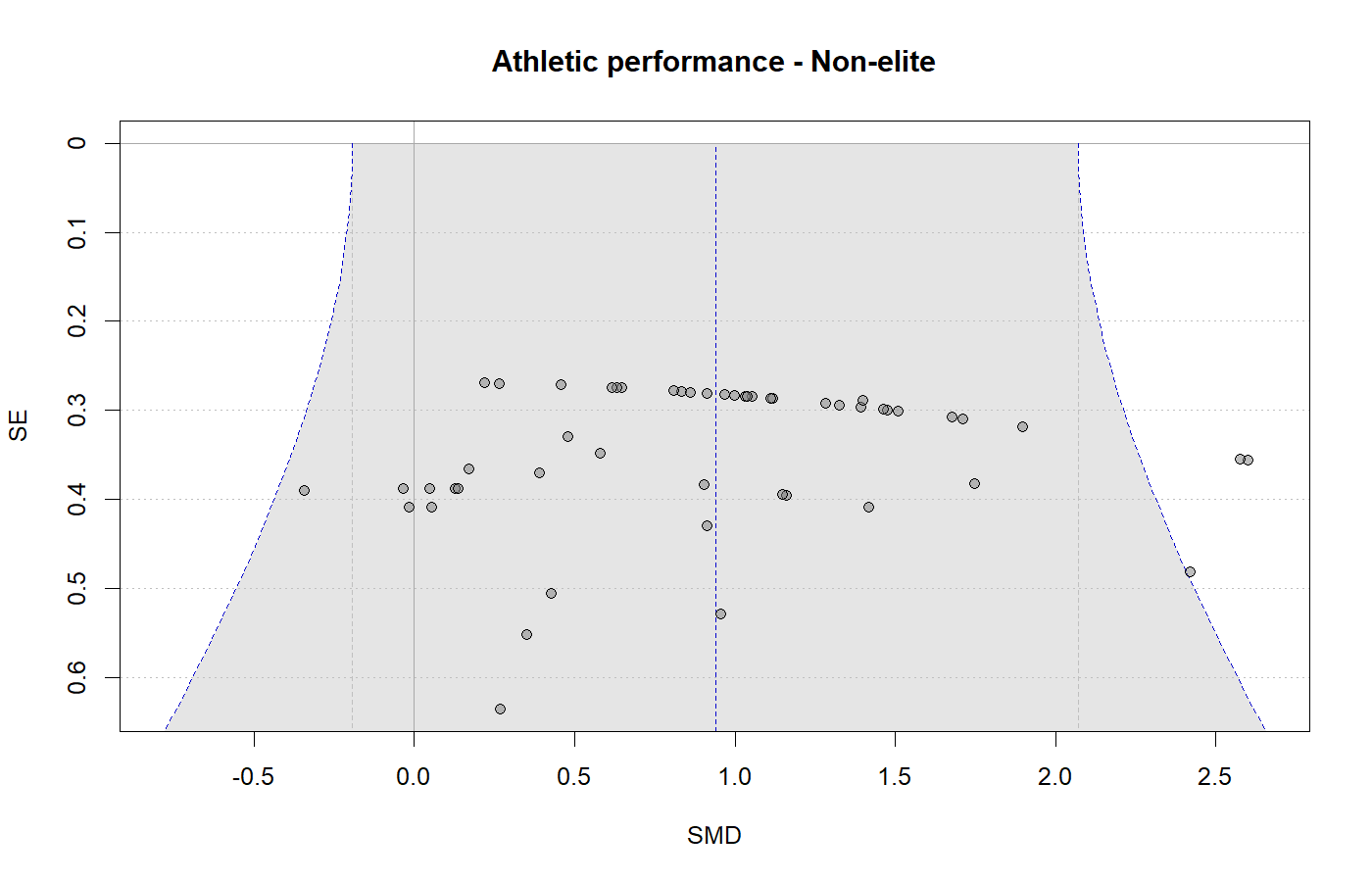


**Figure 39.** The Funnel Plot in Athletic performance funnel NonElite


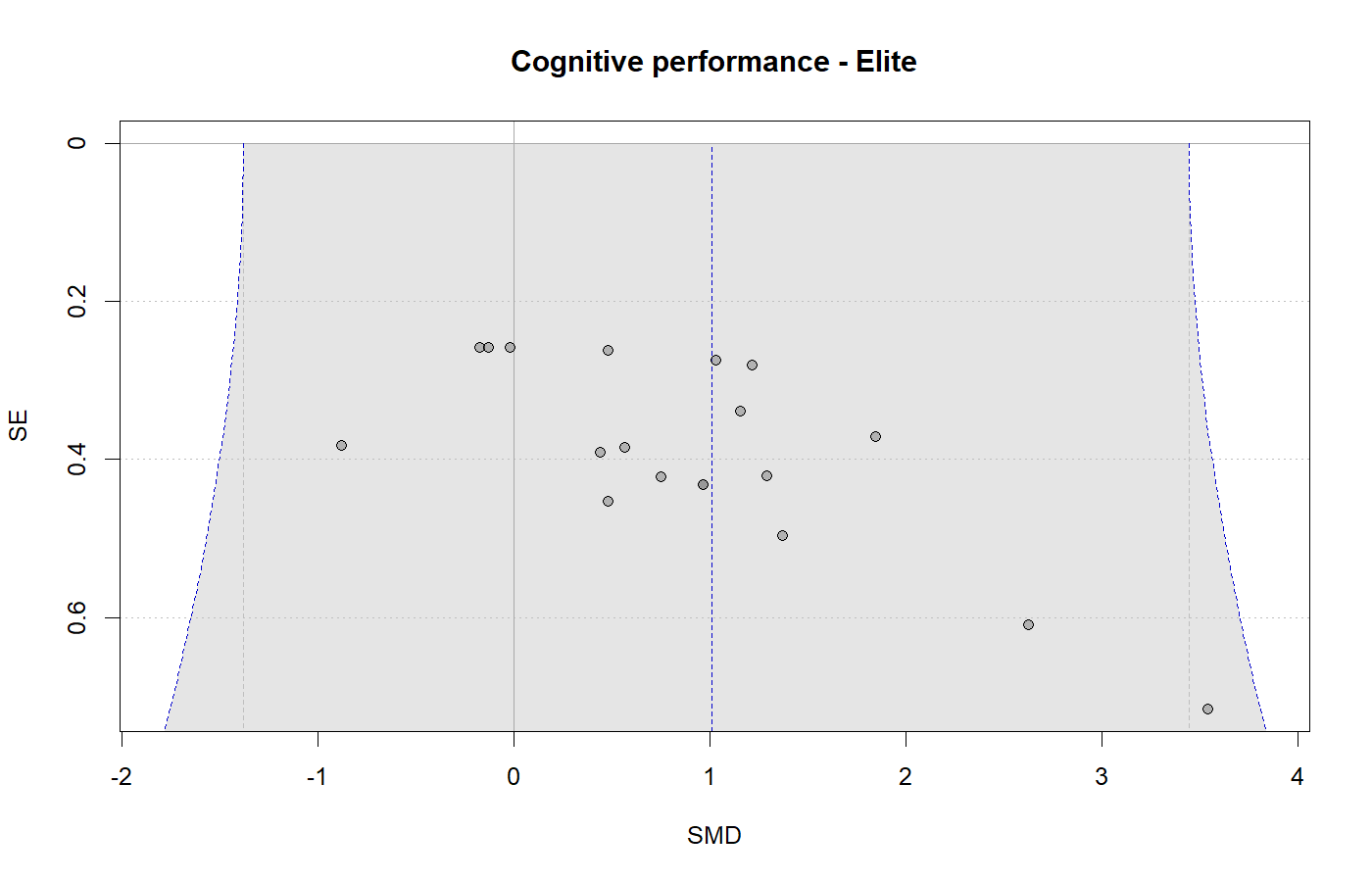


**Figure 40.** The Funnel Plot in Cognitive performance funnel Elite


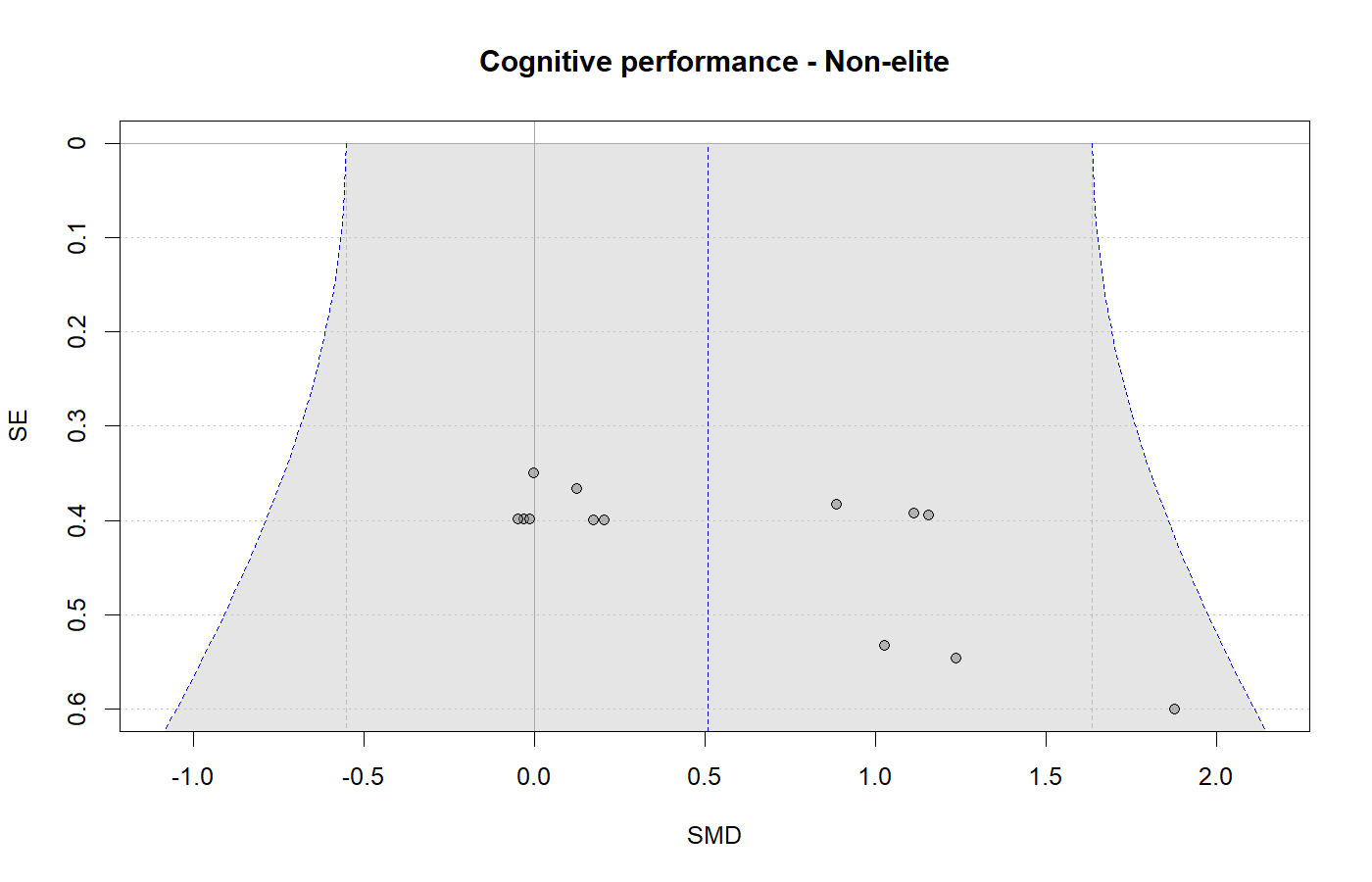


**Figure 41.** The Funnel Plot in Cognitive performance funnel NonElite


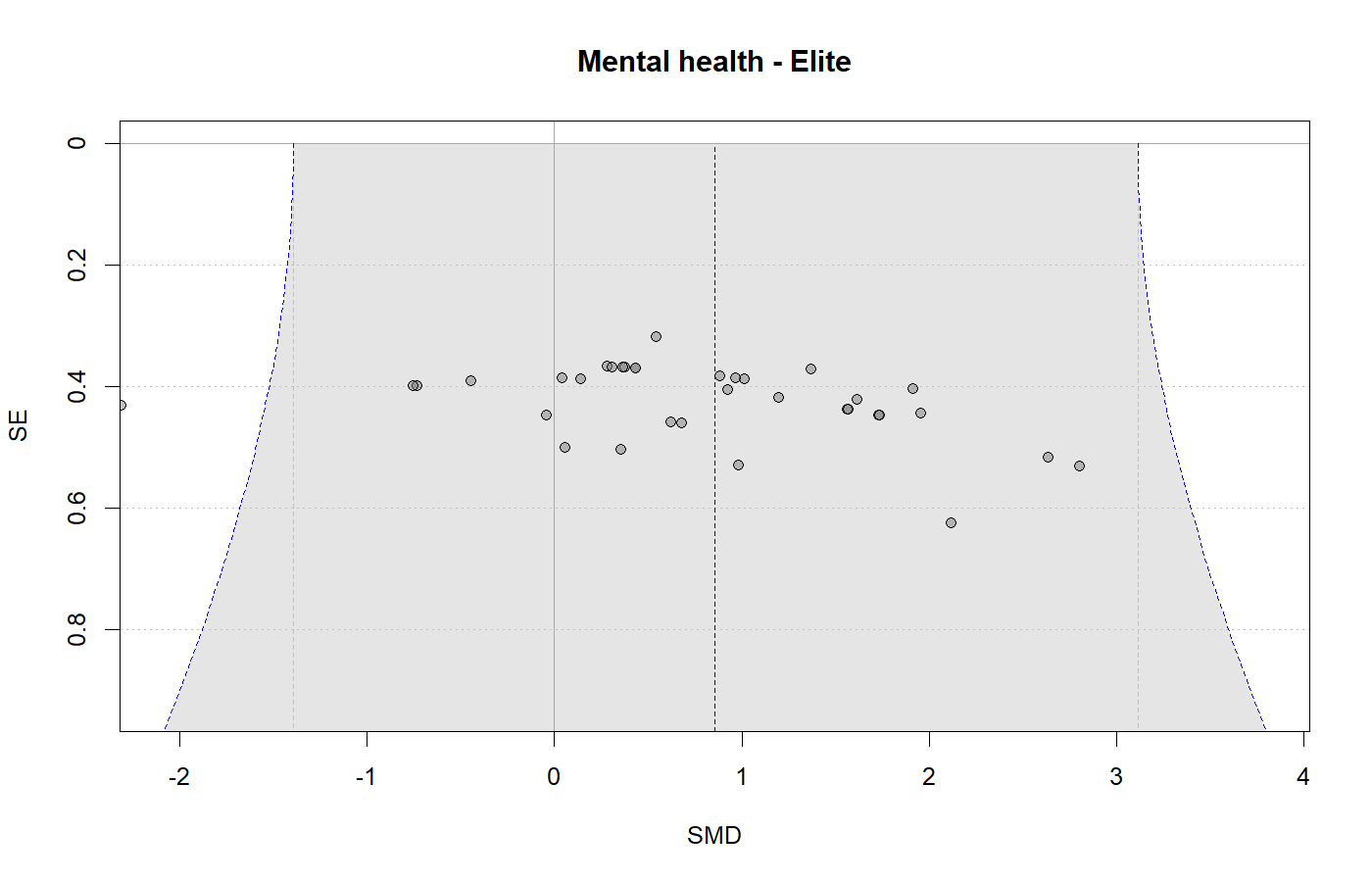


**Figure 42.** The Funnel Plot in Mental health funnel Elite


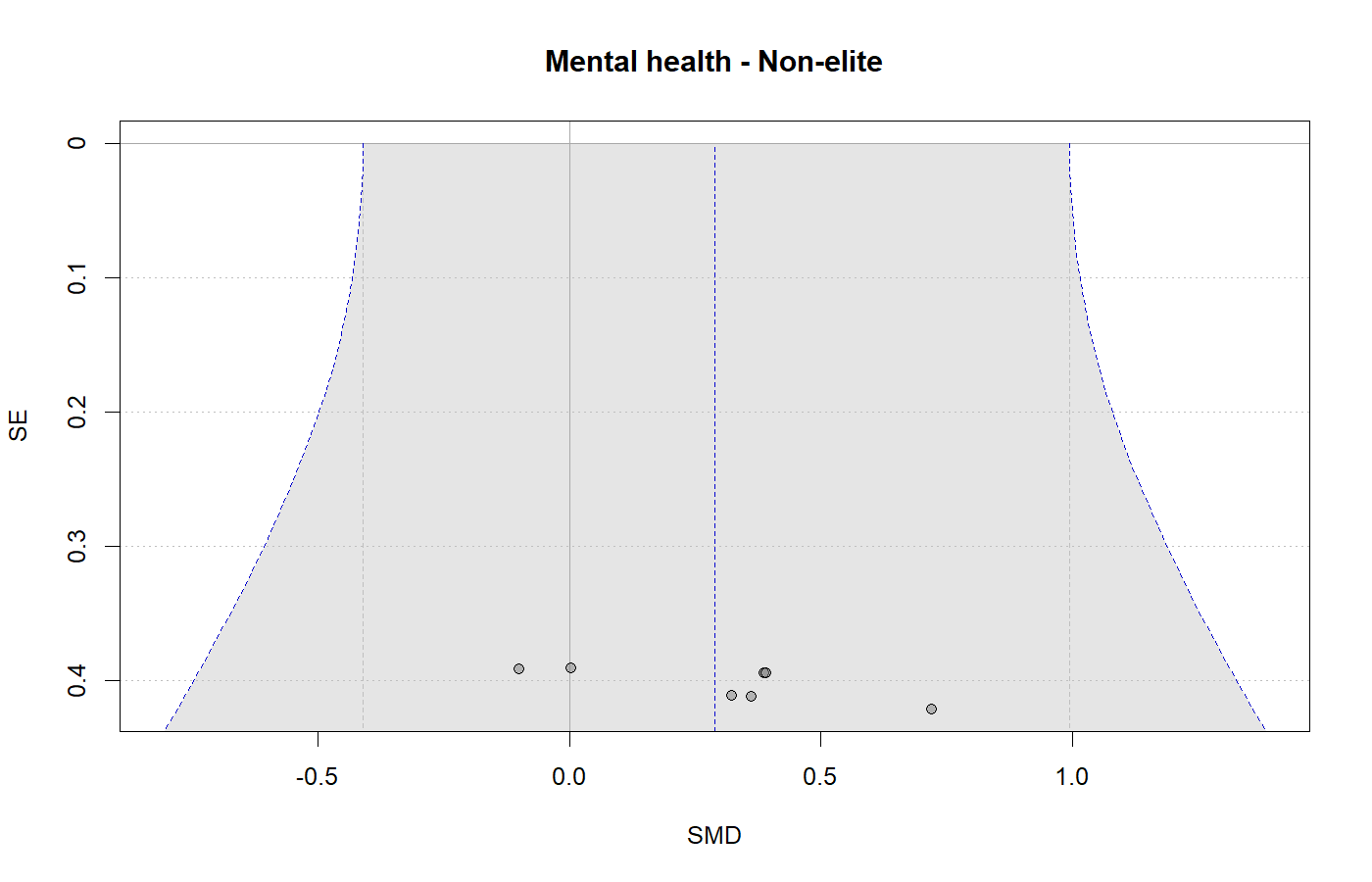


**Figure 43.** The Funnel Plot in Mental health funnel NonElite


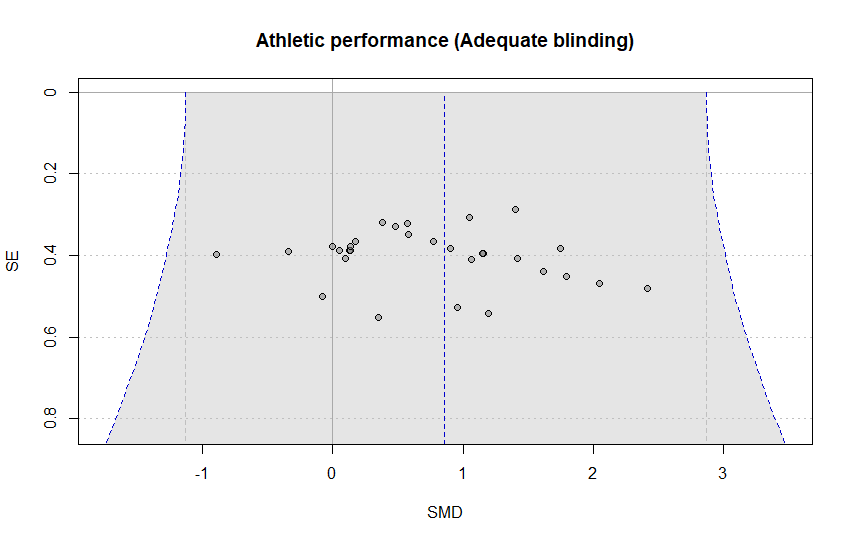


**Figure 44.** The Funnel Plot in Athletic performance Adequate blinding


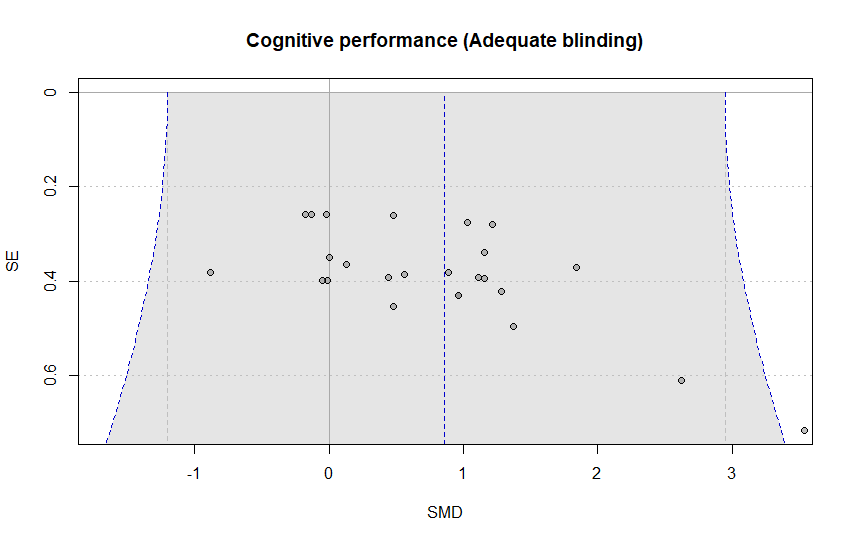


**Figure 45.** The Funnel Plot in Cognitive performance Adequate blinding


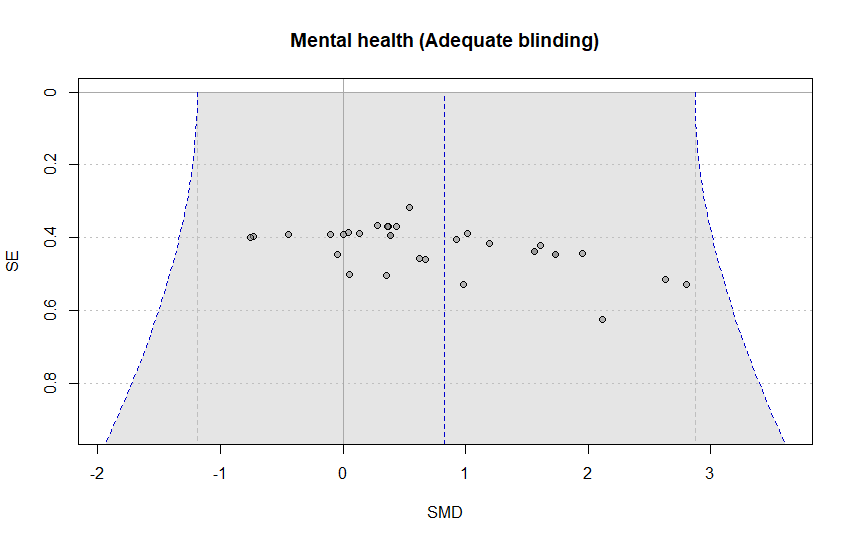


**Figure 46.** The Funnel Plot in Mental health Adequate blinding


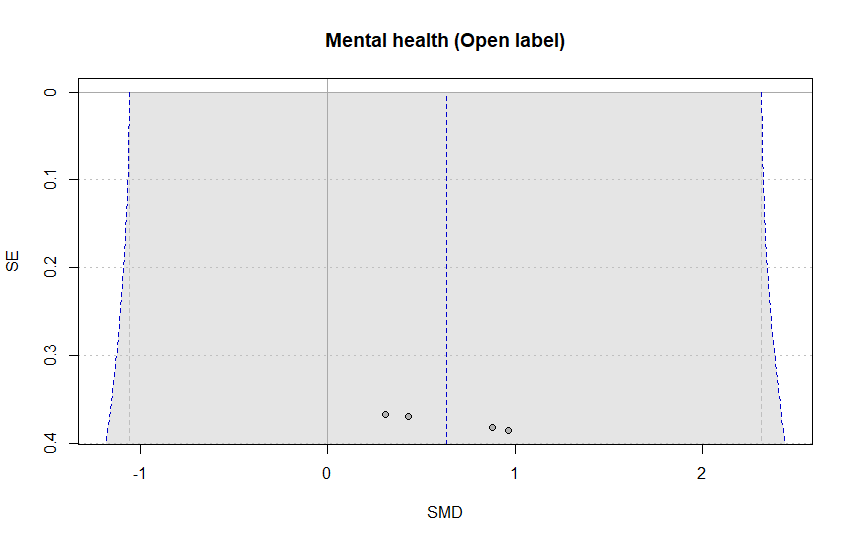


**Figure 47.** The Funnel Plot in Mental health Open label


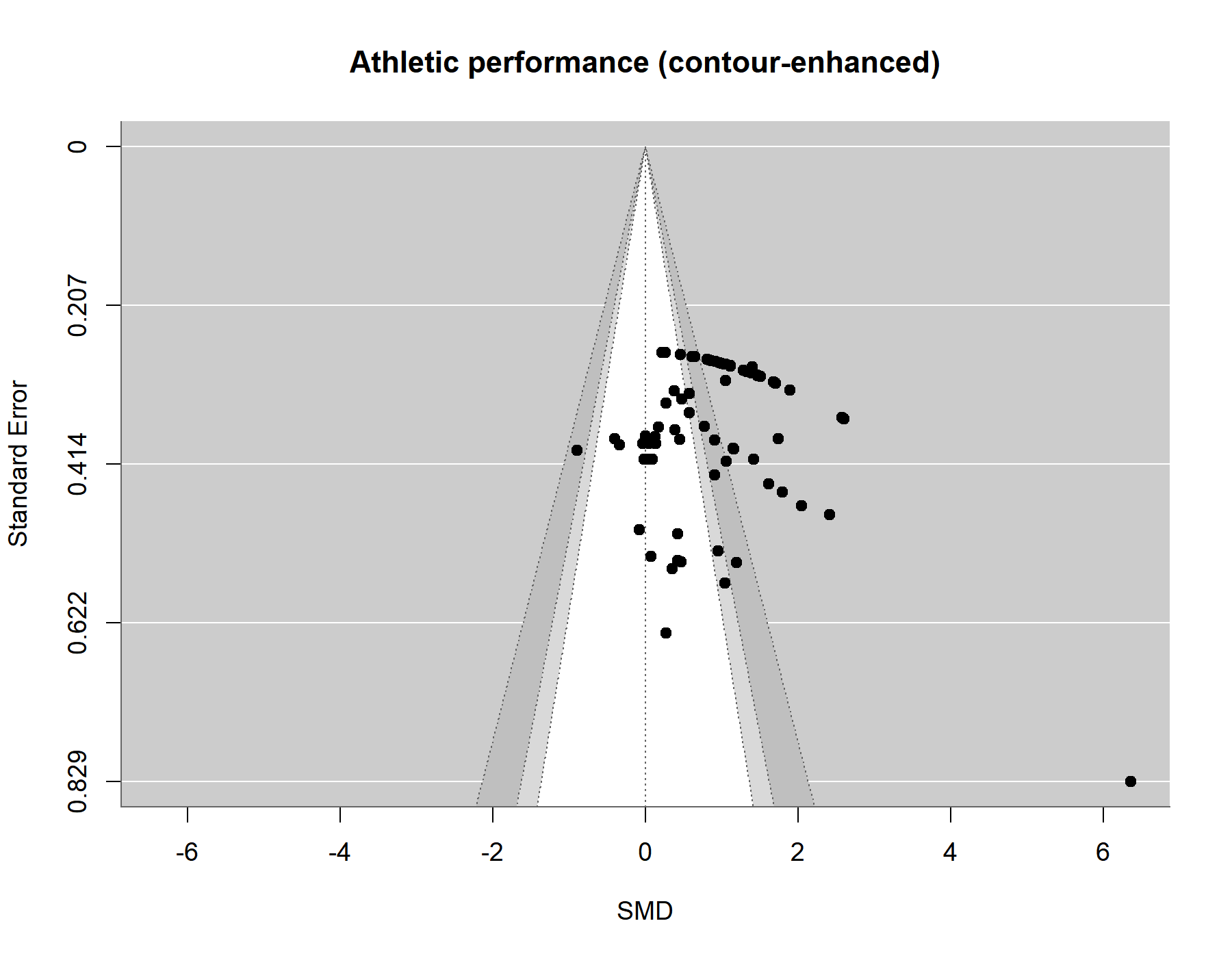


**Figure 48.** The Funnel Plot in Cognitive performance (contour-enhanced)


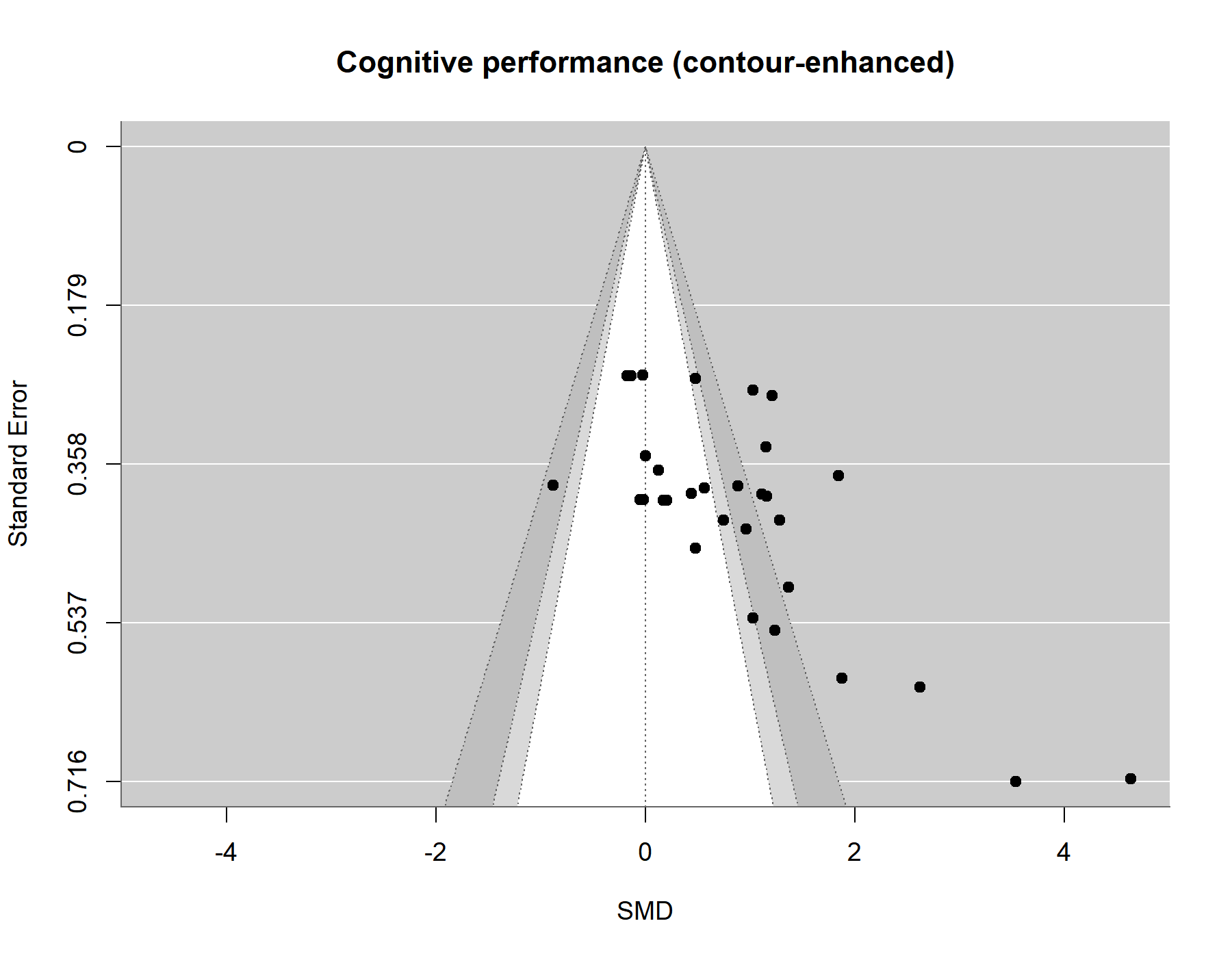
 **Figure 49.** The Funnel Plot in Cognitive performance (contour-enhanced)


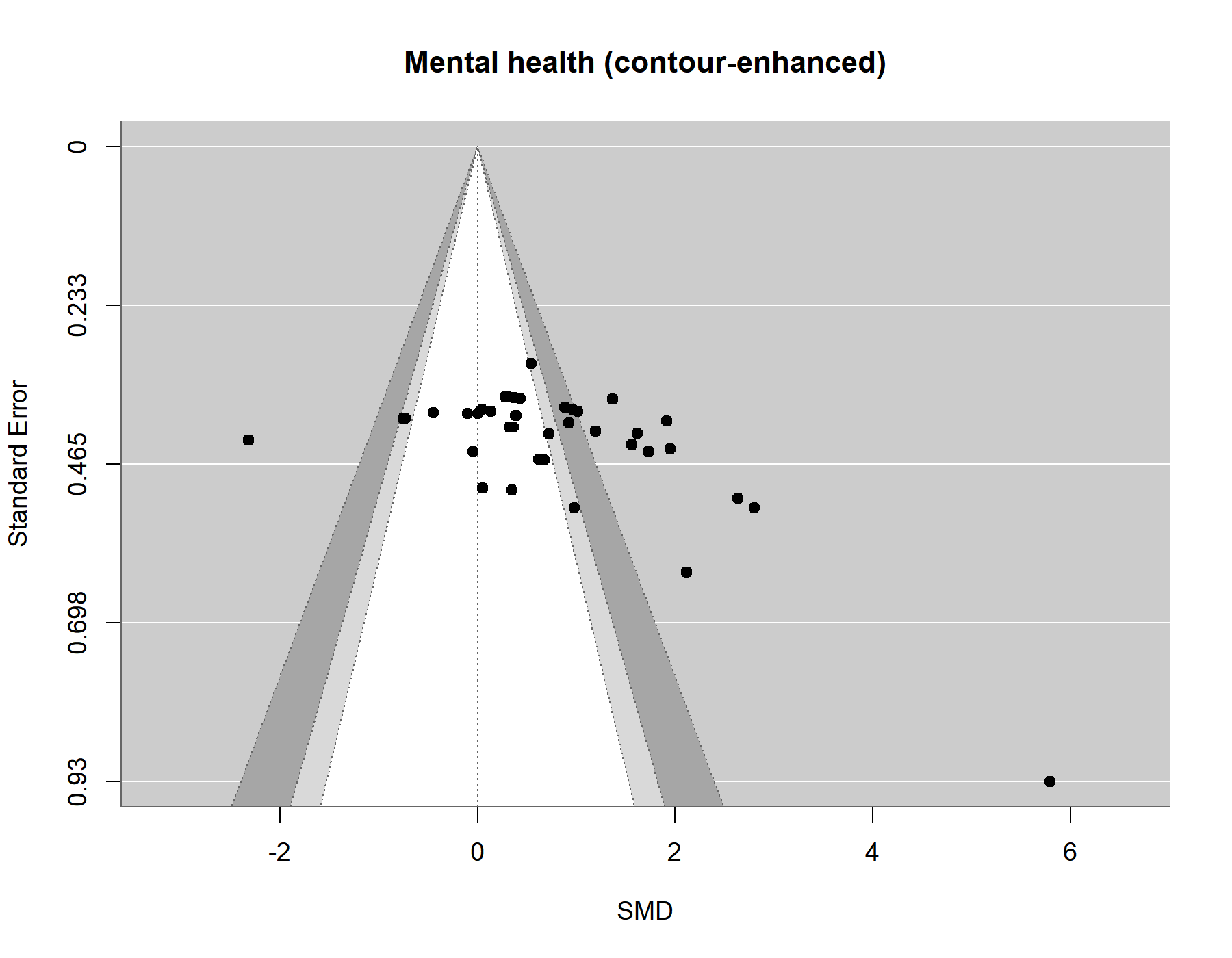


**Figure 50.** The Funnel Plot in Mental health (contour-enhanced)


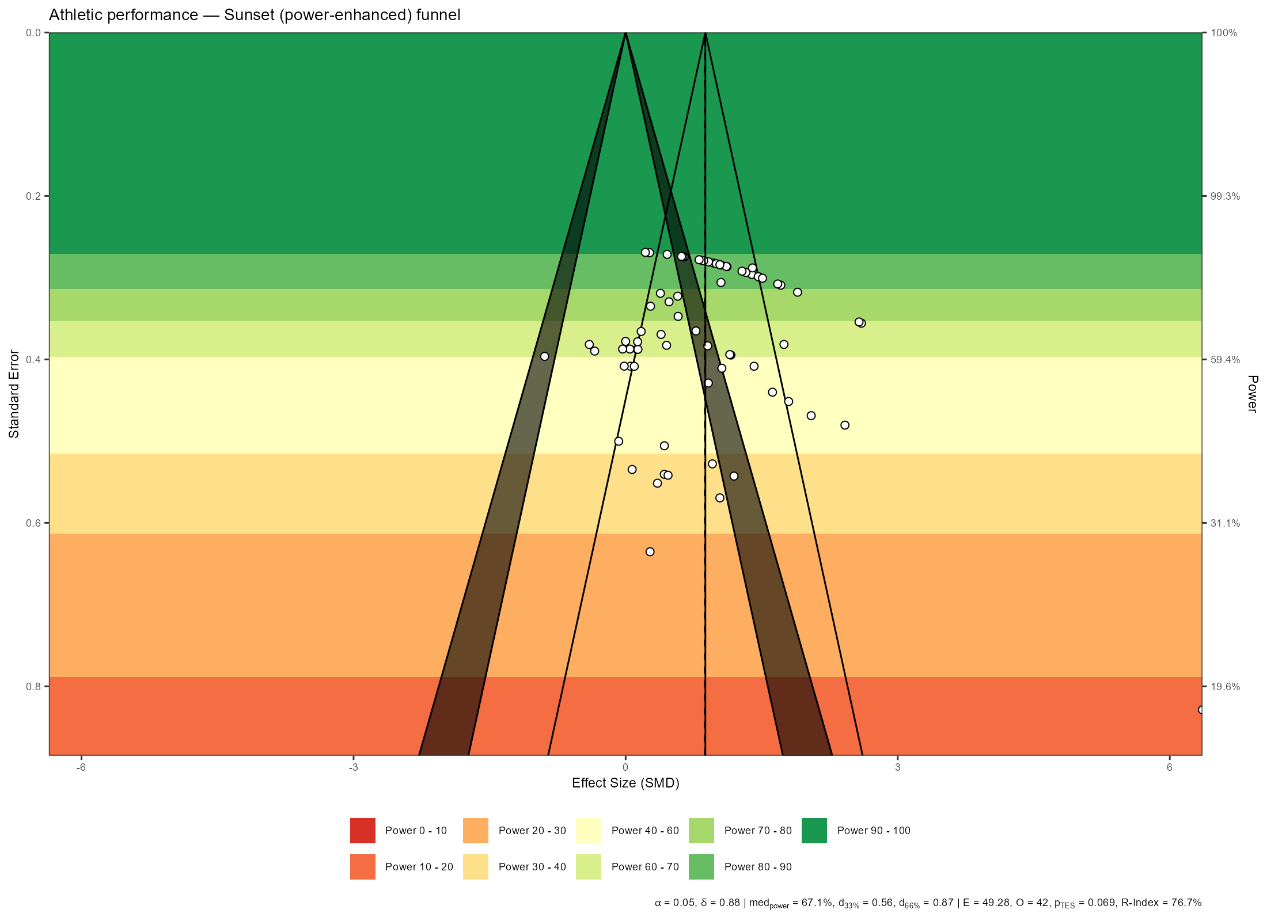


**Figure 51.** The Funnel Plot in Athletic performance Sunset (power-enhanced) funnel


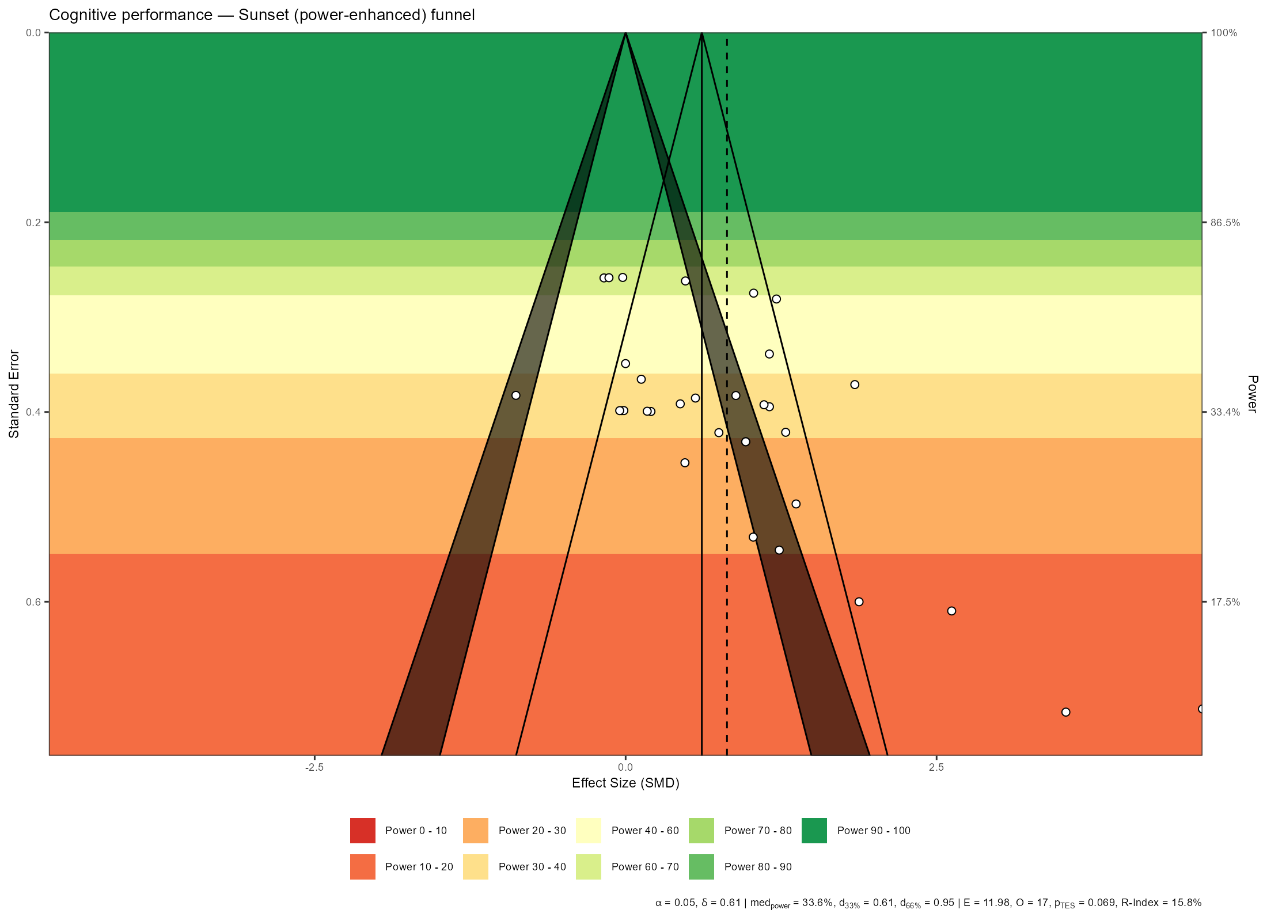


**Figure 52.** The Funnel Plot in Cognitive performance Sunset (power-enhanced) funnel


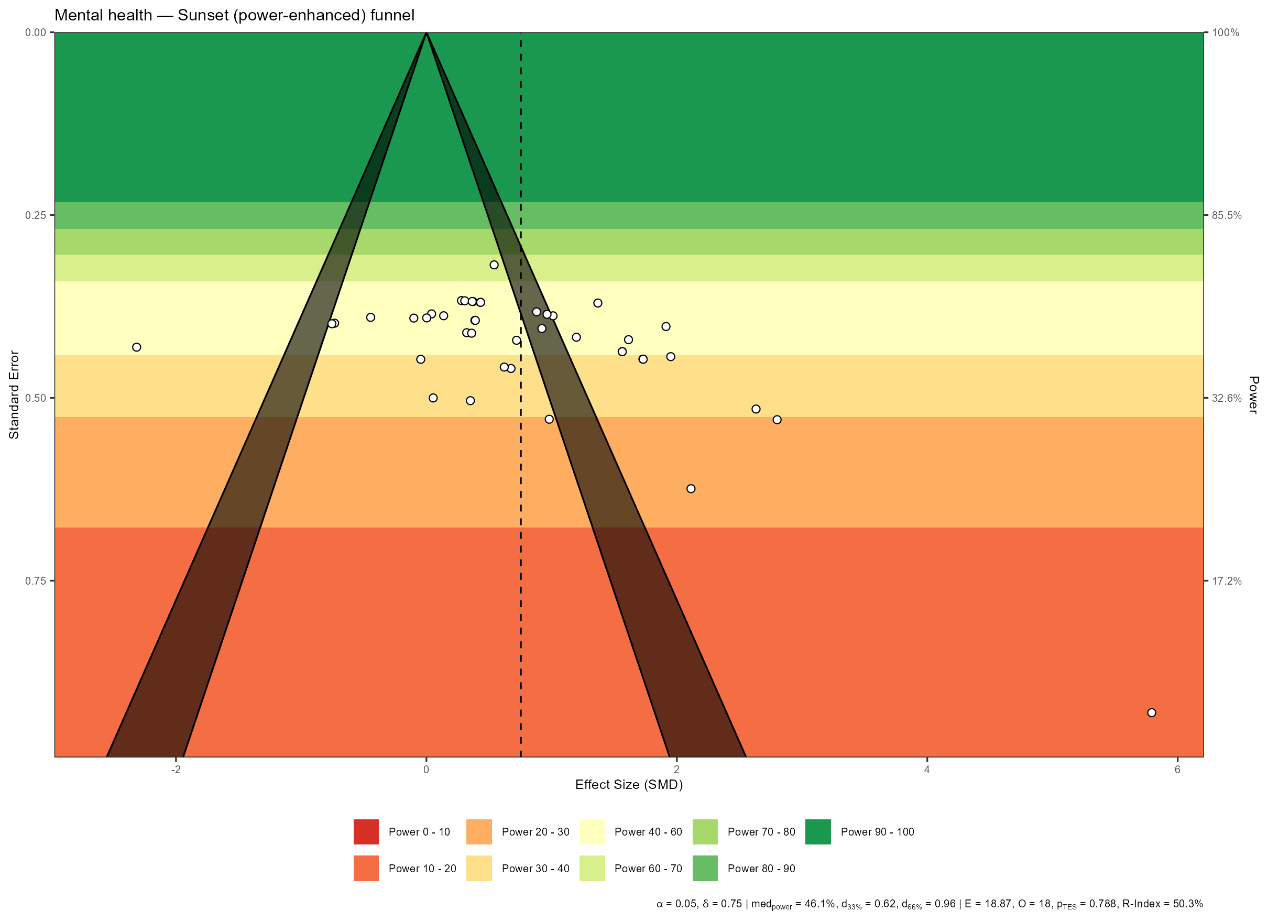
 **Figure 53.** The Funnel Plot in Mental health Sunset (power-enhanced) funnel
