# Supplementary material for: The effects of biofeedback training on athletes’ mental health and performance: a systematic review and Bayesian meta-analysis
Source: Front Psychol. 2025 Oct 21;16:1662868. doi: 10.3389/fpsyg.2025.1662868 (PMC12583207; doi:10.3389/fpsyg.2025.1662868)
Supplement: Supplementary file 1 [file Data_Sheet_1.ZIP › Supplementary file S8 GRADE plots.docx]

**subgroup**


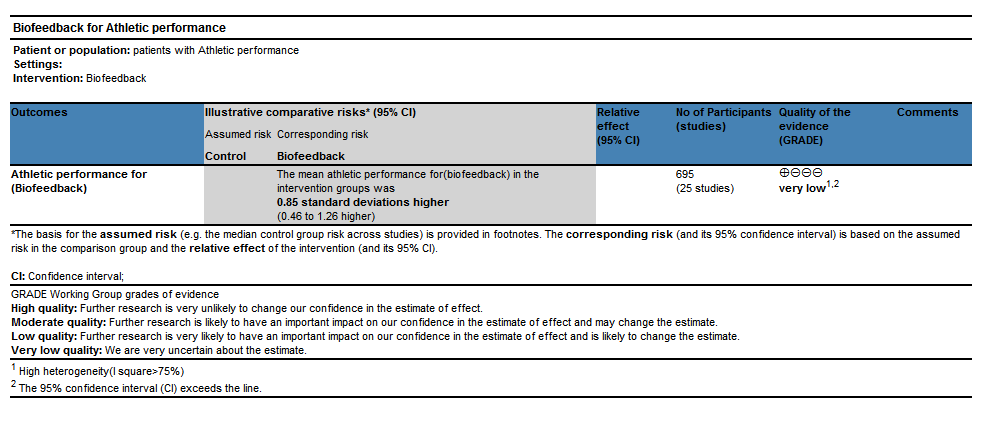


**Figure 1.** The GRADE Summary in Athletic Performance Biofeedback


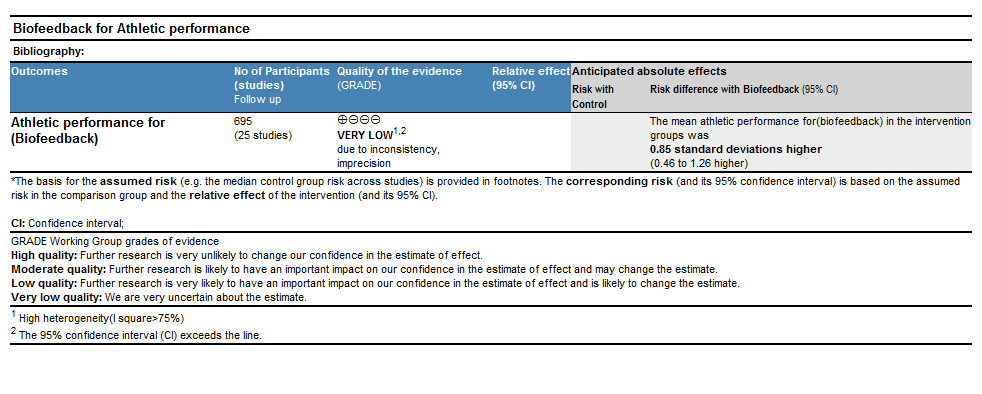


**Figure 2.** The GRADE Summary in Cognitive Performance Biofeedback


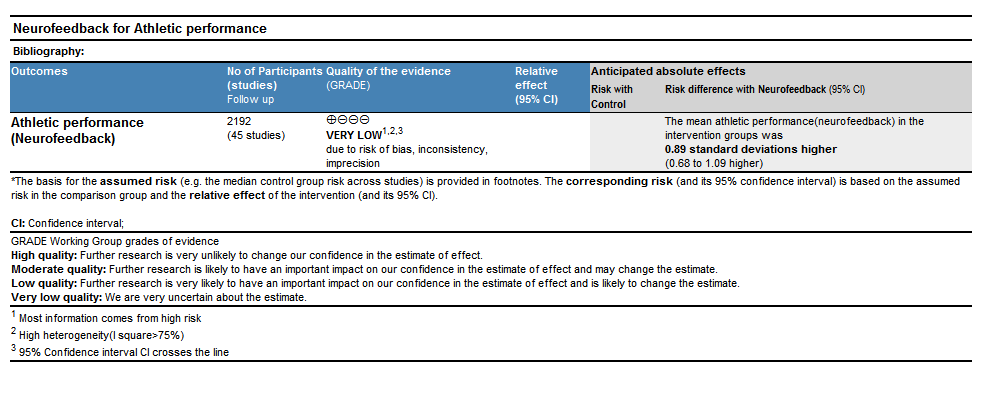


**Figure 3.** The GRADE Summary in Athletic Performance Neurofeedback


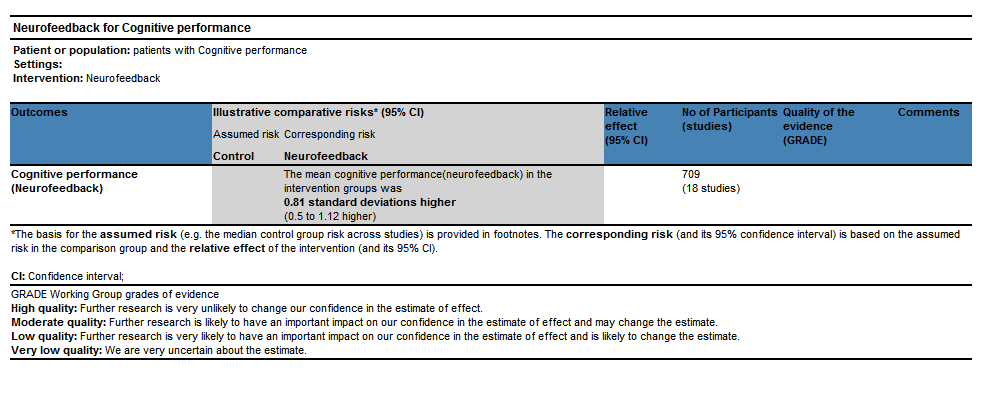


**Figure 4.** The GRADE Summary in Cognitive Performance Neurofeedback

**Detailed subgroups**


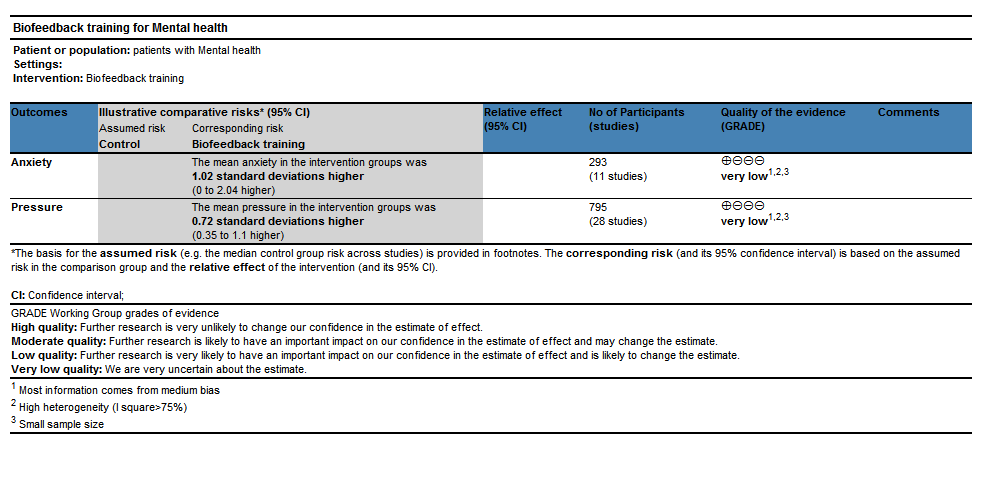


**Figure 5.** The GRADE Summary in Mental Health Biofeedback


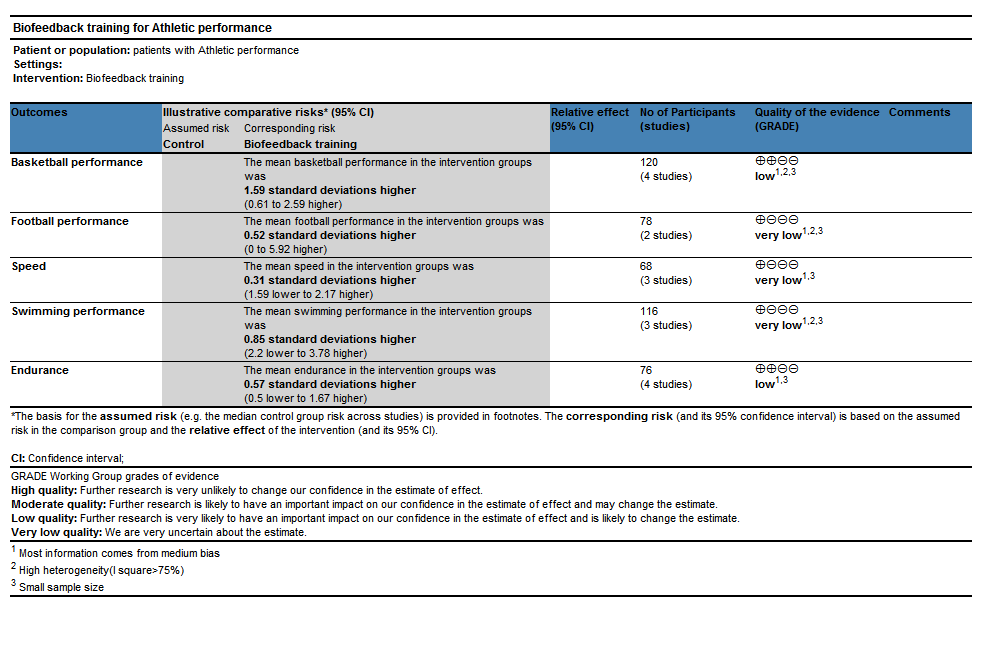


**Figure 6.** The GRADE Summary in Athletic Performance Biofeedback


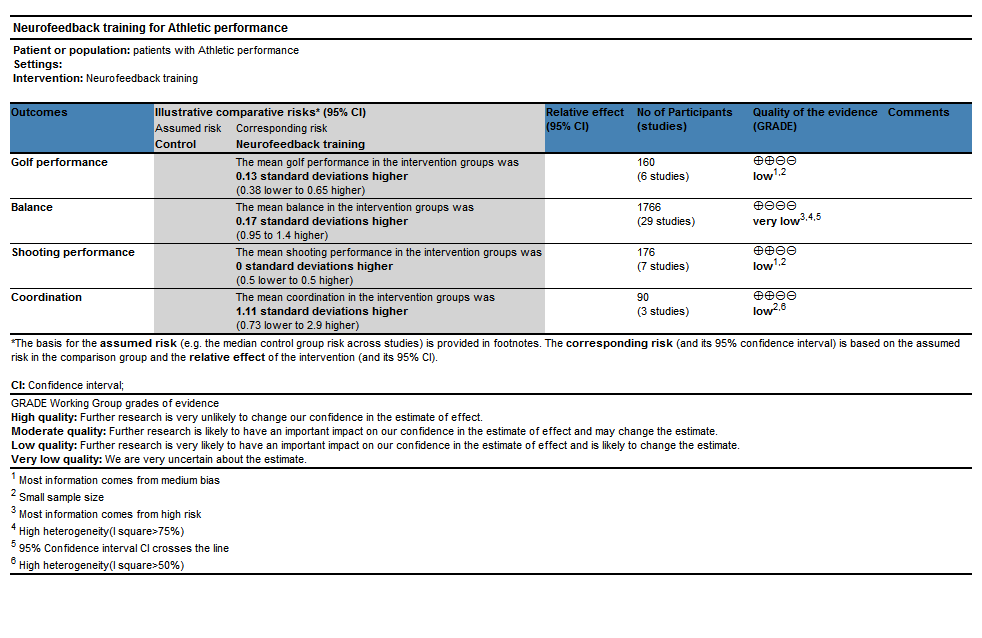


**Figure 7.** The GRADE Summary in Athletic Performance Neurofeedback


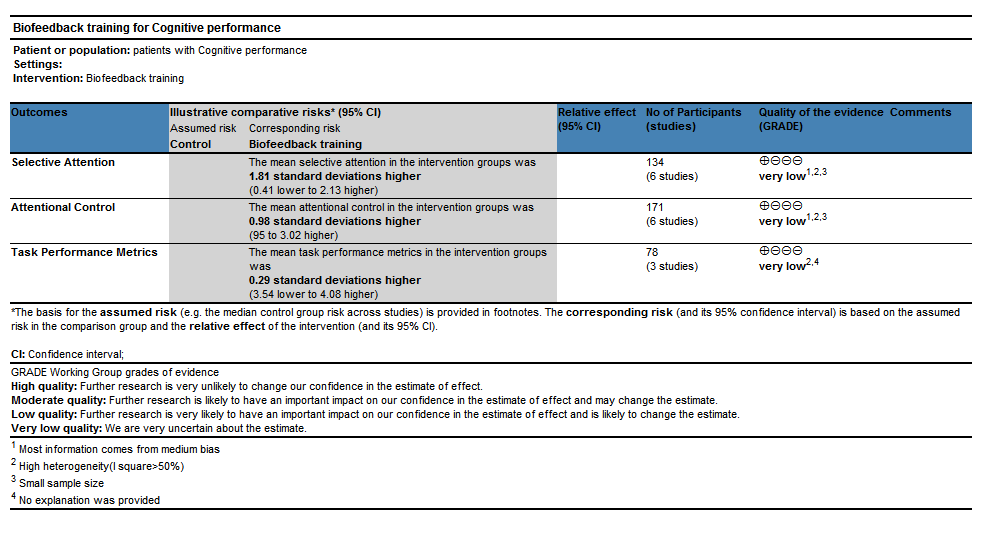


**Figure 8.** The GRADE Summary in Cognitive Performance Biofeedback


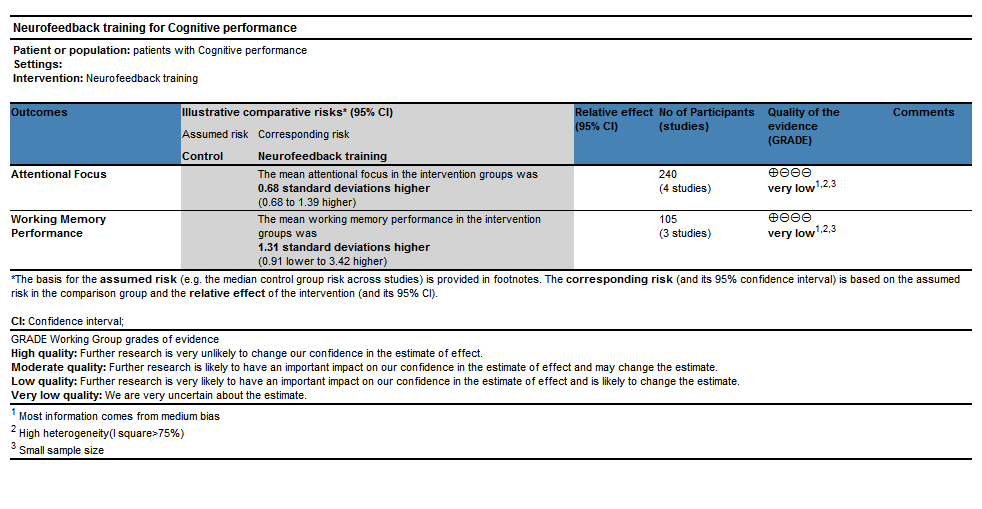


**Figure 9.** The GRADE Summary in Cognitive Performance Neurofeedback
