# Supplementary material for: The effects of biofeedback training on athletes’ mental health and performance: a systematic review and Bayesian meta-analysis
Source: Front Psychol. 2025 Oct 21;16:1662868. doi: 10.3389/fpsyg.2025.1662868 (PMC12583207; doi:10.3389/fpsyg.2025.1662868)
Supplement: Supplementary file 2 [file Data_Sheet_2.ZIP › Figure 9.pdf]

**Biofeedback & Neurofeedback for Mental health and performance**

**Patient or population:** patients with Mental health and performance
**Settings:**
**Intervention:** Biofeedback & Neurofeedback

| Outcomes | Illustrative comparative risks* (95% CI) | | Relative effect (95% CI) | No of Participants (studies) | Quality of the evidence (GRADE) | Comments |
|---|---|---|---|---|---|---|
| | Assumed risk | Corresponding risk | | | | |
| | Control | Biofeedback & Neurofeedback | | | | |
| **Mental health（Biofeedback & Neurofeedback）** | | The mean mental health（biofeedback & neurofeedback） in the intervention groups was **0.76 standard deviations higher** (0.44 to 1.09 higher) | | 1176 (15 studies) | ⊕⊕⊖⊖ **low**[1,2,3] | |
| **Athletic performance （Biofeedback & Neurofeedback)** | | The mean athletic performance （biofeedback & neurofeedback) in the intervention groups was **0.88 standard deviations higher** (0.09 to 0.69 higher) | | 2847 (70 studies) | ⊕⊖⊖⊖ **very low**[1,2] | |
| **Cognitive performance（Biofeedback & Neurofeedback)** | | The mean cognitive performance（biofeedback & neurofeedback) in the intervention groups was **0.81 standard deviations higher** (0 to 1.15 higher) | | 1152 (36 studies) | ⊕⊖⊖⊖ **very low**[1,2,3] | |

*The basis for the **assumed risk** (e.g. the median control group risk across studies) is provided in footnotes. The **corresponding risk** (and its 95% confidence interval) is based on the assumed risk in the comparison group and the **relative effect** of the intervention (and its 95% CI).

**CI:** Confidence interval;

GRADE Working Group grades of evidence
**High quality:** Further research is very unlikely to change our confidence in the estimate of effect.
**Moderate quality:** Further research is likely to have an important impact on our confidence in the estimate of effect and may change the estimate.
**Low quality:** Further research is very likely to have an important impact on our confidence in the estimate of effect and is likely to change the estimate.
**Very low quality:** We are very uncertain about the estimate.

[1] Most information comes from medium bias
[2] High heterogeneity (I square>75%)
[3] Small sample size
