# Supplementary figures and images for: The effects of biofeedback training on athletes’ mental health and performance: a systematic review and Bayesian meta-analysis
Source: Front Psychol. 2025 Oct 21;16:1662868. doi: 10.3389/fpsyg.2025.1662868 (PMC12583207; doi:10.3389/fpsyg.2025.1662868)

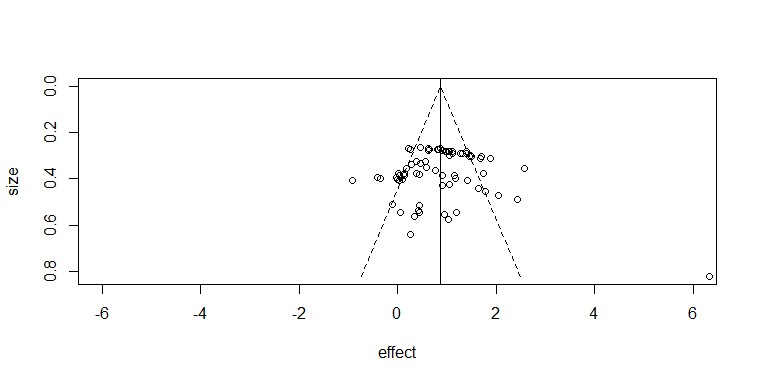

Supplement: Supplementary file 2 [file Data_Sheet_2.ZIP › Figure 7.jpg]

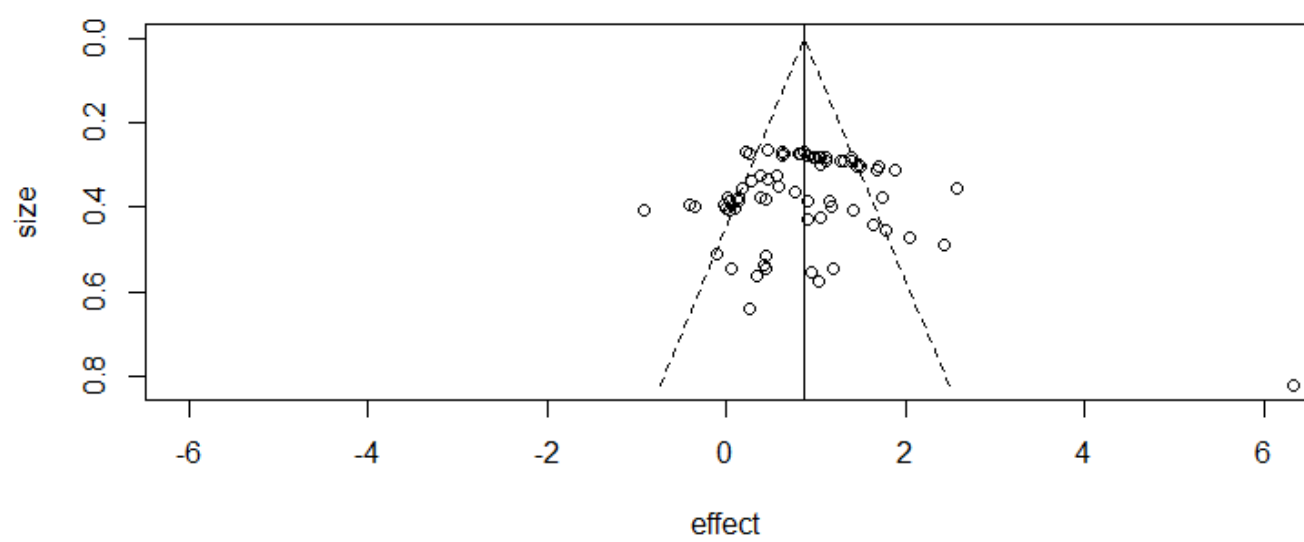

Supplement: Supplementary file 2 [file Data_Sheet_2.ZIP › Figure 7.pdf]

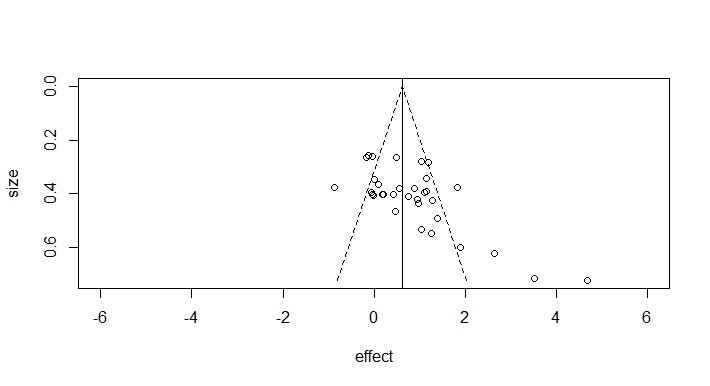

Supplement: Supplementary file 2 [file Data_Sheet_2.ZIP › Figure 8.jpg]

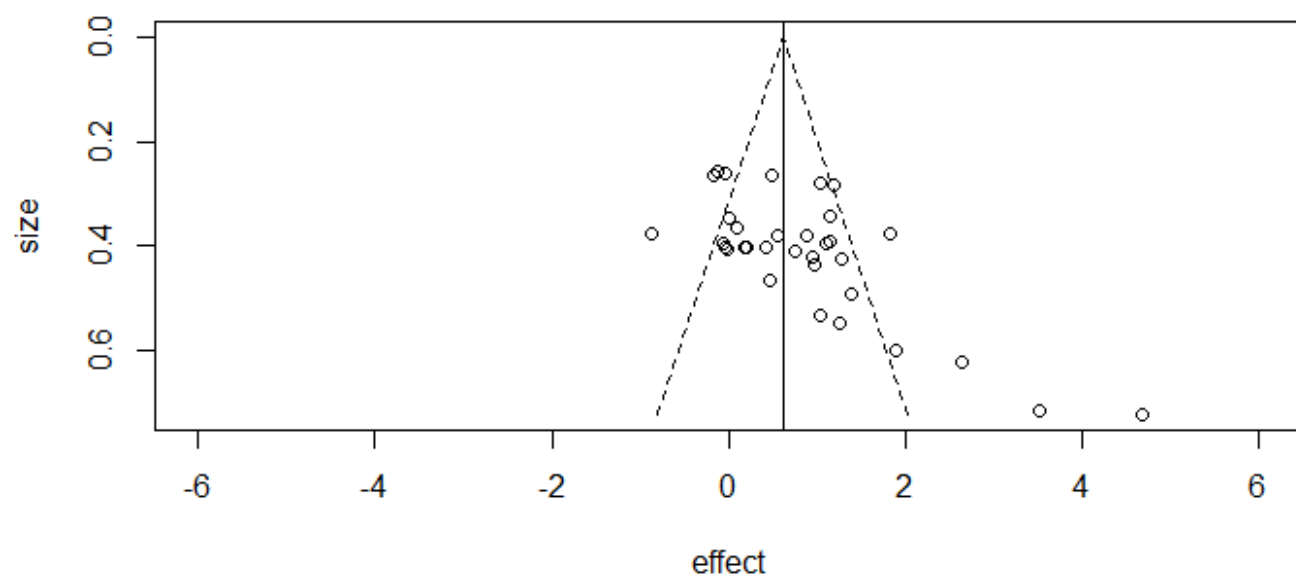

Supplement: Supplementary file 2 [file Data_Sheet_2.ZIP › Figure 8.pdf]

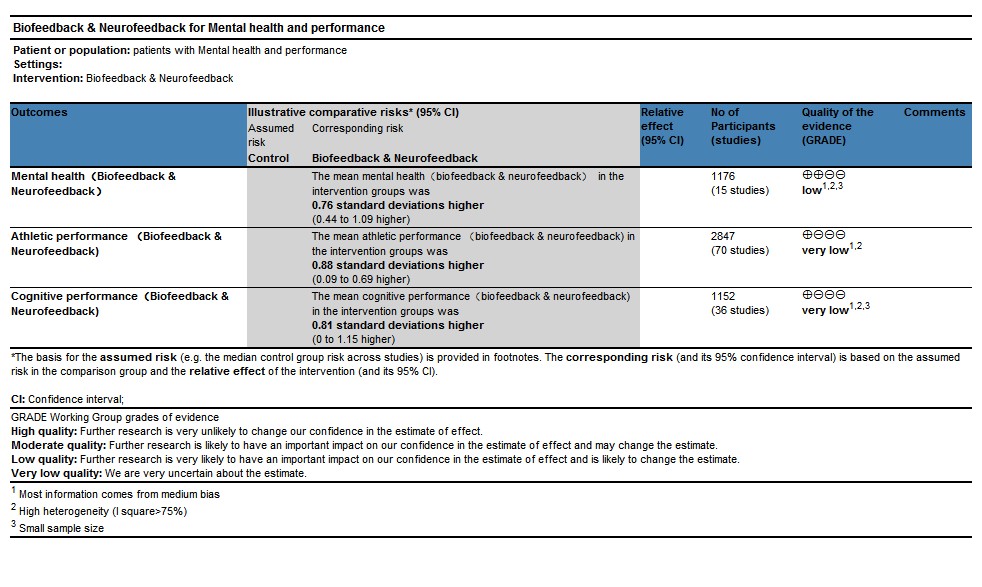

Supplement: Supplementary file 2 [file Data_Sheet_2.ZIP › Figure 9.jpg]

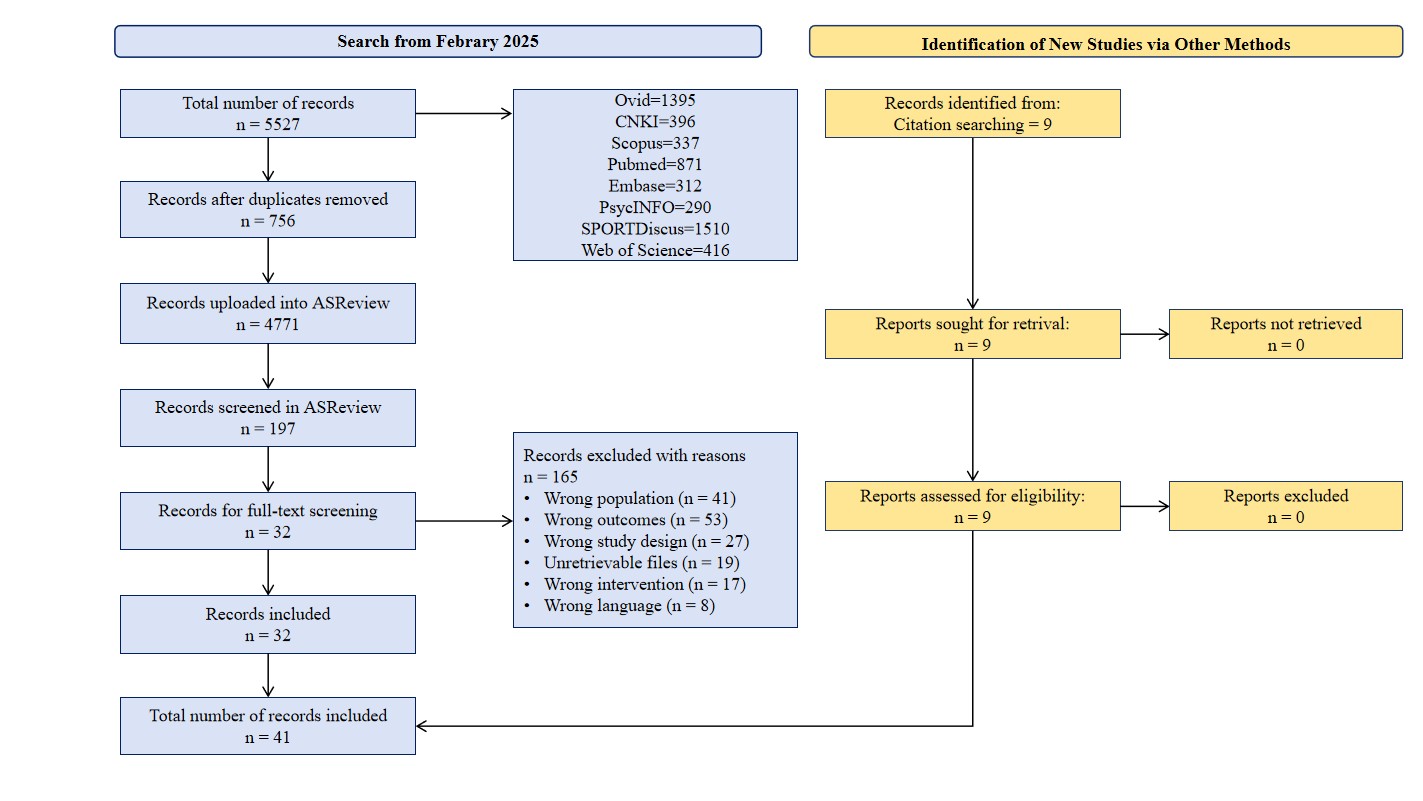

Supplement: Supplementary file 2 [file Data_Sheet_2.ZIP › Figure 1.jpg]

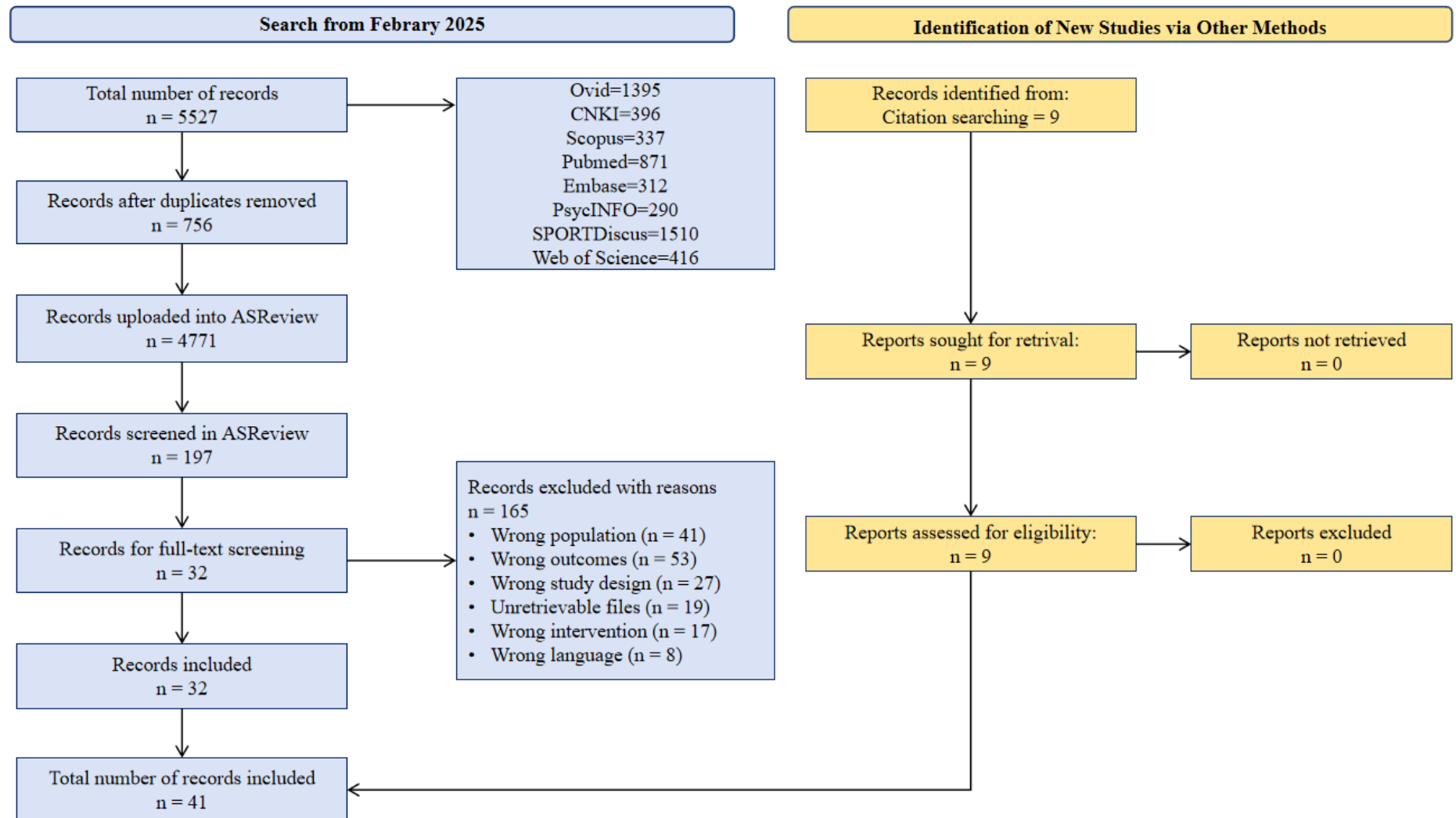

Supplement: Supplementary file 2 [file Data_Sheet_2.ZIP › Figure 1.pdf]

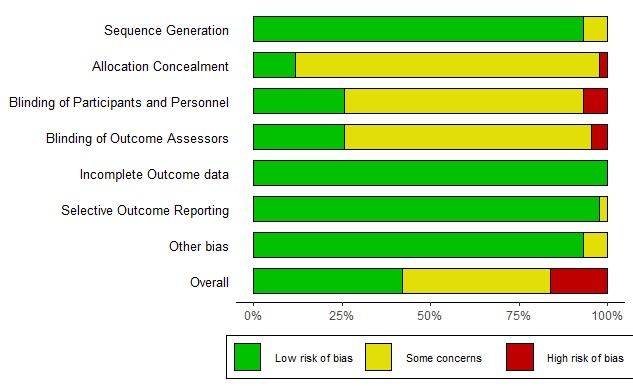

Supplement: Supplementary file 2 [file Data_Sheet_2.ZIP › Figure 2.jpg]

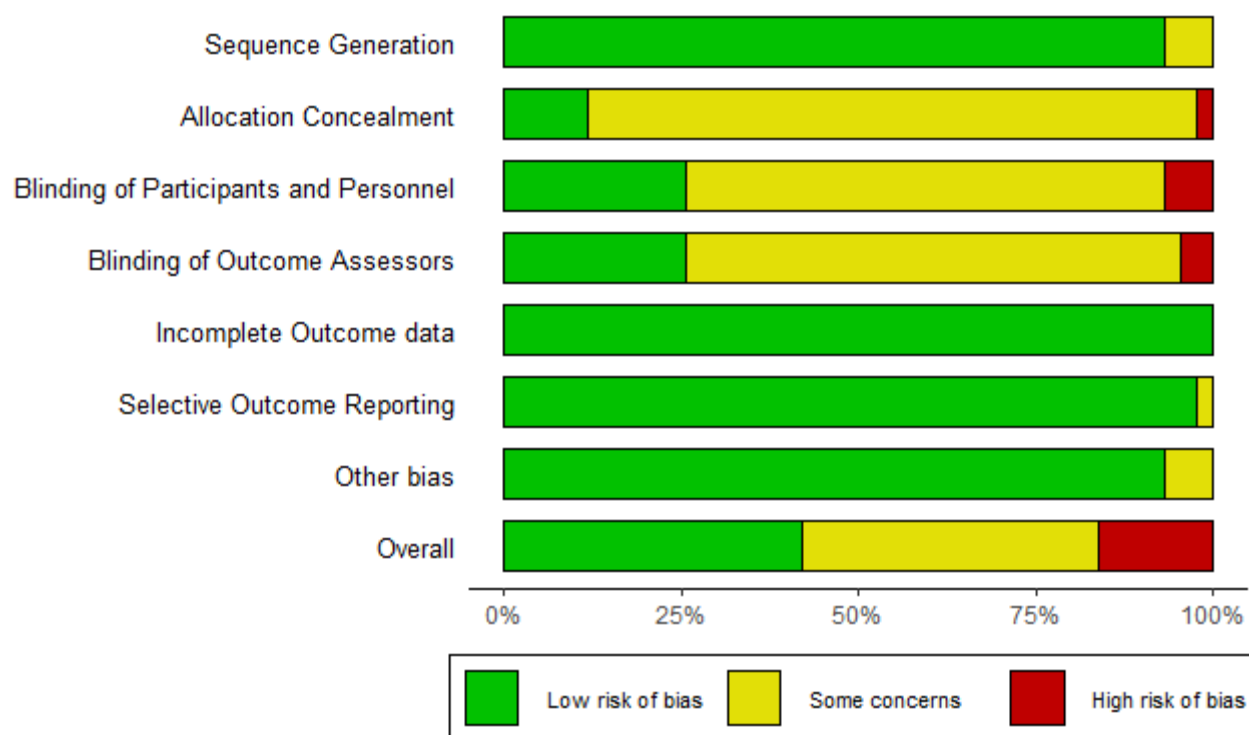

Supplement: Supplementary file 2 [file Data_Sheet_2.ZIP › Figure 2.pdf]

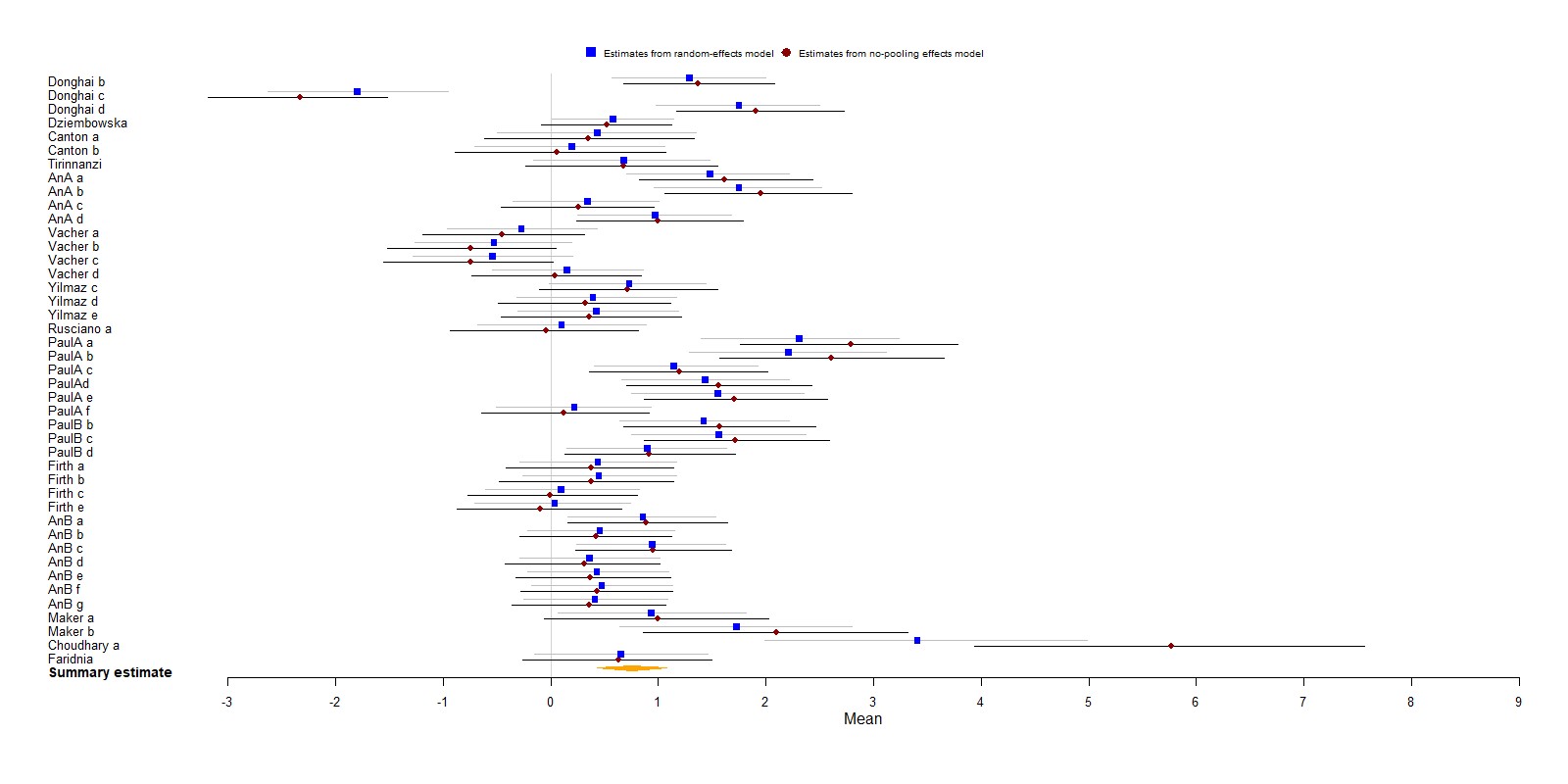

Supplement: Supplementary file 2 [file Data_Sheet_2.ZIP › Figure 3.jpg]

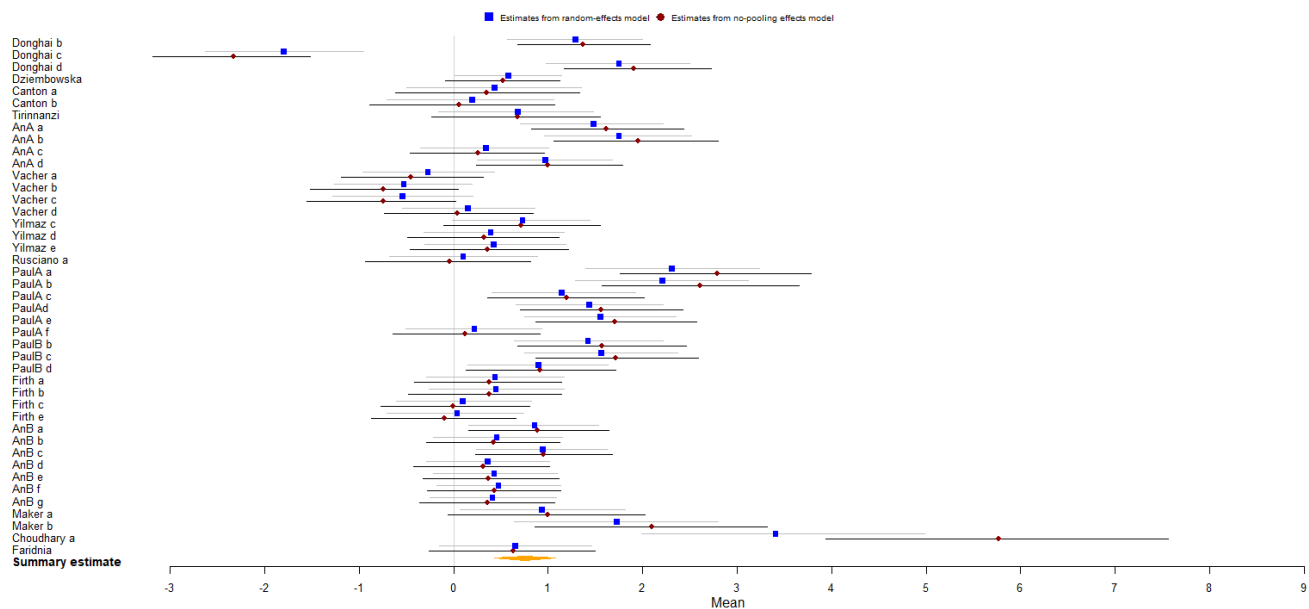

Supplement: Supplementary file 2 [file Data_Sheet_2.ZIP › Figure 3.pdf]

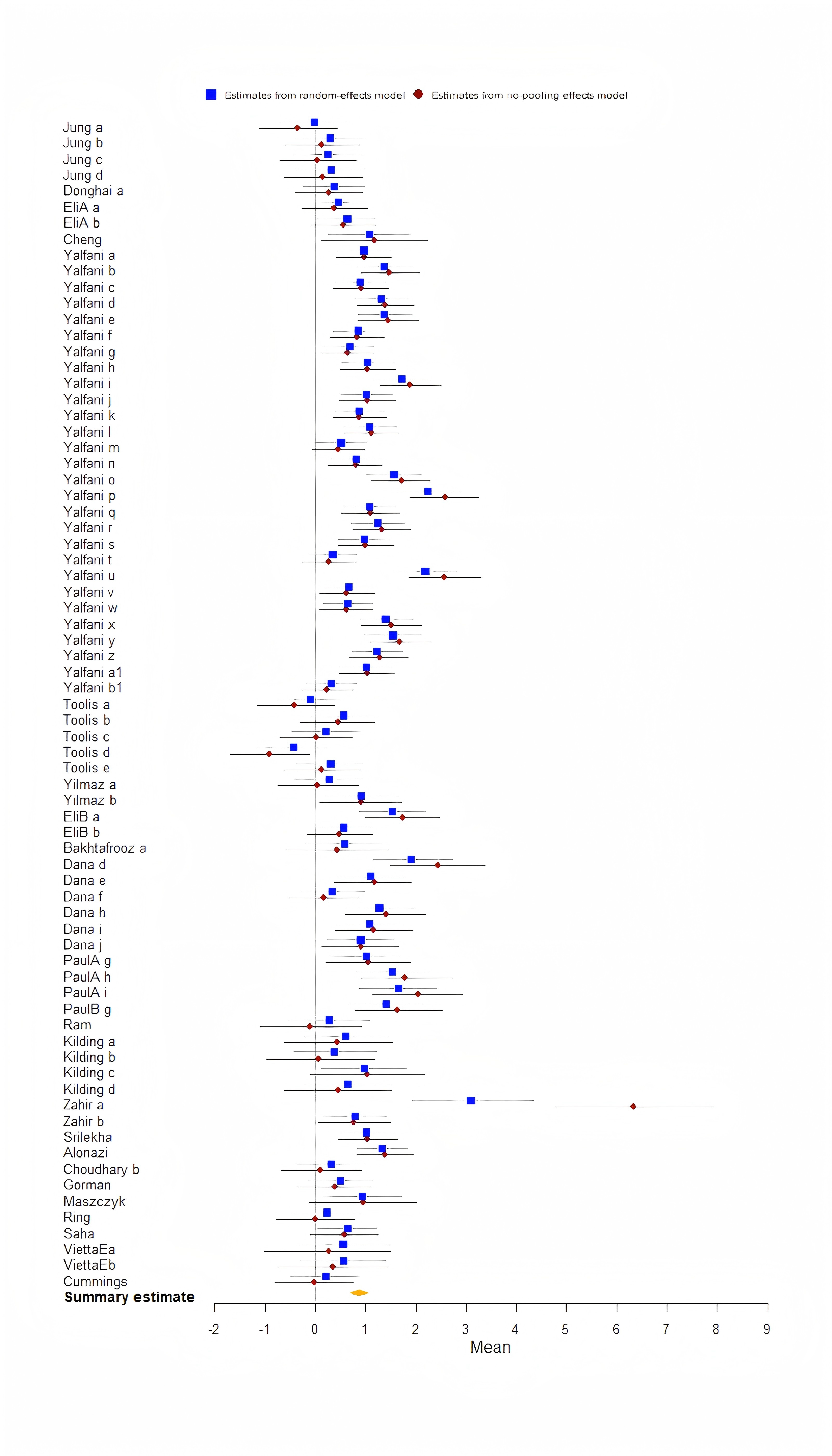

Supplement: Supplementary file 2 [file Data_Sheet_2.ZIP › Figure 4.jpg]

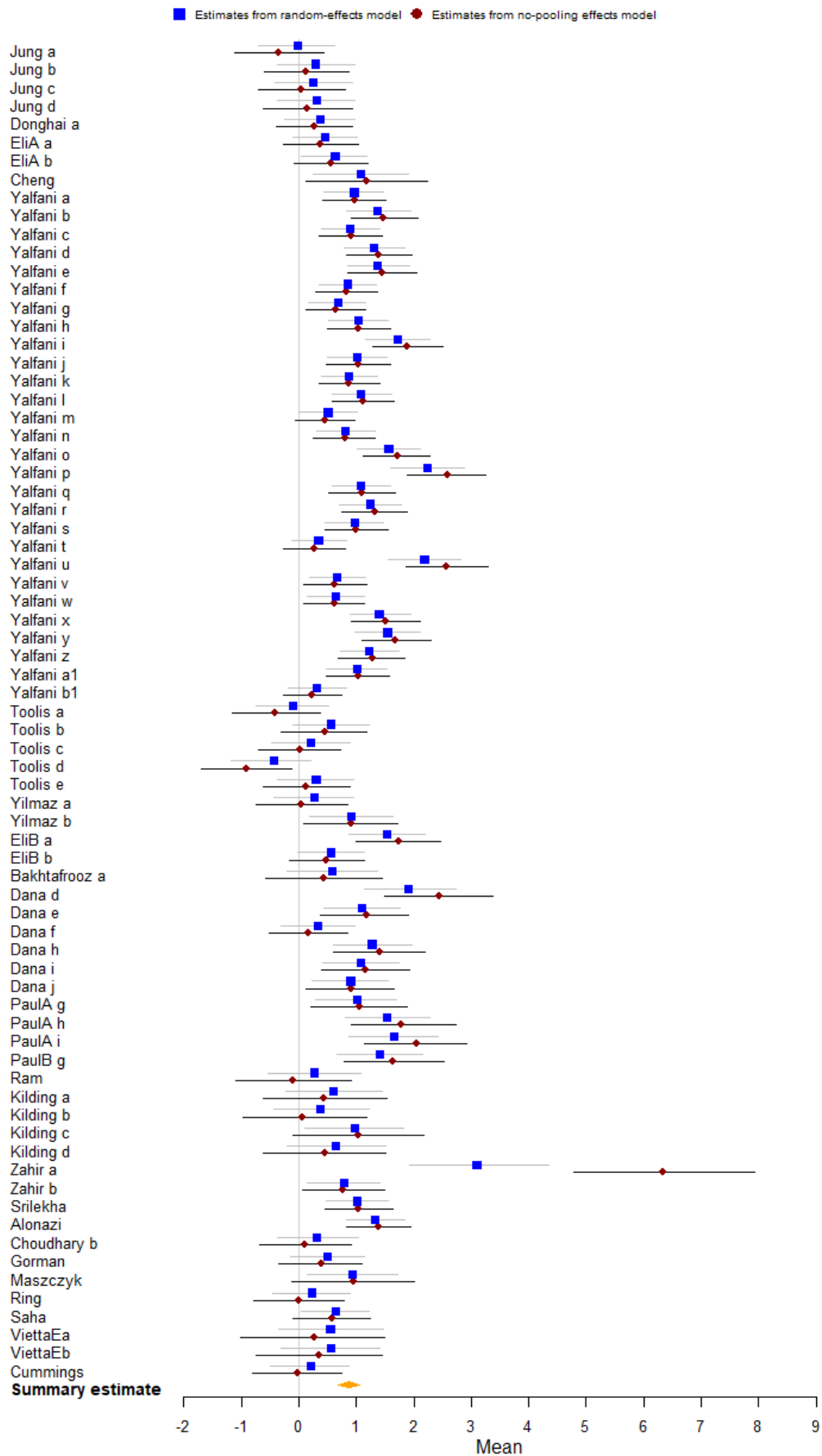

Supplement: Supplementary file 2 [file Data_Sheet_2.ZIP › Figure 4.pdf]

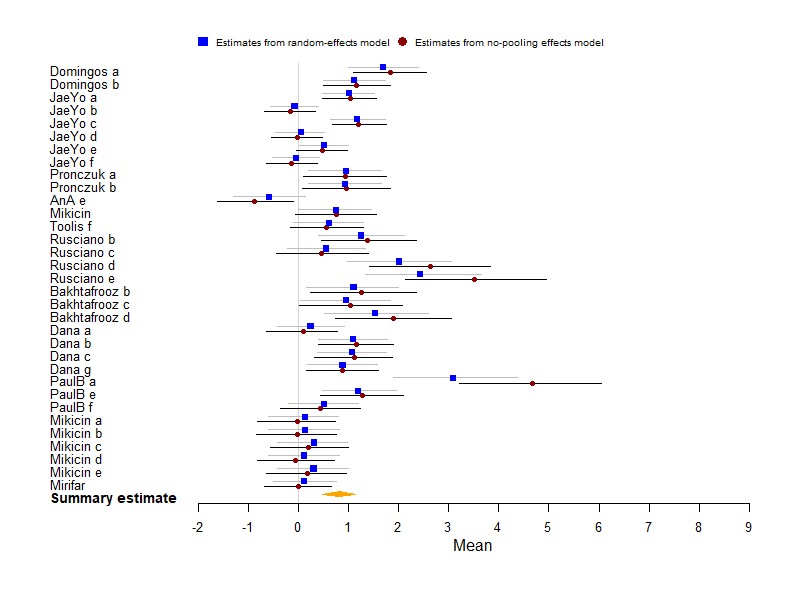

Supplement: Supplementary file 2 [file Data_Sheet_2.ZIP › Figure 5.jpg]

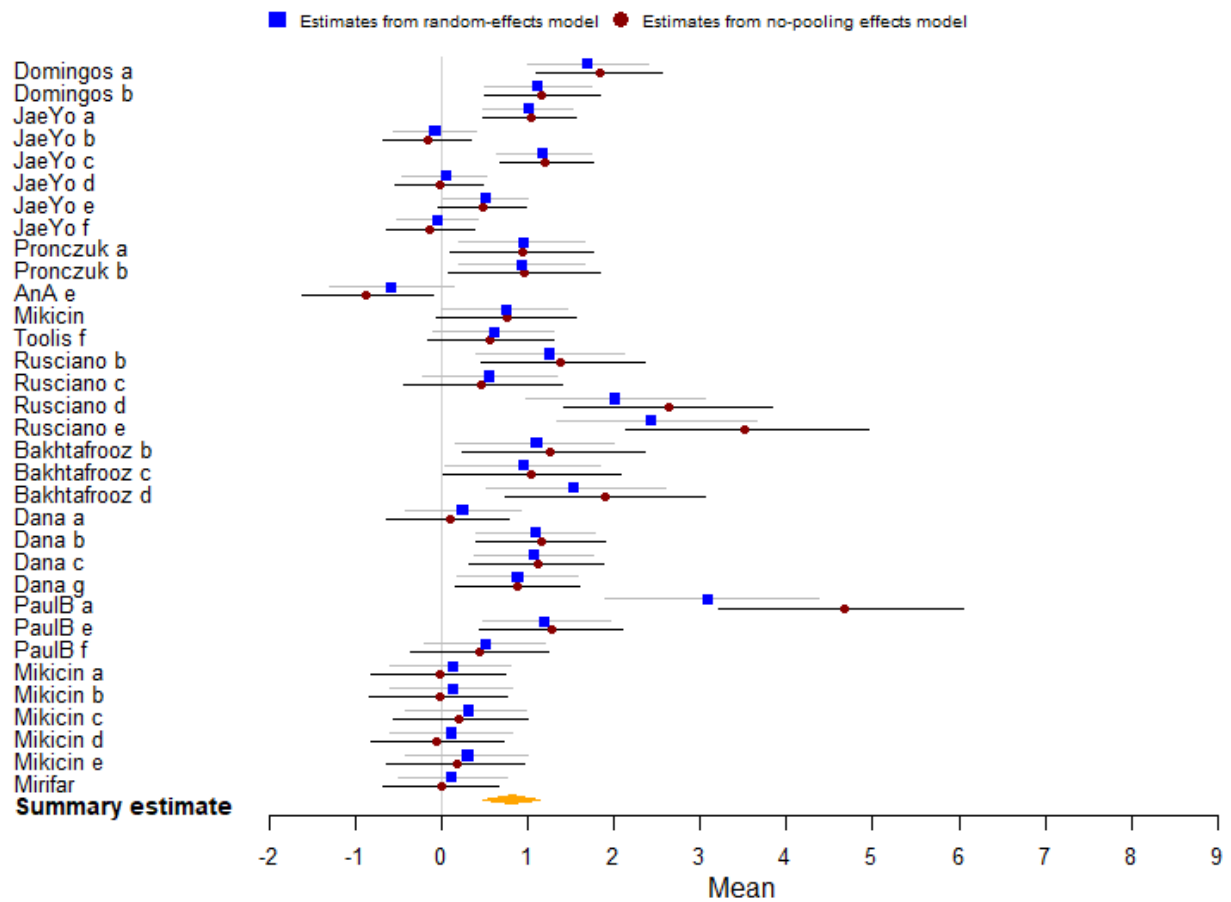

Supplement: Supplementary file 2 [file Data_Sheet_2.ZIP › Figure 5.pdf]

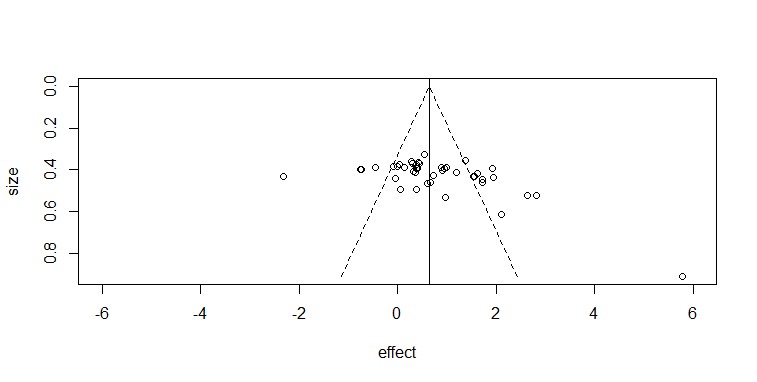

Supplement: Supplementary file 2 [file Data_Sheet_2.ZIP › Figure 6.jpg]

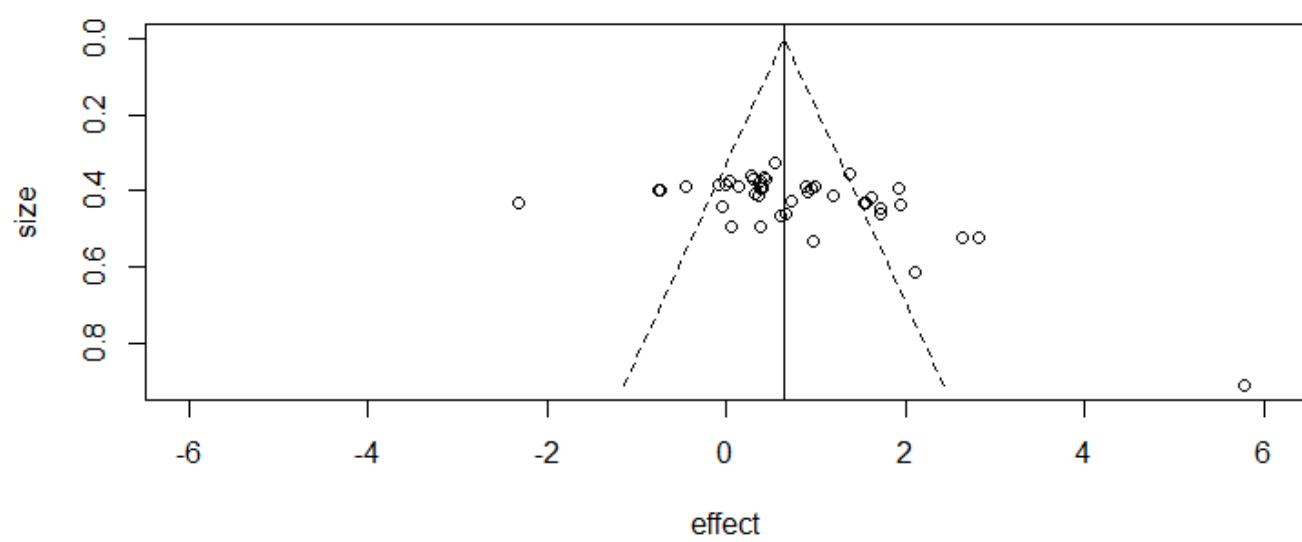

Supplement: Supplementary file 2 [file Data_Sheet_2.ZIP › Figure 6.pdf]

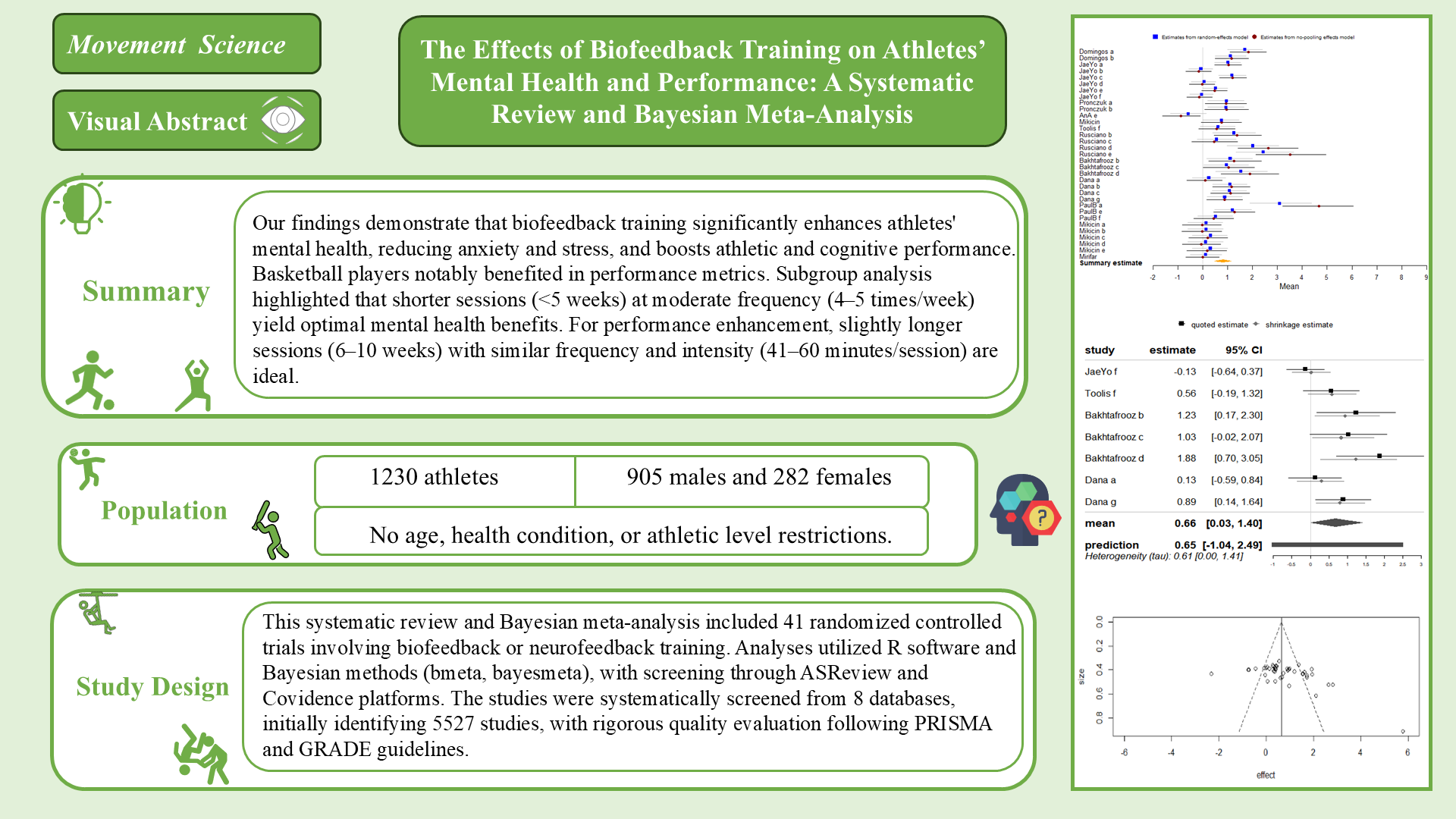

Supplement: Supplementary file 3 [file Image_1.PNG]
